# Prospective and genetic analyses implicate lower immunosurveillance in the aetiology of prostate cancer

**DOI:** 10.1038/s41467-026-77378-3

**Published:** 2026-11-10

**Authors:** Mahboubeh Parsaeian, Wing Ching Chan, Joshua R. Atkins, Keren Papier, Marie Breeur, Trishna A. Desai, Zhe Huang, Chibuzor F. Ogamba, David V. Conti, David Bogumil, Jiayi Shen, Malcolm J. W. Sim, Konstantinos K. Tsilidis, James Yarmolinsky, Sabina Rinaldi, Rudolf Kaaks, Verena Katzke, Matthias B. Schulze, Catarina Schiborn, Saverio Caini, Lorenzo Milani, Raul Zamora-Ros, Marcela Guevara, Maria-Jose Sánchez, María-Dolores Chirlaque, Pilar Amiano, The PRACTICAL Consortium, Rosalind A. Eeles, Christopher A. Haiman, Zsofia Kote-Jarai, Fredrick R. Schumacher, Sara Benlloch, Ali Amin Al Olama, Kenneth R. Muir, Sonja I. Berndt, David V. Conti, Fredrik Wiklund, Stephen Chanock, Ying Wang, Catherine M. Tangen, Jyotsna Batra, Judith A. Clements, Timothy Elliott, Ian G. Mills, Elio Riboli, Loïc Le Marchand, Christopher Haiman, Tim J. Key, Karl Smith-Byrne, Ruth C. Travis

**Affiliations:** 1 Cancer Epidemiology Unit, Nuffield Department of Population Health, University of Oxford, Oxford, UK; 2 Department of Biostatistics and Informatics, Colorado School of Public Health, Aurora, CO USA; 3 Department of Population and Public Health Science, University of Southern California, Los Angeles, CA USA; 4 Oxford Cancer Centre for Immuno-oncology, Nuffield Department of Medicine, University of Oxford, Oxford, UK; 5 Department of Epidemiology and Biostatistics, School of Public Health, Imperial College London, London, UK; 6 Department of Hygiene and Epidemiology, University of Ioannina School of Medicine, Ioannina, Greece; 7 Population Health Sciences, Bristol Medical School, University of Bristol, Bristol, UK; 8 International Agency for Research on Cancer (IARC/WHO), Nutrition and Metabolism Branch, Lyon, France; 9 German Cancer Research Center (DKFZ), Division of Cancer Epidemiology, Heidelberg, Germany; 10 Department of Molecular Epidemiology, German Institute of Human Nutrition Potsdam-Rehbruecke, Nuthetal, Germany; 11 Institute of Nutritional Science, University of Potsdam, Nuthetal, Germany; 12 German Center for Diabetes Research (DZD), München-Neuherberg, Germany; 13 Institute for Cancer Research, Prevention, and Clinical Network (ISPRO), Cancer Risk Factors and Lifestyle Epidemiology Unit, Florence, Italy; 14 Centre for Biostatistics, Epidemiology, and Public Health, Department of Clinical and Biological Sciences,University of Turin, Orbassano, Turin, Italy; 15 Unit of Nutrition and Cancer, Cancer Epidemiology Research Programme, Catalan Institute of Oncology (ICO), Bellvitge Biomedical Research Institute (IDIBELL), Barcelona, Spain; 16 Instituto de Salud Pública y Laboral de Navarra, Pamplona, Spain; 17 Centro de Investigación Biomédica en Red de Epidemiología y Salud Pública (CIBERESP), Madrid, Spain; 18 Navarra Institute for Health Research (IdiSNA), Pamplona, Spain; 19 Escuela Andaluza de Salud Pública (EASP), Granada, Spain; 20 Instituto de Investigación Biosanitaria ibs.GRANADA, Granada, Spain; 21 Department of Epidemiology, Regional Health Council, IMIB-Arrixaca, Murcia University, Murcia, Spain; 22 CIBER in Epidemiology and Public Health (CIBERESP), Madrid, Spain; 23 Ministry of Health of the Basque Government, Sub Directorate for Public Health and Addictions of Gipuzkoa, San Sebastian, Spain; 24 BioGipuzkoa (BioDonostia) Health Research Institute, Epidemiology of Chronic and Communicable Diseases Group, San Sebastián, Spain; 25 Nuffield Department of Surgical Sciences, University of Oxford, Oxford, UK; 26 Patrick G Johnston Centre for Cancer Research, Queen’s University Belfast, Belfast, UK; 27 University of Hawaii Cancer Center, Honolulu, HI USA; 28 Institute of Cancer Research, London, UK; 29 Royal Marsden NHS Foundation Trust, London, UK; 30 Center for Genetic Epidemiology, Department of Preventive Medicine, Keck School of Medicine, University of Southern California/Norris Comprehensive Cancer Center, Los Angeles, CA USA; 31 Department of Population and Quantitative Health Sciences, Case Western Reserve University, Cleveland, OH USA; 32 Seidman Cancer Center, University Hospitals, Cleveland, OH USA; 33 Centre for Cancer Genetic Epidemiology, Department of Public Health and Primary Care, University of Cambridge, Cambridge, UK; 34 University of Cambridge, Department of Clinical Neurosciences, Stroke Research Group, Cambridge, UK; 35 Division of Population Health, Health Services Research and Primary Care, University of Manchester, Manchester, UK; 36 Division of Cancer Epidemiology and Genetics, National Cancer Institute, Bethesda, MD USA; 37 Department of Medical Epidemiology and Biostatistics, Karolinska Institute, Stockholm, Sweden; 38 Department of Population Science, American Cancer Society, Atlanta, GA USA; 39 SWOG Statistical Center, Fred Hutchinson Cancer Research Center, Seattle, WA USA; 40 Australian Prostate Cancer Research Centre-QLD, Institute of Health and Biomedical Innovation and School of Biomedical Sciences, Queensland University of Technology, Brisbane, QLD Australia; 41 Translational Research Institute, Brisbane, QLD Australia; 42 Australian Prostate Cancer Research Centre-QLD, Queensland University of Technology, Brisbane, QLD Australia; 43 Prostate Cancer Research Program, Monash University, Melbourne, VIC Australia; 44 Dame Roma Mitchell Cancer Centre, University of Adelaide, Adelaide, SA Australia; 45 Chris O’Brien Lifehouse, Royal Prince Alfred Hospital, Camperdown, VIC Australia; 46 Department of Applied Health Research, University College London, London, UK; 47 Centre for Cancer Genetic Epidemiology, Department of Oncology, University of Cambridge, Cambridge, UK; 48 Institute of Biomedicine, University of Turku, Turku, Finland; 49 Department of Medical Genetics, Turku University Hospital, Turku, Finland; 50 Department of Surgical Sciences, Uppsala University, Uppsala, Sweden; 51 Division of Cancer Sciences, University of Manchester, Manchester Academic Health Science Centre, Radiotherapy Related Research, Christie Hospital NHS Foundation Trust, Manchester, UK; 52 Department of Epidemiology, Harvard T.H. Chan School of Public Health, Boston, MA USA; 53 CeRePP, Tenon Hospital, Paris, France; 54 Sorbonne Universite, GRC n°5, AP-HP, Tenon Hospital, Paris, France; 55 Department of Molecular Medicine, Aarhus University Hospital, Aarhus N, Denmark; 56 Department of Clinical Medicine, Aarhus University, Aarhus N, Denmark; 57 Department of Medical Genetics, Oslo University Hospital, Oslo, Norway; 58 Nuffield Department of Surgical Sciences, University of Oxford, John Radcliffe Hospital, Oxford, UK; 59 University of Cambridge, Department of Oncology, Addenbrooke’s Hospital, Cambridge, UK; 60 Cancer Research UK, Cambridge Research Institute, Li Ka Shing Centre, Cambridge, UK; 61 Faculty of Medical Science, University of Oxford, John Radcliffe Hospital, Oxford, UK; 62 Dept. of Surgical Oncology, Princess Margaret Cancer Centre, Toronto, ON Canada; 63 Dept. of Surgery (Urology), University of Toronto, Toronto, ON Canada; 64 Department of Preventive Medicine, Keck School of Medicine, University of Southern California/Norris Comprehensive Cancer Center, Los Angeles, CA USA; 65 Department of Radiation Oncology and Department of Genetics and Genomic Sciences, Icahn School of Medicine at Mount Sinai, New York, NY USA; 66 Centre for Cancer Biomarker and Biotherapeutics, Barts Cancer Institute, Queen Mary University of London, John Vane Science Centre, London, UK; 67 Cancer Epidemiology Division, Cancer Council Victoria, Melbourne, VIC Australia; 68 Centre for Epidemiology and Biostatistics, Melbourne School of Population and Global Health, University of Melbourne, Parkville, VIC Australia; 69 Precision Medicine, School of Clinical Sciences at Monash Health, Monash University, Clayton, VIC Australia; 70 Division of Urologic Surgery, Brigham and Womens Hospital, Boston, MA USA; 71 Fundación Pública Galega Medicina Xenómica, Santiago de Compostela, Compostela, Spain; 72 Instituto de Investigación Sanitaria de Santiago de Compostela, Santiago De Compostela, Spain; 73 Centro de Investigación en Red de Enfermedades Raras, Madrid, Spain; 74 ISGlobal, Barcelona, Spain; 75 IMIM (Hospital del Mar Medical Research Institute), Barcelona, Spain; 76 Universitat Pompeu Fabra, Barcelona, Spain; 77 CIBER Epidemiología y Salud Pública, Madrid, Spain; 78 Channing Division of Network Medicine, Department of Medicine, Brigham and Women’s Hospital/Harvard Medical School, Boston, MA USA; 79 Department of Cancer Epidemiology, Moffitt Cancer Center, Tampa, FL USA; 80 Division of Public Health Sciences, Fred Hutchinson Cancer Research Center, Seattle, WA USA; 81 Department of Epidemiology, School of Public Health, University of Washington, Seattle, WA USA; 82 International Hereditary Cancer Center, Department of Genetics and Pathology, Pomeranian Medical University, Szczecin, Poland; 83 Faculty of Health and Medical Sciences, University of Copenhagen, Copenhagen, Denmark; 84 Department of Clinical Biochemistry, Herlev and Gentofte Hospital, Copenhagen University Hospital, Herlev, Copenhagen, Denmark; 85 Division of Clinical Epidemiology and Aging Research, German Cancer Research Center, Heidelberg, Germany; 86 German Cancer Consortium, German Cancer Research Center Heidelberg, Heidelberg, Germany; 87 Division of Preventive Oncology, German Cancer Research Center and National Center for Tumor Diseases, Heidelberg, Germany; 88 Humangenetik Tuebingen, Tuebingen, Germany; 89 University of Texas M.D. Anderson Cancer Center, Department of Genitourinary Medical Oncology, Houston, TX USA; 90 Departments of Epidemiology & Population Health and of Medicine, Division of Oncology, Stanford Cancer Institute, Stanford University School of Medicine, Stanford, CA USA; 91 Department of Genetics, Portuguese Oncology Institute of Porto, Porto, Portugal; 92 Biomedical Sciences Institute, University of Porto, Porto, Portugal; 93 Cancer Genetics Group, IPO-Porto Research Center, Portuguese Oncology Institute of Porto, Porto, Portugal; 94 Department of Population Sciences, Beckman Research Institute of City of Hope, Duarte, CA USA; 95 Ghent University, Faculty of Medicine and Health Sciences, Basic Medical Sciences, Ghent, Belgium; 96 Department of Surgery, Faculty of Medicine, University of Malaya, Kuala Lumpur, Malaysia; 97 Department of Urology, University of Washington, Seattle, WA USA; 98 Institute of Human Genetics, University Medical Center Hamburg-Eppendorf, Hamburg, Germany; 99 Molecular Medicine Center, Department of Medical Chemistry and Biochemistry, Medical University of Sofia, Sofia, Bulgaria; 100 Department of Oncology, Cross Cancer Institute, University of Alberta, Edmonton, AB Canada; 101 Division of Radiation Oncology, Cross Cancer Institute, Edmonton, AB Canada; 102 Molecular Endocrinology Laboratory, Department of Cellular and Molecular Medicine, KU Leuven, Leuven, Belgium; 103 Division of Cancer Sciences, Manchester Cancer Research Centre, Faculty of Biology, Medicine and Health, Manchester Academic Health Science Centre, NIHR Manchester Biomedical Research Centre, Health Innovation Manchester, University of Manchester, Manchester, UK; 104 University of Surrey, Guildford, UK; 105 Genetic Oncology Unit, Complexo Hospitalario Universitario de Vigo, Instituto de Investigación Biomédica Galicia Sur, Vigo (Pontevedra), Spain; 106 Department of Urology, Erasmus University Medical Center, Rotterdam, The Netherlands; 107 Exposome and Heredity, Faculté de Médecine, Université Paris-Saclay, Villejuif, France; 108 Clinical Gerontology Unit, University of Cambridge, Cambridge, UK; 109 Division of Epidemiology, Department of Internal Medicine, University of Utah School of Medicine, Salt Lake City, UT USA; 110 George E. Wahlen Department of Veterans Affairs Medical Center, Salt Lake City, UT USA; 111 Department of Laboratory Medicine and Pathology, Mayo Clinic, Rochester, MN USA; 112 Nuffield Department of Population Health, University of Oxford, Oxford, UK; 113 Program in Genetic Epidemiology and Statistical Genetics, Department of Epidemiology, Harvard School of Public Health, Boston, MA USA; 114 Division of Epidemiology, Department of Medicine, Vanderbilt University Medical Center, Nashville, TN USA; 115 International Epidemiology Institute, Rockville, MD USA

**Keywords:** Prostate cancer, Prostate cancer

## Abstract

Tumour-promoting inflammation and immune evasion are key features of cancer, but prospective evidence linking specific immune pathways to prostate cancer risk remains limited. Here we show that circulating proteins involved in immune regulation may influence prostate cancer development. We analyse 368 immune- and inflammation-related proteins measured in blood samples from men in the European Prospective Investigation into Cancer and Nutrition (EPIC), including 1434 cases and matched controls, and combine these results with data from UK Biobank. While no proteins are associated with risk in EPIC alone after multiple testing correction, meta-analysis identifies several proteins linked to prostate cancer risk, including IL15 and FLT3LG, in particular, in blood taken long before diagnosis. Higher levels of immune surveillance-related proteins, particularly those involved in natural killer and T cell activity, are associated with lower prostate cancer risk, whereas genetic evidence implicates PARP1, SPINT2 and NME3 in increased risk. These findings suggest that immune pathways play a role in prostate cancer development and highlight potential targets for prevention.

## Abstract (web summary)

Role of specific immune and inflammation-related pathways and protein markers in the development of prostate cancer remains to be elucidated. Here the authors show evidence for potential protective role of dendritic cell mediated immunosurveillance, with protective associations with immune and inflammation related proteins measured more than seven years before diagnosis.

## Introduction

The role of inflammation and the immune system in cancer aetiology is complex. While chronic inflammation is a hallmark of cancer and can promote cellular proliferation, immune and inflammation-related signalling can also detect and eliminate abnormal cells, preventing tumorigenesis via immunosurveillance^1–3^. To date, for prostate cancer (PC), there is suggestive epidemiological evidence for an association with a limited number of relevant candidate proteins, such as CRP and IL6^4^. A systematic review and meta-analysis of inflammatory markers and cancer risk, which included up to seventeen studies of prostate cancer, found CRP to be the most frequently examined marker; although most studies reported null findings, pooled prospective estimates revealed a weak but statistically significant association between elevated CRP and PC risk. IL6 showed no consistent effect, with one study reporting a negative association and three studies reporting null findings^4^. More recently, a prospective analysis from the Osteoporotic Fractures in Men (MrOS) cohort reported that moderate or high IL10 levels were associated with substantially lower PC risk, with no significant associations for IL6, CRP, TNF-α, or overall inflammatory burden^5^. Despite these insights, prospective observational data on most immune and inflammation-related proteins in relation to PC remain scarce^6^.

Here, we show that circulating immune and inflammation-related proteins are associated with the risk of developing prostate cancer. Using prospective data from the EPIC cohort and replication in the UK Biobank, we identify several proteins linked to prostate cancer risk, including IL15 and FLT3LG, which are associated with lower risk. We further demonstrate, using genetic analyses including exome scores and Mendelian randomisation, that elevated PARP1, SPINT2 and NME3 are implicated in increased risk. Together, these findings suggest that enhanced immunosurveillance, particularly involving natural killer and T cell activity, may play a protective role in prostate cancer development and highlight potential targets for prevention.

## Results

**Fig. 1:**
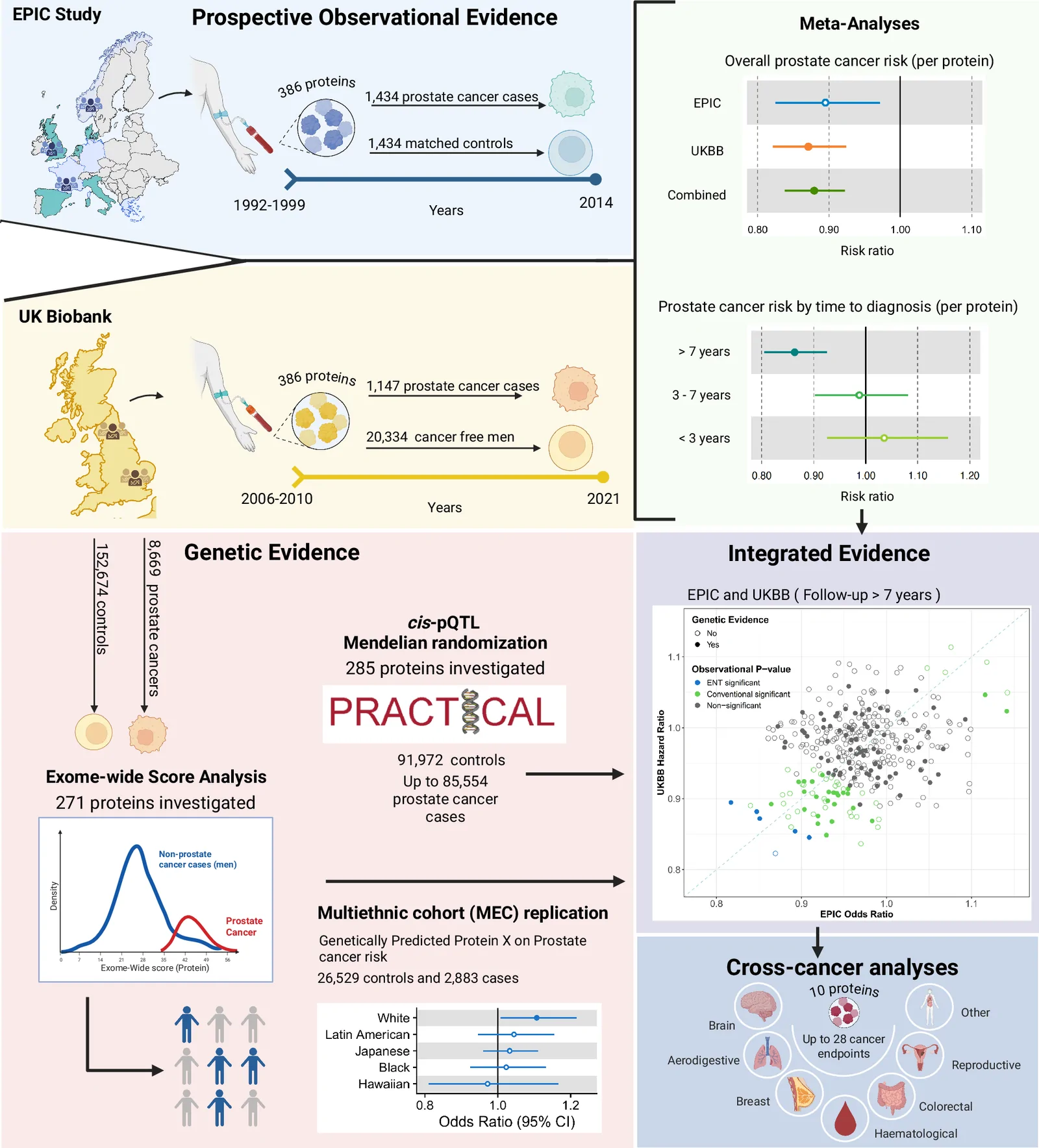
Summary of observational study design and genetic analysis. The observational analyses were based on data from two prospective cohorts, the European Prospective Investigation into Cancer and Nutrition (EPIC) and the UK Biobank (UKBB). Subgroup analyses were conducted by lag time and cancer subtypes. A meta-analysis combining data from both EPIC and UKBB (overall and by lag time) was conducted to strengthen the findings. To further triangulate our results, we used evidence from exome sequencing in the UKBB and Multiethnic cohort, and Mendelian Randomisation (MR) and colocalisation analyses using data from the Prostate Cancer Association Group to Investigate Associated Alterations in the Genome (PRACTICAL) consortium. This figure is created in BioRender. Desai, T. (2026) https://BioRender.com/24opgxy.

A graphical summary of the study design is presented in Fig. 1, encompassing prospective observational analyses in EPIC and UK Biobank, meta-analyses of observational results, genetic evidence from Mendelian randomisation and exome-wide score analyses, and integrated and cross-cancer analyses.

### Observational analyses

In EPIC, median age at PC diagnosis was 67 years [Interquartile range (IQR): 63–72], and the median time from blood collection to PC diagnosis was 12.2 years [IQR: 4.5-14.5]. Small differences were observed in body mass index (BMI) and smoking status between men who were diagnosed with PC and their matched controls. No other differences were observed in participant characteristics by case status (Table 1).

**Table 1:** Baseline characteristics of cases and controls in the EPIC study

Characteristic	Case, N = 1434	Control, N = 1434
**Age at blood collection (year), Mean (SD)**	57.6 (7.5)	57.6 (7.5)
**Weight (kg), Mean (SD)**	79.3 (10.6)	80.3 (11.4)
**Height (cm), Mean (SD)**	172.0 (7.0)	171.9 (7.1)
**Body Mass Index (kg/m^2), Mean (SD)**	26.9 (3.3)	27.2 (3.6)
**Alcohol (g/day), Mean (SD)**	22.1 (24.2)	21.9 (23.6)
**Smoking Status, n (%)**		
Never	486 (33.9)	426 (29.7)
Previous	580 (40.4)	613 (42.7)
Current	331 (23.1)	383 (26.7)
Unknown	37 (2.6)	12 (0.8)
**Educational Attainment, n (%)**		
Primary/None	567 (39.5)	572 (39.9)
Secondary	472 (32.9)	502 (35.0)
Degree	303 (21.1)	288 (20.1)
Unknown	92 (6.4)	72 (5.0)
**Diabetes Status, n (%)**		
Yes	59 (4.1)	63 (4.4)
No	1,347 (93.9)	1,368 (95.4)
Unknown	28 (2.0)	3 (0.2)
**Country, n (%)**		
Germany	276 (19.2)	276 (19.2)
Italy	256 (17.9)	256 (17.9)
Netherlands	92 (6.4)	92 (6.4)
Spain	411 (28.7)	411 (28.7)
UK	399 (27.8)	399 (27.8)
**Time to diagnosis, n (%)**		
<3 years	206 (14.4)	---
3-7 years	311 (21.7)	---
>7 years	917 (63.9)	---
**Stage, n (%)**		
Advanced	273 (19.0)	---
Localised	566 (39.5)	---
Unknown	595 (41.5)	---
**Grade, n (%)**		
High	158 (11.0)	---
Low	1015 (70.8)	---
Unknown	261 (18.2)	---
**Aggressive, n (%)**		
Yes	488 (34.0)	---
No	299 (20.9)	---
Unknown	647 (45.1)	---

In the UKBB study, the median age at PC diagnosis was 69 years [IQR: 65–73]. For men who developed PC, the median duration from blood collection to diagnosis was 6.6 years [IQR: 3.6–9.2]. Differences in various factors were observed between men who were and were not diagnosed with PC during follow-up, including in BMI, Townsend deprivation index, smoking status, and family history of PC (Supplementary Information-Table S2).

### Overall prostate cancer

**Fig. 2:**
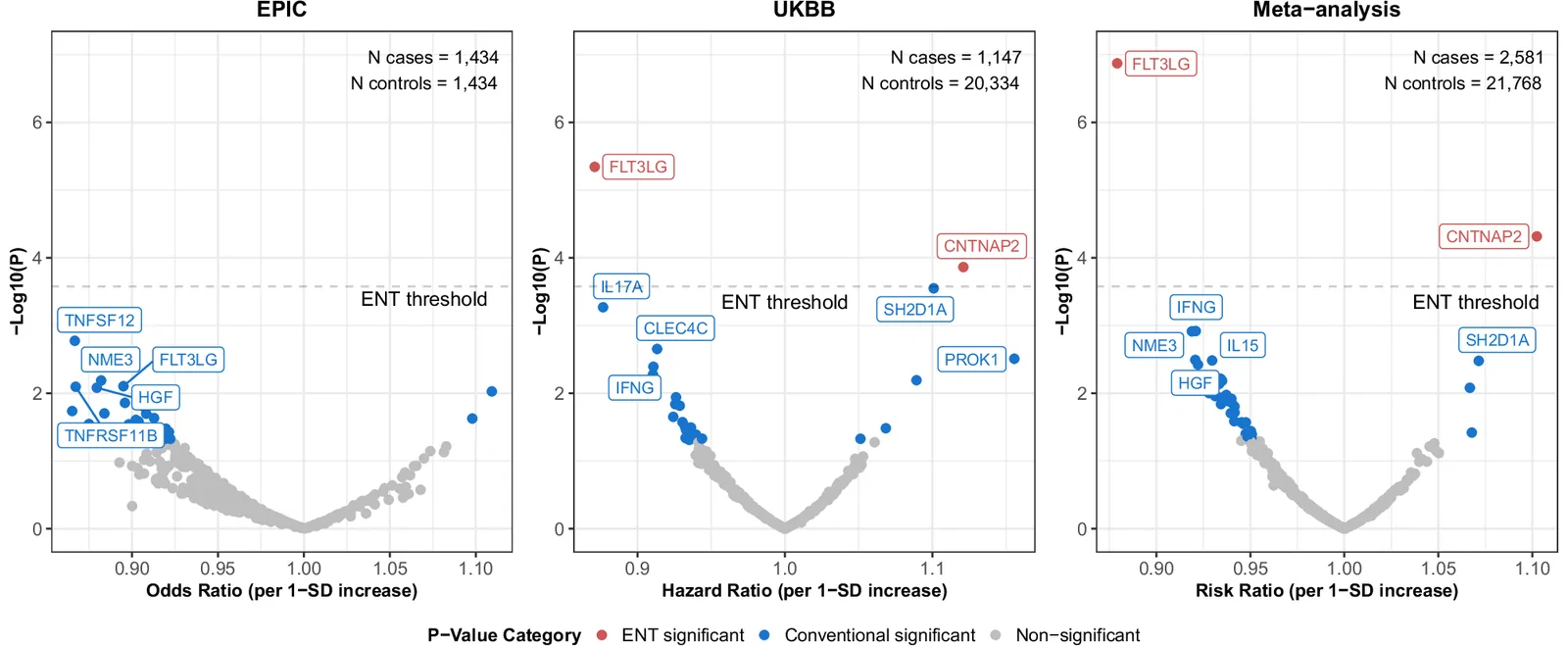
Volcano plots for EPIC, UK Biobank (UKBB), and meta-analysis of overall prostate cancer risk. Associations are reported from fully adjusted models, as described in the Methods section for each cohort. Conditional logistic regression was used for EPIC, and Cox proportional hazards models were used for UK Biobank. The *p*-values shown in the meta-analysis panel were obtained using inverse-variance weighted meta-analysis. All statistical tests were two-sided. The horizontal grey line represents the significance threshold based on the effective number of independent tests (ENT), shown on the −log10 scale; points above this line are considered statistically significant after adjustment for multiple comparisons. The ENT was calculated as 189 effective tests, as described in the Methods. Data corresponding to this figure are provided in Source Data-Figs. 2, 3, and S1.

Figure 2 presents volcano plots of the associations of all 368 proteins with overall prostate cancer risk in the EPIC and UK Biobank cohorts and their meta-analysis. In EPIC, none of the 368 proteins included were associated with the risk of PC overall after ENT correction for multiple testing. In the UKBB, two proteins, FLT3LG and CNTNAP2, were associated with PC risk after correction for multiple testing (HR:0.87, 95%CI:0.82–0.92 and 1.12, 1.06–1.19, respectively), and these proteins were also associated with PC risk in a meta-analysis of results from EPIC and the UKBB (RR_Meta_:0.88, 95%CI:0.84–0.92 and 1.10, 1.05–1.16, respectively) (Supplementary Dataset 1). Evidence of PH assumption violations was identified for 12 proteins using Schoenfeld residuals. For these proteins, extended Cox analyses revealed a significant association of higher IL17A with lower prostate cancer risk in the UKBB; no such association was observed in EPIC. For IL15 and DNER, p-values improved but did not meet the ENT threshold, and results for the remaining nine proteins were materially unchanged.

### Analyses by time to diagnosis

In analyses according to time from blood collection to diagnosis, one protein, CNTNAP2, was associated with an increased risk of PC diagnosed within 3 years of blood collection in the EPIC + UKBB meta-analysis (RR_Meta_:1.25, 95%CI:1.12–1.39) (Supplementary Dataset 1).

**Fig. 3:**
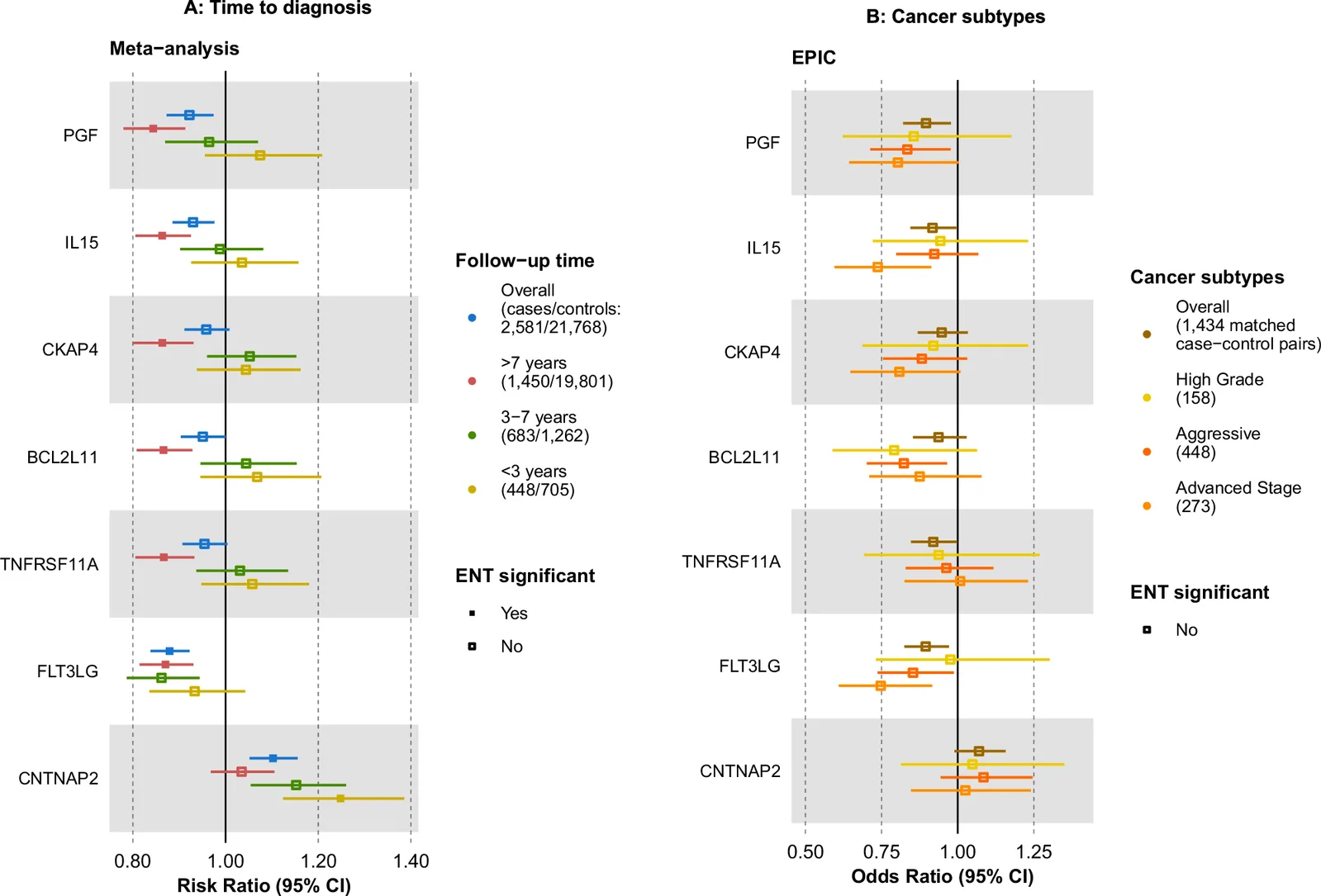
Sub-group analysis of ENT significant association of proteins and prostate cancer risk by time to diagnosis in the meta-analysis of EPIC* +* UKBB (3A) and by cancer sub-types in EPIC (3B). Associations are reported based on the fully adjusted model as defined in the Methods section. Both the risk ratios and odds ratios, with 95% confidence intervals (CIs), represent the effect of a 1-SD increase in protein levels on prostate cancer risk. Multiple testing correction was applied using the Effective Number of Tests (ENT) approach, as described in the Methods section. As data on cancer subtypes are not yet available from UKBB, the results for cancer subtypes are based on the EPIC study. Data for this figure are provided in Source Data-Figs. 2, 3, and S1.

For PC diagnosed more than seven years after blood collection, in the EPIC study, higher IL15 was associated with a lower risk of PC (OR_EPIC_:0.82, 95%CI:0.73–0.91). In the long lag-time analyses in the UKBB, higher levels of FLT3LG and BCL2L11 were associated with lower risks of PC (RR_Meta_:0.85, 95%CI:0.78–0.92 and 0.85, 0.79–0.93, respectively) (Supplementary Information-Fig. S1). In the long lag-time meta-analyses, six proteins were significantly associated with lower overall PC risk: IL15 (0.86, 0.81–0.93), FLT3LG (0.87, 0.81–0.93), BCL2L11 (0.87, 0.81–0.93), PGF (0.84, 0.78–0.91), CKAP4 (0.86, 0.80–0.93), TNFRSF11A (0.87, 0.81–0.93) (Supplementary Dataset 1) (Fig. 3).

### Analyses by prostate cancer subtypes

In EPIC, no proteins were associated with high-grade, advanced-stage, or aggressive PC after accounting for multiple testing. However, we identified inverse associations at conventional significance for IL15 (0.74, 0.60–0.91) and FLT3LG (0.75, 0.61–0.92) with advanced stage and BCL2L11 (0.82, 0.70–0.97), PGF (0.83, 0.71–0.98), and FLT3LG (0.85, 0.74–0.99) with aggressive PC subtypes (Fig. 3). A sensitivity analysis showed that effect estimates for the identified proteins were consistent between the minimally adjusted and fully adjusted models, indicating that the observed associations were not materially explained by the additional covariates included in the fully adjusted model (Supplementary Information–Fig. S5). The full results of protein–prostate cancer associations for both the minimally and fully adjusted models, including overall analyses, lag-time analyses, and cancer subtypes, are presented in Supplementary Dataset 2.

### Correlations between and mutual adjustment for selected proteins

**Fig. 4:**
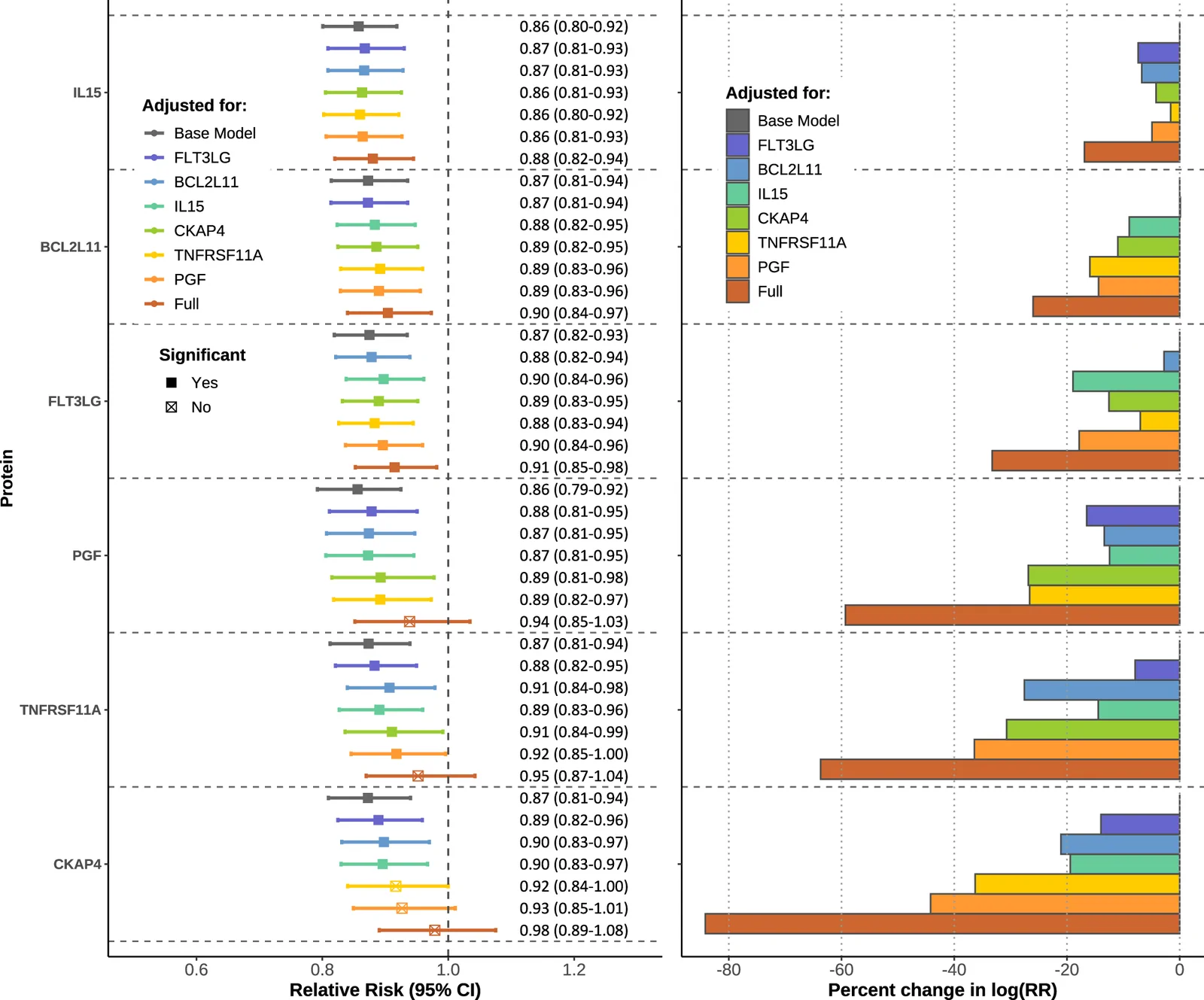
Relative risk (95% CI) for identified risk proteins in relation to prostate cancer diagnosed more than seven years from recruitment after pairwise and mutual adjustment, and percent change in the magnitude of the associations. Percent changes were calculated by comparing the magnitude of association of each protein with prostate cancer risk before and after pairwise or mutual adjustment among the six inflammatory proteins that were statistically significant in the long lag time meta-analysis of EPIC and UK Biobank (follow-up >7 years; FLT3LG, IL15, PGF, TNFRSF11A, BCL2L11, and CKAP4). The mutual and pairwise adjustment analyses were conducted using EPIC long lag time data, comprising 886 matched case-control pairs with follow-up greater than seven years and complete data for all six proteins. All models were adjusted for smoking, alcohol intake, BMI, education, and diabetes status. The base model includes each protein individually with the above covariates; pairwise and mutual adjustment models additionally include one or all remaining proteins, respectively. Data underlying this figure are provided in Source Data-Fig. 4.

We identified modest correlations between proteins identified in observational analyses (IL15, FLT3LG, BCL2L11, PGF, CKAP4, TNFRSF11A, and CNTNAP2) (Supplementary Information-Figs. S2, 3). Multivariable analyses to assess the impact of each of these proteins separately, and together showed that, after mutual adjustment, IL15, BCL2L11, and FLT3LG were significantly associated with overall cancer risk (Fig. 4, Supplementary Information-Fig. S4). Using multivariate network analysis, we also note that IL15 had a greater number of connections to other immune and inflammation-related proteins and was more central in cases with greater than 7 years of follow-up than in their matched controls (Supplementary Information, Figs. S6–8). This is in line with our observation that IL15 was least affected by mutual adjustment for other risk proteins identified in observational analyses.

### Exome analyses

Baseline characteristics of UK Biobank participants included in the exome analysis are presented in Supplementary Information-Table S3. Of the 368 proteins, 347 had available exonic genetic instruments reported by Dhindsa et al within the UKBB, and for these 347 proteins, we conducted an analysis of genetically predicted protein concentrations with PC risk.

Out of the 368 proteins, we were able to construct cis-pQTL exGS for 262 proteins with a median of 2 independent weighting variants per protein. Scores for each male participant were generated within the UKBB, and PC risk was assessed. This identified 16 proteins that were associated with overall PC risk at nominal significance; after correction for multiple testing, one protein, CEACAM21, was associated with risk (OR: 0.95, 95%CI: 0.93–0.98). Full cis-pQTL results of the protein–cancer associations are provided in Supplementary Dataset 3.

For all 347 proteins, we also derived exGS that combined *cis*- and *trans*-pQTLs with a median of 16 independent weights per protein. We identified 266 proteins that were associated with overall PC risk at nominal significance; after correction for multiple testing (0.05/347), we identified two proteins, PARP1 and CEACAM21, associated with a risk of PC overall (OR: 1.05, 95%CI:1.02–1.07, OR:0.96, 95%CI:0.93–0.98, respectively). Full exome results of the protein–prostate cancer associations are provided in Supplementary Dataset 4.

**Fig. 5:**
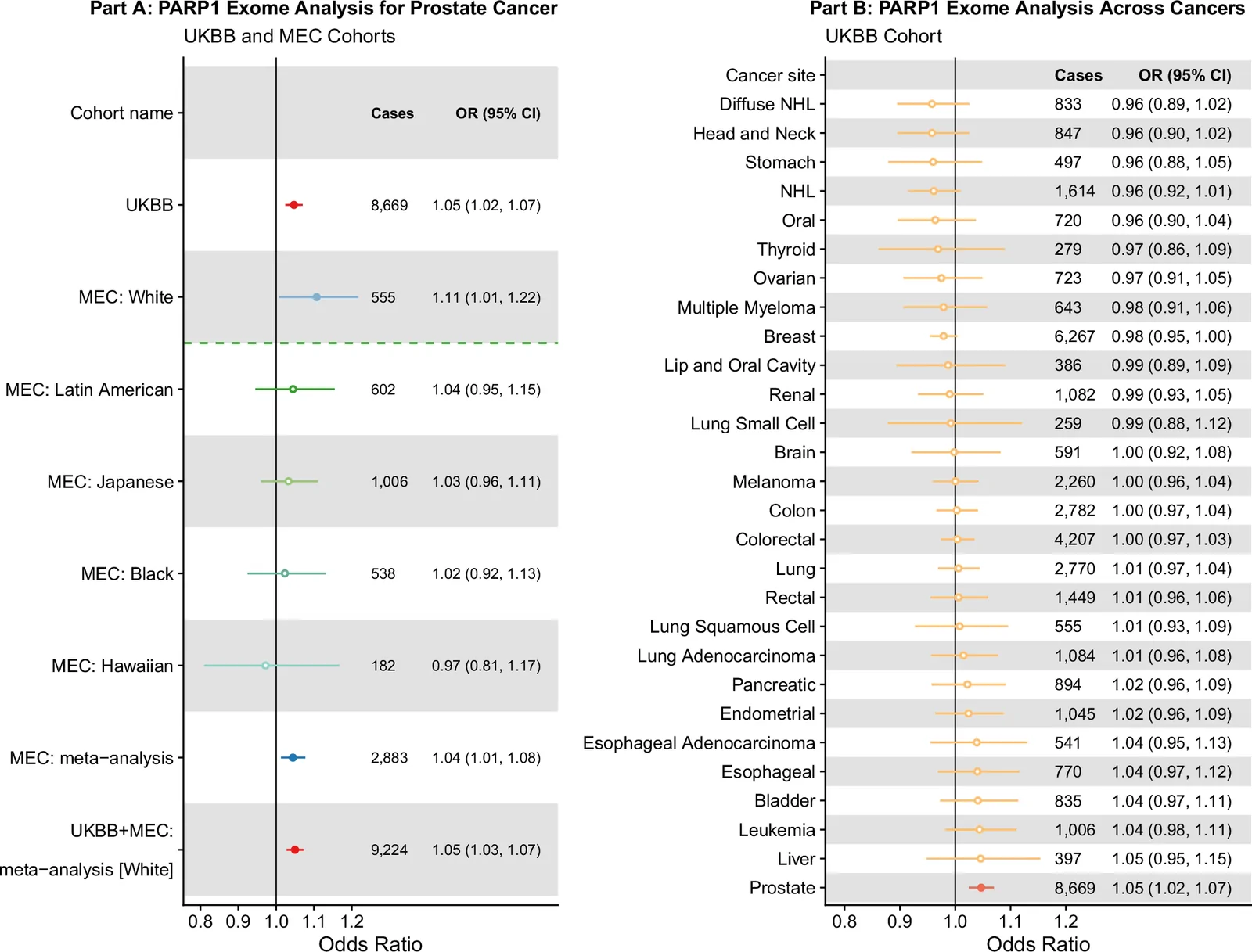
Exome analysis of PARP1 in relation to risk for prostate cancer and other cancers. Part A shows a forest plot of odds ratios (95% CIs) from the exome-wide genetic score (exGS) analysis of PARP1 using a combined panel of independent cis- and trans-pQTLs, with variants oriented to the protein-increasing allele and weighted by beta estimates from Dhindsa et al. Logistic regression models were used to estimate the association of a one standard deviation (SD) increase in genetically predicted PARP1 levels with prostate cancer risk, adjusted for age and the first ten genetic principal components of ancestry. Results are shown for the UK Biobank (UKBB; European ancestry MEN) and for ethnicity-specific analyses in the Multiethnic Cohort (MEC; African American, European American, Japanese American, Latino, and Native Hawaiian men). Meta-analyses were performed using an inverse-variance weighted approach, combining results across ancestry groups within the MEC, and separately combining UKBB with the European-ancestry subset of the MEC. Part B displays a forest plot of odds ratios (95% CIs) for PARP1 across multiple cancer types based on the same exGS approach conducted in the UKBB cohort. Bonferroni correction was applied to account for multiple testing across the number of proteins investigated. Data for this figure are provided in Source Data-Fig. 5.

In the Multiethnic Cohort (MEC), a combined multiethnic analysis found that genetically predicted PARP1 levels were also associated with an increased risk of PC (OR:1.04, 95%CI:1.01–1.08). See Supplementary Information-Table S1 for more information about the SNVs used within the PARP1 panel. In ethnicity-specific analyses, we found directionally concordant associations for higher PARP1 with PC risk among African American, European American, Latino, and Japanese American men but not Native Hawaiian men (Fig. 5).

### Mendelian randomisation and colocalisation analyses

We also conducted MR analyses for PC for the 285 (out of 368) proteins in our study that had available *cis*-pQTLs (see Supplementary Dataset 5 and Supplementary Information-Table S4 for complete MR results and the number of eligible cis-SNPs for each protein, respectively). Sixty-five proteins were associated with overall prostate cancer risk at nominal significance, ten of which remained significant after correction for multiple testing. Three of these ten proteins, CDSN, HLA-DRA, and HSPA1A, were located within the MHC. While MR results for these proteins are reported for completeness, due to the complexity of LD and the sensitivity of colocalisation methods to LD references, we chose not to conduct colocalisation analyses for cis-pQTL MR results located in the MHC region.

Colocalisation analyses showed support for a shared causal variant for SPINT2 (OR:2.11, 95%CI:1.70–2.63, PP4: 97%) and NME3 (1.25, 1.11–1.41, PP4: 71%) with PC and these conclusions were generally robust to the choice of priors (i.e. SPINT2 colocalised when p12 = 1e-4 to 1e-6, while NME3 colocalised when p12 = 1e-4 to 1e-5). No support from colocalisation was found for the five remaining MR findings (CEACAM21, MGLL, GAL, DAG1, SPRY2), which may instead imply these associations are explained by confounding due to LD (Supplementary Dataset 6). No consistent evidence across our included MR methods for associations for risk of prostate cancer was observed with protein levels in reverse MR analyses, suggesting that our observed protein-PC associations were not likely due to reverse causation (Supplementary Dataset 7).

### Integrating observational and genetic evidence

Concordant with observations from our observational analyses from meta-analyses in EPIC and UKBB, we found evidence at conventional significance in exome protein score analyses for inverse associations of FLT3LG (OR:0.97, 95%CI:0.95–0.99) and PGF (OR:0.96, 95%CI:0.94–0.98) with PC risk (Supplementary Dataset 1). We found evidence for the associations of SPINT2 and NME3, identified using MR and colocalisation, with risk of PC diagnosed greater than 7 years after blood collection using EPIC + UKB meta-analyses. However, prospective evidence was discordant and showed inverse associations with PC risk for SPINT2 (OR:0.88, 95%CI:0.82-0.95) and NME3 (0.91, 0.85–0.98), whereas MR suggested higher levels lead to a higher risk.

### Cross-cancer analyses for identified risk proteins

We evaluated the associations of proteins identified in our prostate cancer risk analyses with the risk of up to 27 other cancers and cancer subtypes, using both observational and genetic approaches. After correcting for multiple testing, we found no strong evidence linking the seven proteins highlighted in our prospective prostate cancer meta-analyses to the risk of other cancers (Supplementary Information-section S4.1, Figs. S9–15). In genetic analyses, exGS-based genetically predicted PARP1 levels showed no association with the risk of other cancers (Fig. 5-Part B). We also did not find supportive evidence for an association of SPINT2 and NME3 with risk of other cancers based on cis-pQTL Mendelian randomisation and colocalisation analyses (Supplementary Information-section S4.2, Figs. S16, 17).

## Discussion

We investigated the associations of up to 368 immune and inflammation-related proteins with PC risk using prospective observational data on a total of 2581 men who developed PC from EPIC and UKBB, as well as using exome protein score data for more than 8500 incident cases from UKBB, and MR analyses in approximately 80,000 cases in PRACTICAL. After meta-analysis of results from EPIC and UKBB, we found that higher FLT3LG and CNTNAP2 were associated with a lower and higher risk, respectively, of overall PC risk, while higher FLT3LG, IL15, PGF, CKAP4, BCL2L11, and TNFRSF11A were associated with a lower risk of PC diagnosed more than seven years after blood collection. Exome score analyses that we externally replicated in a multiethnic population also suggested that men with genetically elevated PARP1 have an increased risk of PC. MR analyses also highlighted a possible role for SPINT2 and NME3 in PC risk. Additionally, our cross-cancer analyses did not find evidence for a role of these immune and inflammation-related proteins in the risk of other cancers, although statistical power was limited to detect associations for some endpoints.

Broadly, our main finding is a potentially protective role for heightened immuno-surveillance or preparedness in the aetiology of PC via one or more of the recruitment and differentiation of T and natural killer (NK) cells, and an increase in T cell stamina and memory. We note that the associations for immunosurveillance-related proteins were generally either restricted to or more prominent in samples taken from men whose PCs were diagnosed more than seven years after blood collection. Our findings are consistent with potential endogenous processes/factors acting early in prostate cancer aetiology rather than the associations reflecting markers of subclinical disease. Prostate cancer often evolves slowly and typically becomes immunologically cold, with low tumour-infiltrating lymphocytes, such as active T cells and NK cells, in tumour tissue compared to tissue from healthy prostates^3,7^. Circulating immune proteins measured well before diagnosis may therefore reflect host immunosurveillance and tissue homeostasis at a time when these processes are intact, rather than reflecting inflammatory responses to an existing lesion.

More specifically, we identified an association of higher plasma IL15 with a lower risk of PC using both blood samples taken more than seven years prior to diagnosis. IL15 is expressed primarily on dendritic cells and is known to enhance the proliferation, survival, and maturation of CD8 + T-cells and NK cells and to facilitate anti-tumour activity^8,9^. After mutual adjustment for our protein associations with PC, associations of IL15, as well as BCL2L11 and FLT3LG, remained. Given the inter-correlations, it is possible that this mutual adjustment may represent statistical over-adjustment, and equally in terms of explanatory biologic pathways, adjusting multiple immune and inflammation-related proteins may represent over-adjustment if the main effect of one protein on risk is via its regulation of another’s levels. Nonetheless, taken at face value, our results suggest that IL15 is the most likely sentinel protein association. Notably, IL15 knockout mice also lack NK cells^9^. Further, in-vitro findings suggest that higher IL15 expression may decrease the migration and invasion of PC cells and increase the infiltration of neutrophils in the tumour tissue^10^. In-vivo studies also showed that IL15 limited the proliferation of PC cells and inhibited tumour growth, either by causing inflammation and modifications in the tumour stroma^10^, or by triggering cell-cycle arrest. Due to these findings, among others, recent work has focused on the use of IL15 in the context of cancer treatment with promising results from early-stage trials^11,12^ and also limited animal models investigating cancer vaccines^13,14^.

We have previously reported an inverse association between FLT3LG and PC risk using both observational and using exome analyses^6^. Here we additionally demonstrate, as with IL15, its strong inverse association with risk in men who developed PC longer after having given blood. FLT3LG is primarily produced by NK cells and controls the development of dendritic cells via binding to FLT3 to increase the presentation of tumour antigens, as part of immunosurveillance that enhances T cell and NK cell activity^15–17^. In addition, FLT3LG is linked to higher levels of stimulatory dendritic cells within the tumour environment^18^, playing a pivotal role in enhancing the effectiveness of immune responses against cancer, including the improvement of outcomes in melanoma patients undergoing anti-PD-1 immunotherapy. Recent reviews further highlight the importance of dendritic cell priming in prostate cancer immunobiology and as a determinant of responsiveness to immunotherapy^19^. Clinical studies also indicate that checkpoint inhibitors have limited overall benefit in unselected prostate cancer but can elicit durable responses in biomarker-defined subsets^20^.

Taken together, therefore, it would not be unreasonable to propose an immune-protective effect in normal or precancerous prostate tissue arising from the IL15-mediated recruitment of dendritic cells via tissue-resident NK cell-derived FLT3LG. In line with this, we note that a dendritic cell-based cancer vaccine, sipuleucel-T, has shown modest efficacy across a range of clinically relevant outcomes, including overall survival from PC, and may have improved efficacy in the presence of higher IL15 activity^21^. This would also be consistent with our observation that TNFRSF11A, which is highly expressed on dendritic cells, is also associated with a lower risk of PC via its normal function to augment specific T cell immunity. However, we cannot rule out the possibility that these are protein markers of other risk factors or underlying processes that reflect pre- or early cancerous changes, such as genomic instability or cellular transition to a cancer state^6^.

Higher BCL2L11, CKAP4, and PGF were also associated with a lower risk of PC diagnosed more than seven years after blood draw. While variants at the BCL2L11 locus have been associated with prostate cancer risk^22^, as with CKAP4 and PGF, a direct biological link to PC is currently unclear. Similarly, further work is needed to understand the associations of SPINT2 and NME3 with PC; while both were associated with an increased risk using MR, results from observational analyses suggested this association may be inverse. One explanation for this may be potential complexities arising from cis pQTL used to proxy protein levels; cis pQTL for SPINT2 were also splicing QTL, with the protein-increasing allele also being associated with high intron-excision ratio, which makes it challenging to ascertain whether MR analyses proxy relative binding affinity for these Olink assays to one over other proteoforms or overall protein abundance.

We also identified genetically predicted higher PARP1 to be associated with an increased risk of PC overall using exome protein score analyses and were successful in replicating this association in an external, diverse prospective cohort consisting of African American, European American, Japanese American, Latino, and Native Hawaiian men. However, we did not find evidence for an association with risk in the observational data, which may be due to limited power to detect a modest association. Inhibition of PARP1, such as with Olaparib used to treat PC patients, is beneficial because it leads to the accumulation of DNA damage and promotes cell death in cancer cells deficient in homologous recombination repair mechanisms, such as those with BRCA1/2 mutations. In the context of PC, Olaparib acts to treat PC by removing this repair mechanism for cancer cells with a high burden of double-strand breaks. Most inherited PC risk from rare variants acts via DNA damage and repair, and therefore, by a similar mechanism, the inhibition of PARP1 may be a valuable avenue to consider for research into the prevention of PC^23,24^. Further investigation, including functional validation, is needed to elucidate its mechanistic role in early-stage disease.

Our study is the largest and most comprehensive analysis of the circulating immune and inflammation-related proteins in relation to PC risk to date. However, we note that the data in this analysis were predominantly from EPIC participants  of European ancestry and that almost all cases in our UK Biobank analyses are also of European ancestry; the promise of these initial proteomics results further underscores the need for the prompt diversification of studies on proteins and cancer and the assessment of the generalisability of findings across populations with differing ancestries.

Our study had several limitations. It was not possible to perform triangulation using genetic epidemiological approaches for all the proteins in our study; for a number of reasons, including limited availability of cis-genetic variation, as was the case for IL15, FLT3LG, and BCL2L11. Although cis-pQTLs typically explain a modest proportion of variance in protein levels, all instruments used in our MR analyses had F-statistics above 10, suggesting adequate instrument strength. Nevertheless, for some proteins, the variance explained was very low, which limited our ability to detect modest effects. Furthermore, most proteins were instrumented by a single independent cis-pQTL, which precluded the application of multi-instrument MR methods designed to account for horizontal pleiotropy, and residual pleiotropy within cis-regions cannot be fully excluded. Limited or no support from MR and colocalisation analyses may also be due to other factors such as case mix in the cancer GWAS and genomic complexity of cis-protein regions, which remain challenging for colocalisation methods to resolve. Discrepancies between results from MR and exome analyses may stem from differences in the genetic variants that contribute to these methods; MR typically leverages largely intronic variants that may determine protein levels by altering the promotion or enhancement of transcription, while exome variants may predict the abundance of differing functional protein sequences in the blood. Further, we investigated a sizable but nonetheless still modest subset of all immune and inflammation-related proteins. Therefore, the seven proteins that we identify in this study likely represent only a fraction of proteins with an important role in PC aetiology. With large-scale high-throughput proteomics increasingly available in population cohorts (e.g., Olink HT and emerging Illumina protein-prep workflows in UK Biobank), it will be possible to broaden coverage beyond the panel we studied. Deeper characterisation of the proteome in larger sample sizes from mature prospective cohorts in the future will be needed to bring greater resolution to the network of the PC risk proteome - in particular, proteins that might be linked to a positive feedback loop for IL15 involving NK cells, DC, and T lymphocytes. Finally, the number of cases was limited for PC subtypes and for some cancers included in our cross-cancer analysis, leading to more limited statistical power to detect associations between protein levels and risk of these specific cancer endpoints.

In conclusion, results from our analyses in two mature prospective cohorts and using MR and exome-based protein scores identify proteomic risk factors for PC and suggest a potential protective role of dendritic cell-mediated immunosurveillance, with evidence for protective associations with immune and inflammation-related proteins measured more than seven years before diagnosis. We also find some multiethnic genetic evidence in two independent cohorts that PARP1 inhibition may have a protective role in PC aetiology in addition to its known role in improving prognosis. The roles of both immunosurveillance and PARP1 inhibition are important topics for further research to guide strategies for PC prevention.

## Methods

### EPIC: study population and case ascertainment

EPIC is a prospective multicentre cohort study that recruited 519,978 participants (153,457 men), who were predominantly white and aged 35–70 years old, from 23 centres in 10 European countries (Denmark, France, Germany, Greece, Italy, the Netherlands, Norway, Spain, Sweden and the United Kingdom)^25^. The summary of the study design is presented in Fig. 1. Information on diet, lifestyle, medical history, and anthropometric measurements was recorded at recruitment between 1992 and 1999. Fasting or non-fasting blood samples were also collected from 387,889 individuals (138,939 men) and are stored at local centres and at the International Agency for Research on Cancer – World Health Organisation (IARC-WHO) in Lyon, France. All participants provided written informed consent, and approval for the research was obtained from the ethical review boards of IARC and from all local institutions where participants were recruited. The current study included male participants who had blood samples from centres located in Germany, Italy, the Netherlands, Spain, and the United Kingdom.

Information on cancer incidence, tumour characteristics, vital status, and cause of death was ascertained through population-based cancer registries in Italy, the Netherlands, Spain, and the United Kingdom, and through active follow-up in Germany via health insurance records, municipality registries, and hospital- or physician-based cancer and pathology registries. PC cases were identified as men diagnosed with first incident PC based on the International Classification of Diseases 10th revision code (ICD-10: C61)^26^ after blood collection and before the end of follow-up. These cases were matched one-to-one with controls who were selected among male cohort participants who were free of cancer (excluding non-melanoma skin cancer) and alive at the time of diagnosis of the index case, using an incidence density sampling protocol. Matching variables were study centre, length of follow-up ( ± 6 months), age at blood collection ( ± 6 months), time at blood collection ( ± 1 h) and fasting duration at blood collection ( < 3 h, 3–6 h or >6 h), and study phase (matched case–control sets were identified after each of two rounds of follow-up and end point data centralisation in EPIC conducted in approximately 2008 and 2014). Samples from each phase were assayed together. The present analyses included 1434 cases and 1434 matched controls.

Information on histological grade and tumour stage at diagnosis was available for 1173 cases (81.8%) and 839 cases (58.5%), respectively. For histological grade, there were 1015 low-intermediate grade (Gleason score <8, or grade coded in the recruitment centre as well, moderately, or poorly differentiated) and 158 high grade (Gleason score ≥8, or grade coded in the recruitment centre as undifferentiated). For tumour stage, 566 cases were clinically localised (tumour-node-metastasis (TNM) staging score of T0-T2 and N0/Nx and M0, or stage coded in the recruitment centre as localised) and 273 cases were clinically advanced (T3-T4 and/or N1-N3 and/or M1, or stage coded in the recruitment centre as metastatic). Death from PC (*n* = 156) was defined as PC being recorded as the underlying cause on the death certificate. We also further classified aggressive PC (*n* = 488) as those with clinically advanced and/or high grade and/or prostate-specific antigen (PSA) > 20 ng/ml at diagnosis based on the definition from the European Association of Urology^27^, and/or those who died from PC.

### UKBB: study population and case ascertainment

UKBB is a prospective cohort that recruited 502,150 adults aged 39–73 years (228,985 men) during 2006–2010 at 22 assessment centres across England, Scotland, and Wales^28^. The baseline assessment included a touchscreen questionnaire (covering socio-demographics, lifestyle factors, and medical history), physical measurements, and biological sample collection. Approval for the UKBB study was obtained from the National Information Governance Board for Health and Social Care and the National Health Service Northwest Multi-centre Research Ethics Committee (06/MRE08/65), and written consent was provided by participants for follow-up via national record linkage. Further details of the UKBB study design have been described elsewhere^29^.

Information on cancer registration and death was obtained through record linkage to national registries (NHS Digital for England and Wales using participants’ NHS numbers, and NHS Central Register for Scotland using the Community Health Index). The follow up time were calculated from the date of recruitment until the earliest date of the following events: withdrawal of consent, emigration from the UK, death, or the study censoring date (December 31, 2020, in England and Wales and November 30, 2021, in Scotland) or date of first incident PC diagnosis (ICD-10: C61) or PC recorded on death certificate if no previous record of a cancer diagnosis was identified. Details for the definition of non-prostate cancer endpoints included in cross-cancer analyses can be found in section S4.1 of the Supplementary Information.

### Proteomic measurements in EPIC and UKBB

We measured 368 immune and inflammation-related proteins for EPIC in 1,434 cases and 1,434 matched controls at the Centre for Human Genetics at the University of Oxford using the Olink Explore Inflammation I panel. For UKBB, these proteins were assayed at the Olink Analyses Service in Sweden for 54,306 participants (44,502 were a random sample) selected as part of the UK Biobank Pharma Proteomics Project (UKBB-PPP), including 1147 prostate cases^30^. In both EPIC and UKBB, circulating proteins were measured in citrated plasma. The Olink Explore platform is based on proximity extension assays (PEA) that are highly sensitive and avoid cross-reactivity with high reproducibility^31^. Relative concentrations of these 368 unique proteins were read out by next-generation sequencing. Measurements are expressed as normalised protein expression (NPX) values, which are log-base-2 transformed. Protein values below the limit of detection (LOD) were replaced with the LOD divided by the square root of 2. Protein values were subsequently mean-centred and standardised.

More than 90% of the proteins had a majority of values above the LOD in both EPIC and UKBB (Supplementary Information-Table S5). Missing values due to technical and measurement issues were low across all proteins in EPIC (median: 2.32%, IQR: 0.65% to 2.32%), and in UKBB (median: 2.97%, IQR: 2.41% to 3.29%) (Supplementary Information-Table S6).

### Exome-sequencing in the UKBB and exonic pQTL discovery

Exome-sequencing data protocols for the UKBB have been described^32^. In brief, exome capture was done using the IDT xGen Exome Research Panel v1.0 that underwent 75 bp paired-end Illumina sequencing on the NovaSeq 6000 platform using the S2 and S4 flow cells. Reads were mapped to the reference genome GRCh38 using BWA-MEM, and variant calling was performed by DeepVariant. We extracted exome variants that were associated with circulating protein concentrations at *p* < 5 × 10^−8^ reported by Dhindsa et al. for 50,829 UKBB participants. The exome pipeline reported by Dhindsa et al. was performed by the AstraZeneca’s Genomics Research (CGR) bioinformatics pipeline^33^. SnpEFF v4.3 was used to annotate single nucleotide variants (SNV) and small insertions and deletions (INDEL) against Ensembl Build 38.92^34^.

### MEC: the multiethnic cohort

The MEC is a prospective cohort study of 215,251 participants (96,810 men) recruited in Hawaii and California between 1993 and 1996^35^. Participants completed a self-administered questionnaire about demographics (e.g., race/ethnicity), diet and lifestyle behaviours (e.g., smoking, physical activity), history of prior medical conditions, and family history of common cancers. The study and the analysis were approved by the institutional review boards at the University of Southern California and the University of Hawaii.

Incident cases of PC were defined as ICD10: C61 and identified by linkage to the Surveillance, Epidemiology, and End Results (SEER) cancer registries covering the states of Hawaii and California. Linkage with these registries is complete through July 31 2015 for Hawaii, and through March 31 2015 for California, with 9328 incident cases of invasive PC identified.

The MEC included 73,139 individuals aged 45–75 years at cohort entry who were genotyped and had biosamples collected. Prevalent cases of PC, breast cancer, or colorectal cancer at baseline (*n* = 7731) were excluded based on self-report or from the SEER registries, resulting in 65,408 individuals (29,520 males) in the MEC Genetics Prospective Cohort. Men not in one of the five main ethnic groups (African American, European American, Native Hawaiian, Japanese American, and Latino) were further excluded (*n* = 108). The final sample for analyses of selected genetically-predicted proteins and PC risk was 29,412 male participants, which included 2883 incident PC cases and 26,529 controls.

### Statistics & reproducibility

As this is an observational study using existing cohort data, no statistical method was used to predetermine sample size. Exclusions were minimised. In EPIC, the primary dataset for this study, one participant was excluded, along with 17 participants with missing covariate data in the fully adjusted model. In the UK Biobank, individuals with a prior cancer diagnosis and those failing proteomics quality control were excluded, leaving 44,635 participants for analysis, including 21,481 men (1147 cases) for this study.

### Observational analyses in EPIC

Pearson’s χ2 tests and paired t-tests for matched case–control sets were conducted for selected anthropometric and lifestyle characteristics. Conditional logistic regression was used to estimate odds ratios (ORs) and 95% confidence intervals (CIs) for the association of each circulating protein concentration with the risk of PC. Minimally adjusted models included adjustment for exact age at blood collection (continuous). Additional sensitivity analyses were conducted for a priori potential confounders of PC diagnosis, such as smoking (never, past, current and unknown), alcohol intake ( < 10, 10–19, 20–39 and ≥40 g of alcohol/day; unknown), body mass index (BMI) (fourths, unknown), attained education level (primary, secondary, degree level and unknown), and diabetes status. These analyses were repeated in subgroups of time from blood collection to diagnosis ( < 3 years [*n* = 206], 3–7 years [*n* = 311], and >7 years [*n* = 917]), age at diagnosis ( ≤ 67 years/> 67 years), and prostate tumour stage (localised/advanced), histological grade (low-intermediate/high), and aggressiveness (aggressive/non-aggressive), for all proteins. We additionally conducted Bayesian network analysis on proteins associated with PC risk, diagnosed at least 7 years after recruitment, with a significance threshold of *p* < 0.01 (see section S3 of the Supplementary Information for more details). This protein-protein network analysis can reveal proteins that are more highly connected in cases compared to controls, providing insights into complex protein interactions for cancer development.

### Observational analyses in UKBB

Cox regression models, using age of diagnosis (or age at last follow-up for controls) as the underlying time variable, were used to estimate hazard ratios (HRs) and 95% confidence intervals (CI) for the association of each protein with PC risk^6^. The proportional hazards (PH) assumption was evaluated using Schoenfeld residuals; where violations were identified, extended Cox models using time-varying effects for proteins were fitted. Analyses were stratified by age at recruitment ( < 45, 45–49, 50–54, 55–59, 60–64, and ≥65 years) and adjusted for geographical region (London, North-West, North-East, Yorkshire and Humber, West Midlands, East Midlands, South-East, South-West, Wales, and Scotland), Townsend deprivation index (fifths), family history of PC (yes, no), and BMI (fourths). For each covariate, missing data were handled by assigning participants to an “unknown” category. Subgroup analysis by time to diagnosis (diagnosed in <3 [*n* = 242] years, 3–7 years [*n* = 372], and >7 years of follow-up [*n* = 533]) was also conducted. Cancer sub-type data are currently unavailable within the UKBB dataset.

### Meta-analysis of protein–prostate cancer associations

We conducted an inverse-variance weighted meta-analysis to combine evidence of the association between inflammatory proteins and PC risk using results from our analyses of data in the EPIC and UKBB cohorts. Since cases and controls are matched at follow-up time in the nested case-control study design, the odds ratio derived from such a study can be interpreted as the hazard ratio, assuming the proportional hazards assumption holds^36^. To avoid confusion, we used the term relative risk (RR) to report the results of the meta-analysis. Heterogeneity among results from these two studies was assessed using the I^2^ statistic. The meta-analysis was done for PC risk overall (total *n* = 2581) and for the association of proteins with PC risk stratified by the time to diagnosis ( < 3 years [*n* = 448], 3-7 years [*n* = 683], and >7 years of follow-up [*n* = 1450]).

### Multiple testing correction in observational studies

To account for multiple testing, we applied the Effective Number of Tests (ENT) approach^37^, which reflects the correlation structure among proteins and provides a less conservative correction than Bonferroni while still addressing the risk of false discoveries. Specifically, we conducted principal component analysis (PCA) on the 368 protein measurements in the EPIC dataset to estimate the number of independent tests; 189 components were required to explain 95% of the variance. Accordingly, a significance threshold of 0.05/189 was applied to all observational analyses in EPIC, UK Biobank, the meta-analysis, and subgroup analyses.

### Exome analyses

#### Cis-pQTL and exome-wide genetic score on cancer outcomes

We investigated protein-cancer associations for 347 of the 368 immune and inflammation-related proteins with exonic variation identified in Dhindsa et al.^33^ using an exome-wide genetic score (exGS) approach using a two-panel design; the first containing only independent (clumping r^2^ < 0.01, 10,000KB) *cis-*pQTLs (n proteins=262), the second - a combined panel of both independent *cis* and *trans*-pQTLs. In all analyses, variants were oriented to the protein-increasing allele, and exGS were calculated by summing the number of independent protein-increasing alleles, weighted by beta estimates from the genotypic model reported in Dhindsa et al. This was then projected in the UKBB exome-sequencing cohort using PLINK2^19^. We subsequently used logistic regression models to estimate the association of a one SD increase in each genetically predicted protein level with PC risk in 161,190 European men within the UKBB. Models were adjusted for age and the first 10 principal genetic components of ancestry. For the exome analysis, conventional significance was defined as p < 0.05, while Bonferroni correction was used as the threshold for multiple test correction across the number of proteins investigated (225) for *cis*-pQTL and combined (314) exGS analyses. The LDpair tool within LDlink^38^ was used to assess the LD between MR instruments (see below) that passed Bonferroni correction and instruments in the exGS panels, with the highest R^2^ reported in Supplementary Dataset 5. Panels constructed in this manuscript can be accessed at GitHub (see Data availability).

Replication analyses were performed for selected combined exGS and risk of incident PC in the MEC. Analyses were conducted using logistic regression models that estimated the association of genetically predicted protein levels with PC risk^39^. These models were conducted separately by ethnicity (African American, European American, Native Hawaiian, Japanese American, and Latino) and adjusted for age of diagnosis (or current age for controls) and the first 10 genetic principal components of ethnicity-specific ancestry. Ethnicity-specific risk estimates were then combined using the inverse-variance weighted approach.

#### Mendelian randomisation and colocalisation analyses

*Cis*-pQTLs meeting genome-wide significance (p-value < 5e-8) and independence at r2 < 0.01 were extracted from the protein GWAS by Sun et al., involving 34,557 UKBB participants of European ancestry, and those with weak instrument strength (i.e., F-statistics<10 [β2/σ2]) were excluded from our study^30^. GWAS summary statistics for overall, early onset, and aggressive PC were obtained from the Prostate Cancer Association Group to Investigate Associated Alterations in the Genome (PRACTICAL) consortium, which included 85,554 overall PC cases and 91,972 controls, 6988 early onset cases and 44,256 controls, and 15,167 aggressive cases and 58,308 controls, all of white European ancestry^40,41^. Early onset PC was defined as cases diagnosed before the age of 55 years, while aggressive disease was defined as having metastatic disease or Gleason score ≥ 8 or PSA > 100 ng/mL or death from PC.

Summary data for *cis*-pQTLs and each PC outcome were harmonised after matching on rsID where available, or after selecting a proxy SNP at r^2^_max_ where r^2^ > 0.8 in 1000 Genomes CEU population with the index *cis*-pQTL where necessary. Harmonised SNPs were oriented to the protein-increasing allele, and 285 proteins were included in cis pQTL MR analyses in this study. Primary risk estimates were odds ratios (ORs) estimated using per *cis*-pQTL Wald Ratios [β-outcome/ β-protein] for a one SD increase in the protein concentration. To account for multiple testing in our MR analyses, a Bonferroni-corrected threshold of 0.05/285 was used based on the number of proteins with available cis-instruments (i.e., 285 proteins). Where MR associations passed correction for multiple testing, we assessed the potential for confounding by LD using Bayesian colocalisation. Within each predefined genetic region (250 kb around the index cis-pQTL), we tested for the presence of a single shared causal variant between protein and cancer using conventional colocalisation, as well as multiple shared causal variants using the Sum of Single Effects (coloc SuSiE) regression method, which can account for the presence of multiple causal variants, of which only a subset are shared between two traits^42^. Default priors were used for analyses, and PP4 > 0.7 was accepted as evidence for colocalisation; we also assessed the sensitivity of our colocalisation results to the choice of priors (with p12 ranging from 1e-4 to 1e-6).

For proteins with significant associations after Bonferroni correction, we conducted reverse MR analyses (with PC modelled as exposure and protein concentrations as outcomes) to examine the possibility of reverse causation. The following MR approaches were used: random-effects inverse-variance weighted, weighted median, MR-Egger, and MR-PRESSO. SNPs associated with each cancer endpoint at *p* < 5 × 10-8 were clumped (r < 0.01, clumping distance=10,000 kb) and summary data for these SNPs (or proxy SNPs if no match is available) were extracted from the protein GWAS. Effect alleles and the corresponding estimates were harmonised between cancer and protein GWAS. We required consistent evidence from multiple methods to indicate the presence of a notable reverse MR association.

All statistical analyses were conducted using R 4.1.1. Cox and conditional logistic regressions were conducted using the survival package; meta-analyses using metafor; MR using TwoSampleMR and colocalisation using coloc (version 5.2.3).

### Reporting summary

Further information on research design is available in the Nature Portfolio Reporting Summary linked to this article.

## Supplementary information

Supplementary Information

Description of Additional Supplementary Files

Supplementary Dataset 1-7

Reporting Summary

Transparent Peer Review file

## Source data

Source Data

## Data Availability

Data from the European Prospective Investigation into Cancer and Nutrition (EPIC) are available upon request and with permission from the International Agency for Research on Cancer (IARC) and the EPIC Steering Committee. Access to UK Biobank data was granted under Application Number 67506. UK Biobank data are available to bona fide researchers for health-related research in the public interest via application at https://www.ukbiobank.ac.uk/register-apply. Raw data for the figures are provided as Source Data. Large tables that cannot be accommodated in the Supplementary Information are provided as Supplementary Datasets 1–7 with this paper. Source data are provided with this paper.

## Code availability

Analysis code is available at https://github.com/ParsaeianM/EPIC-Prostate-Analysis, and the exGS panels used in this study are available at https://github.com/Cancer-epi-unit/INF_PC.
