# Supplementary material for: Prospective and genetic analyses implicate lower immunosurveillance in the aetiology of prostate cancer
Source: Nat Commun. 2026 Nov 10;17:10698. doi: 10.1038/s41467-026-77378-3 (PMC13752312; doi:10.1038/s41467-026-77378-3)
Supplement: Supplementary file 1 [file 41467_2026_77378_MOESM1_ESM.pdf]

S1: Proteomics measurements and quality control

Circulating proteins were measured using the Olink Proteomics multiplex platform (Uppsala, Sweden). The technology is based on a proximity extension assay (PEA) technique that is highly sensitive and avoids cross-reactivity with high reproducibility. The full protocol of the PEA has been reported previously.[1] All sample plates included eight internal control samples to monitor the quality of assay performance, as well as the quality of individual samples. The quality control (QC) was performed in two steps:

1. Protein values per participant where average matched count between PEA measurements and NGS reads was less than 500 counts were removed as per Olink guidelines.
2. Protein values per participant where deviation from the median value of the controls for each participant was greater than 0.3 NPX from the median were excluded.

About 2.1% of all protein measurements were excluded from analyses after QC steps, which led to the exclusion of one individual and their matched pair from the analysis.

For protein measurements, pairs of case-control samples were plated together, with the pairs randomly allocated over 88-well plates. Protein concentrations were measured by next-gen sequencing (qPCR) to quantify relative protein concentrations expressed as normalized protein expression (NPX) values on the log2 scale. Measurements below the lower limit of detection (LOD) were replaced with the Lower limit of detection (LOD) divided by the square-root of 2. A summary of proportion of protein measurements with values below LOD and missing values are presented in Supplementary Information-Table S5 and Table S6, respectively.

S2: Supplementary figures for identified risk proteins in relation to prostate cancer

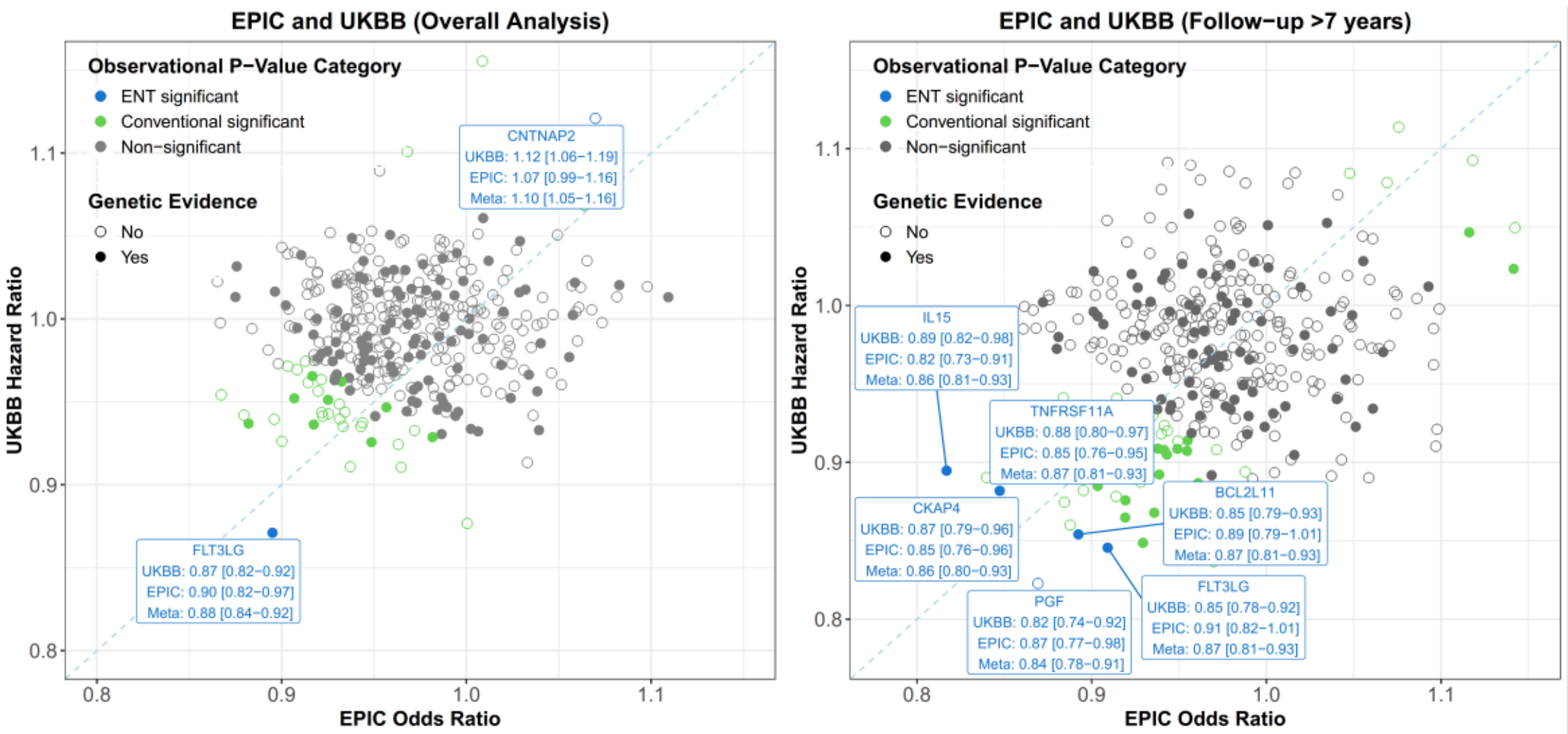

Figure S1: Scatterplot of inflammatory proteins and prostate associations from EPIC, UKBB and meta-analyses

The scatter plot illustrates the consistency of association estimates between the two observational studies (EPIC and UKBB). P-values were derived from conditional logistic regression models using the fully adjusted model, as described in the Methods section. All p-values are two-sided. Labelled proteins are those identified as prostate cancer risk proteins in the overall and long lag time meta-analyses. Point colours represent the strength of evidence from the meta-analysis: blue indicates ENT significance (P < 0.05/189), green indicates conventional significance (P < 0.05), and grey indicates non-significance. Filled circles indicate the presence of supporting genetic evidence from exome or Mendelian randomisation analyses. In the overall meta-analysis, 2,581 cases and 21,768 controls were included; in the long lag time analysis (follow-up >7 years), 1,450 cases and 19,801 controls were included. Data for this figure is provided in Source Data-Figure 2, 3, and S1.

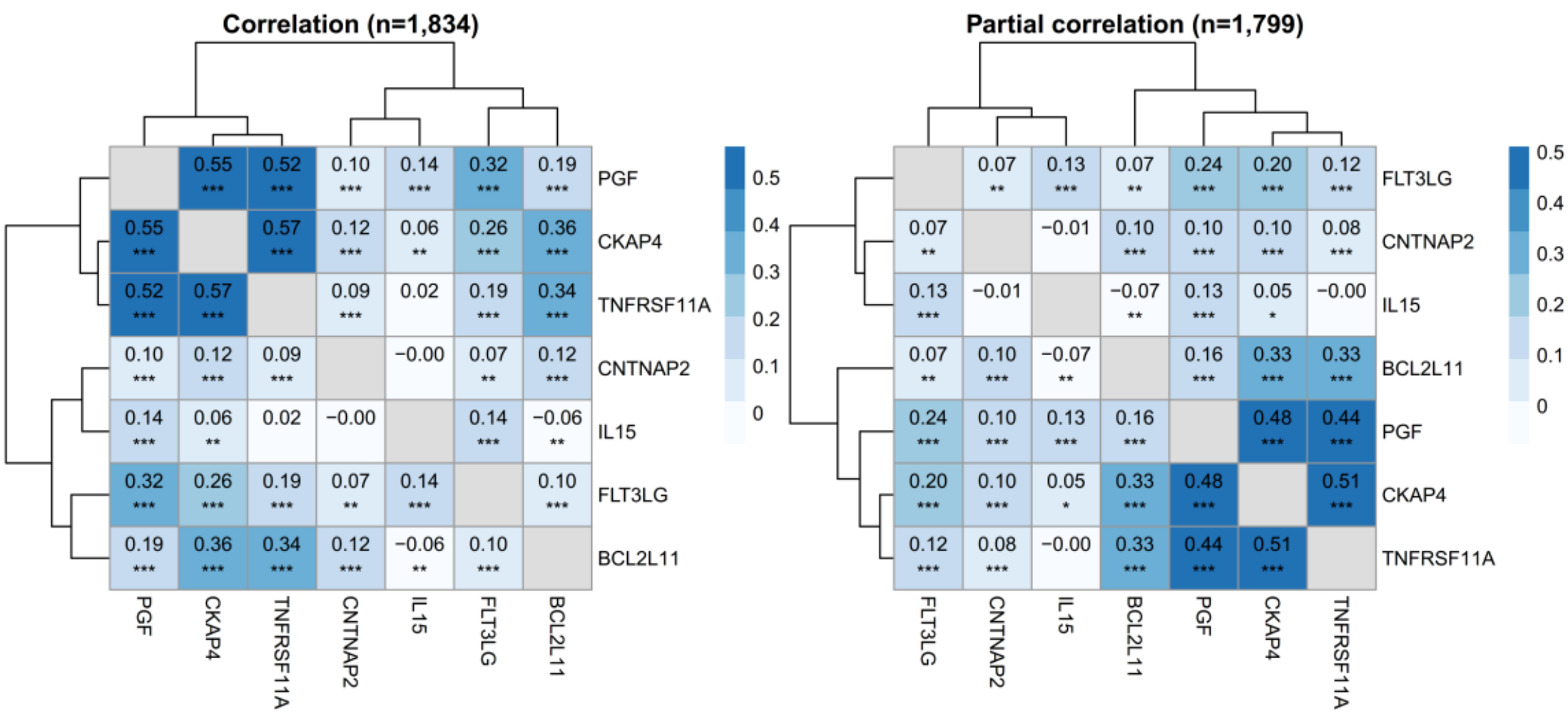

Figure S2: Pearson correlation coefficients among candidate risk proteins related to prostate cancer prostate cancer in EPIC

Correlations are shown for 917 matched case-control pairs diagnosed more than seven years after recruitment in EPIC. Partial correlations were adjusted for age at blood collection and BMI. The sample size is reduced by 35 due to missing BMI values. All p-values are two-sided and were not adjusted for multiple comparisons. Significance levels are indicated as follows: p < 0.05 (*), p < 0.01 (**), and p < 0.001 (***). Data for this figure is provided in Source Data-Figure S2.

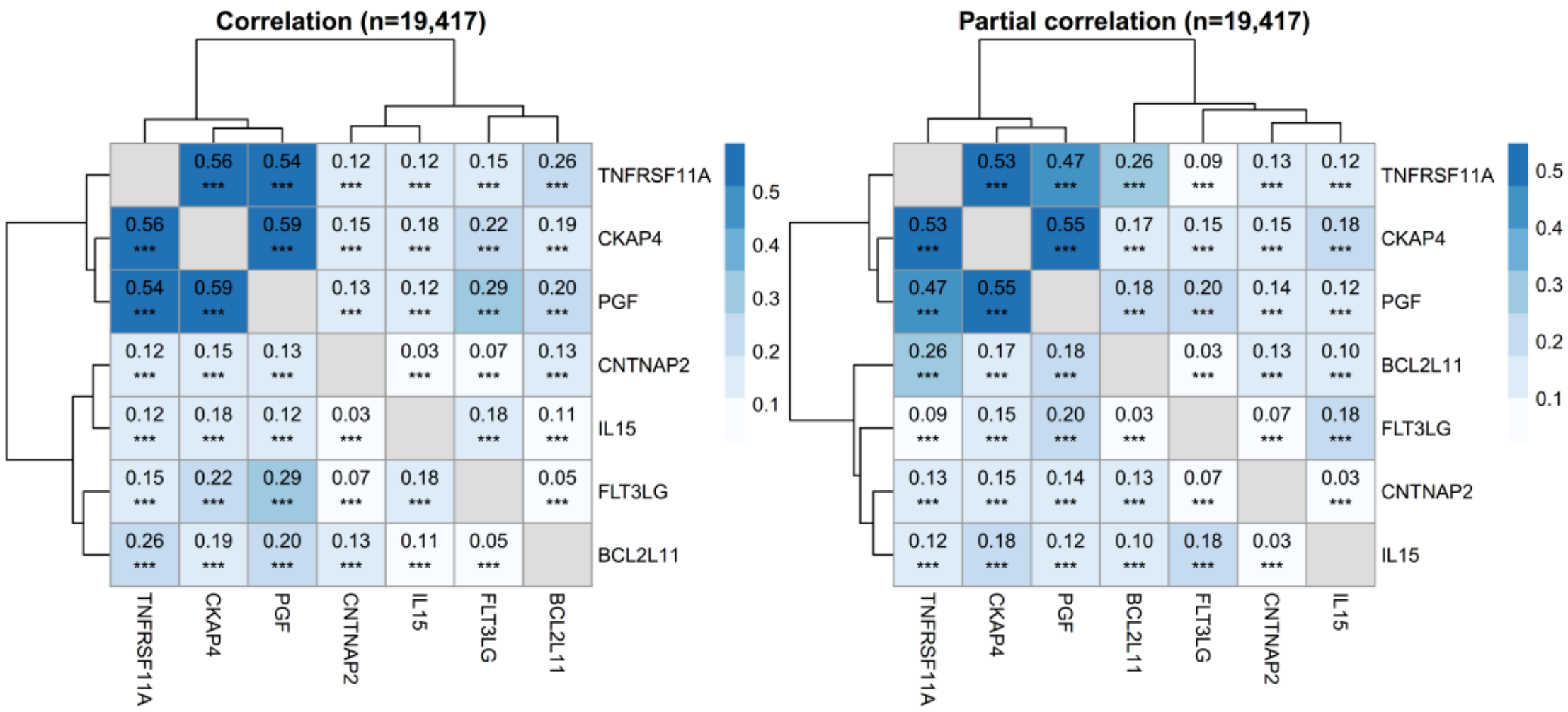

Figure S3: Pearson correlation coefficients among candidate risk proteins related to prostate cancer in the UKBB cohort.

Correlations are shown for 19,417 participants (533 cases and 18,884 controls) diagnosed more than seven years after recruitment in the UK Biobank cohort. Partial correlations were adjusted for age at blood collection and BMI. All p-values are two-sided and were not adjusted for multiple comparisons. Significance levels are indicated as follows: p < 0.05 (*), p < 0.01 (**), and p < 0.001 (***). Data for this figure is provided in Source Data-Figure S3.

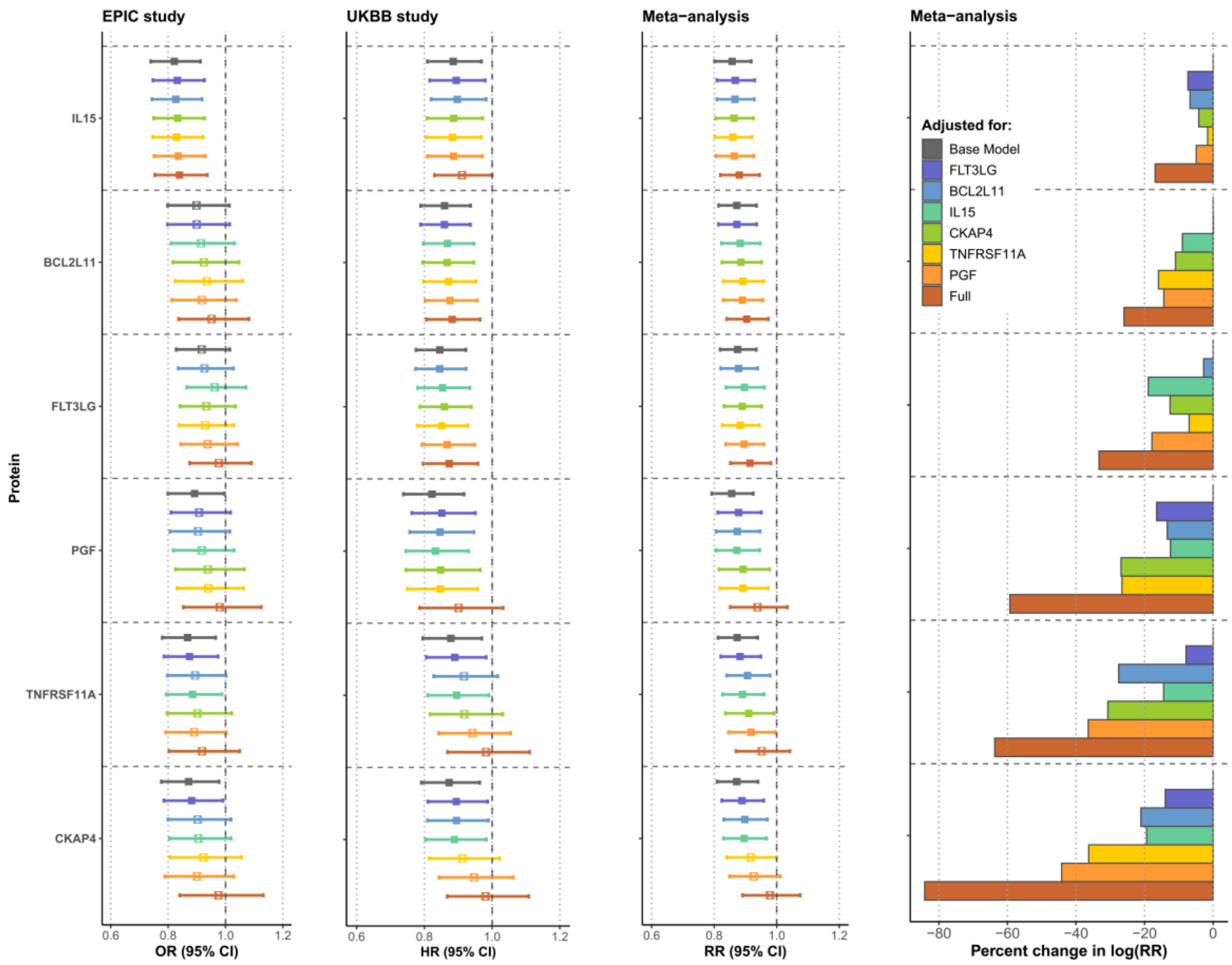

Figure S4: Relative risk (95% CI) for identified risk proteins in relation to prostate cancer diagnosed more than seven years from recruitment after pairwise and mutual adjustment, and percent change in the magnitude of the associations.

Associations between each protein and prostate cancer risk were estimated using conditional logistic regression in EPIC and Cox proportional hazards models in UK Biobank. Percent changes were calculated by comparing associations before and after pairwise or mutual adjustment among the six inflammatory proteins significant in the long lag time meta-analysis (follow-up >7 years: FLT3LG, IL15, PGF, TNFRSF11A, BCL2L11, and CKAP4). These analyses were conducted separately in each cohort among participants with complete data for all six proteins (EPIC: 886 cases; UK Biobank: 508 cases), with all models adjusted for smoking, alcohol intake, BMI, education, and diabetes status. The base model includes each protein individually with these covariates; pairwise and mutual adjustment models additionally include one or all remaining proteins, respectively. Data for this figure is provided in Source Data-Figure S4.

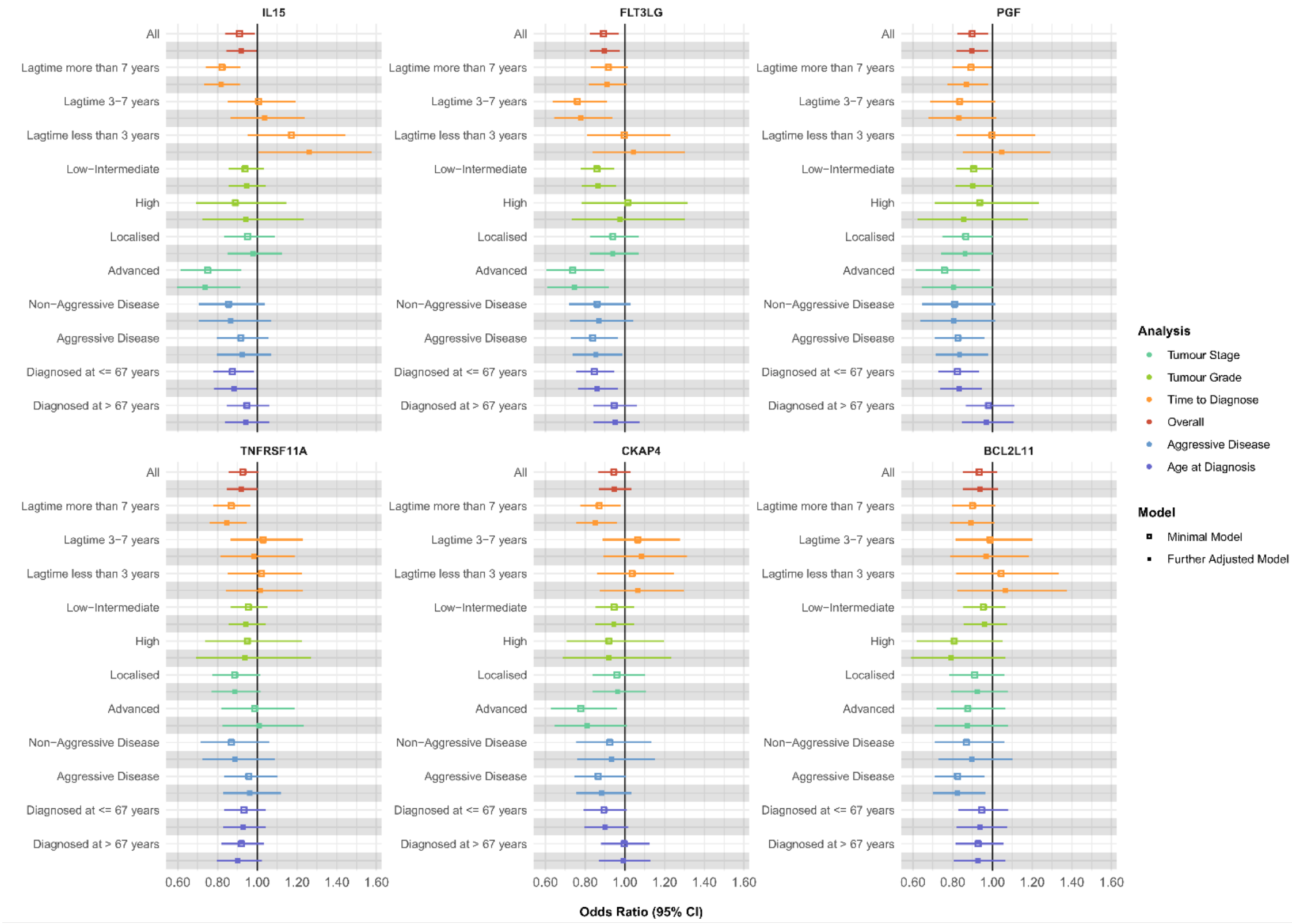

Figure S5: Comparison of minimally and fully adjusted models for the association between selected inflammatory proteins and prostate cancer risk in the EPIC study.

Selected proteins are those that met the effective number of tests (ENT) threshold for multiple-comparison correction based on the meta-analysis results. Odds ratios (ORs) and 95% confidence intervals were estimated using conditional logistic regression models. Confidence intervals are presented at the 95% level.

Minimally Adjusted Model: Adjustment for exact age at blood collection.

Fully Adjusted Model: Additional adjustment for smoking, alcohol intake, attained education level, and diabetes status.

Overall analysis: 1,434 matched case-control pairs. Stratified by follow-up time: <3 years (206 pairs), 3–7 years (311 pairs), and >7 years (917 pairs). Stratified by age at diagnosis: ≤67 years (715 pairs) and >67 years (719 pairs). Stratified by tumor grade: high grade (158 pairs), low-intermediate grade (1,015 pairs), and unknown (261 pairs). Stratified by clinical stage: advanced stage (273 pairs), localised (566 pairs), and unknown (595 pairs). Stratified by disease aggressiveness: aggressive (488 pairs), non-aggressive (299 pairs), and unknown (647 pairs).

Data for this figure is provided in Source Data-Figure S5.

S3: Protein-protein interaction network

Bayesian network analysis was used to explore the inter-relationships among proteins associated with prostate cancer. This approach relies on a probabilistic graphical model, where proteins were represented as nodes and their interdependencies as directed edges.[2] We selected a set of proteins significantly associated with prostate cancer ($p \leq 0.01$) from the EPIC+UK Biobank meta-analysis in sub-group analysis with more than 7 years of follow-up (25 proteins). Using hill-climbing learning method, the algorithm starts with an initial network structure and iteratively modifies the structure (by adding, removing, or reversing edges), aiming to optimize the fit of the model using Bayesian Information Criterion (BIC), which balances model fit and the complexity of the network. The learning process iterated until convergence, in which no modification to the network structure improves the fit of the model. Analyses were conducted using data from cases and their matched controls with more than seven years of follow-up.

To emphasize the most important interactions, edges displaying weaker associations (strength below the 40th quantile) were excluded. Additionally, proteins not directly related to the target proteins (IL15, FLT3LG, CKAP4, PGF, TNFRSF11A, and BCL2L11) were omitted from the network result (only 15 proteins remained in the final analysis).

The protein-protein networks among cases and separately among controls with more than 7 years of follow-up are presented in ***Supplementary Figure S6* & *Supplementary Figure S7***, respectively. To compare the interconnection among cases and controls, we conducted a differential analysis based on nodes' degree of protein-protein interaction network between cases and controls (cases-controls) presented in ***Supplementary Figure S8***.

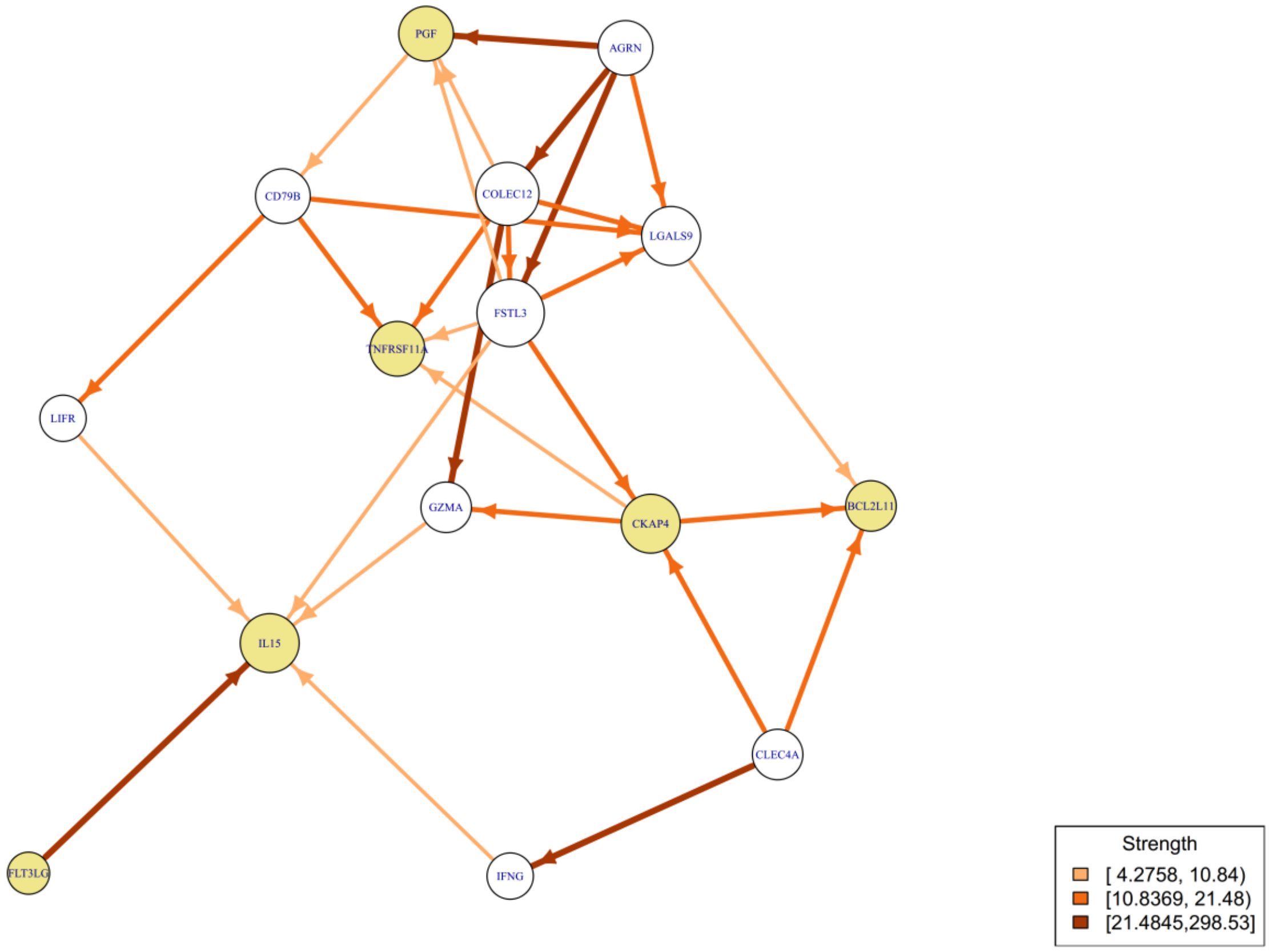

Figure S6: Protein–protein interaction network among EPIC prostate cancer cases diagnosed more than seven years after blood collection.

The network was constructed using Bayesian network analysis among EPIC cases with follow-up greater than seven years (n = 917). Input proteins were those associated with prostate cancer risk ($p < 0.01$) in the EPIC+UK Biobank long-lag meta-analysis. Network structure was learned using a hill-climbing algorithm with Bayesian Information Criterion (BIC) optimization. Edges represent inferred conditional dependencies between proteins; only interactions above the 40th percentile of edge strength were retained. Proteins not directly connected to the six target proteins (IL15, FLT3LG, CKAP4, PGF, TNFRSF11A, and BCL2L11) were excluded from the final network. Nodes highlighted in yellow represent these six prostate cancer risk proteins identified in the long-lag meta-analysis. Data for this figure is provided in Source Data-Figure S6.

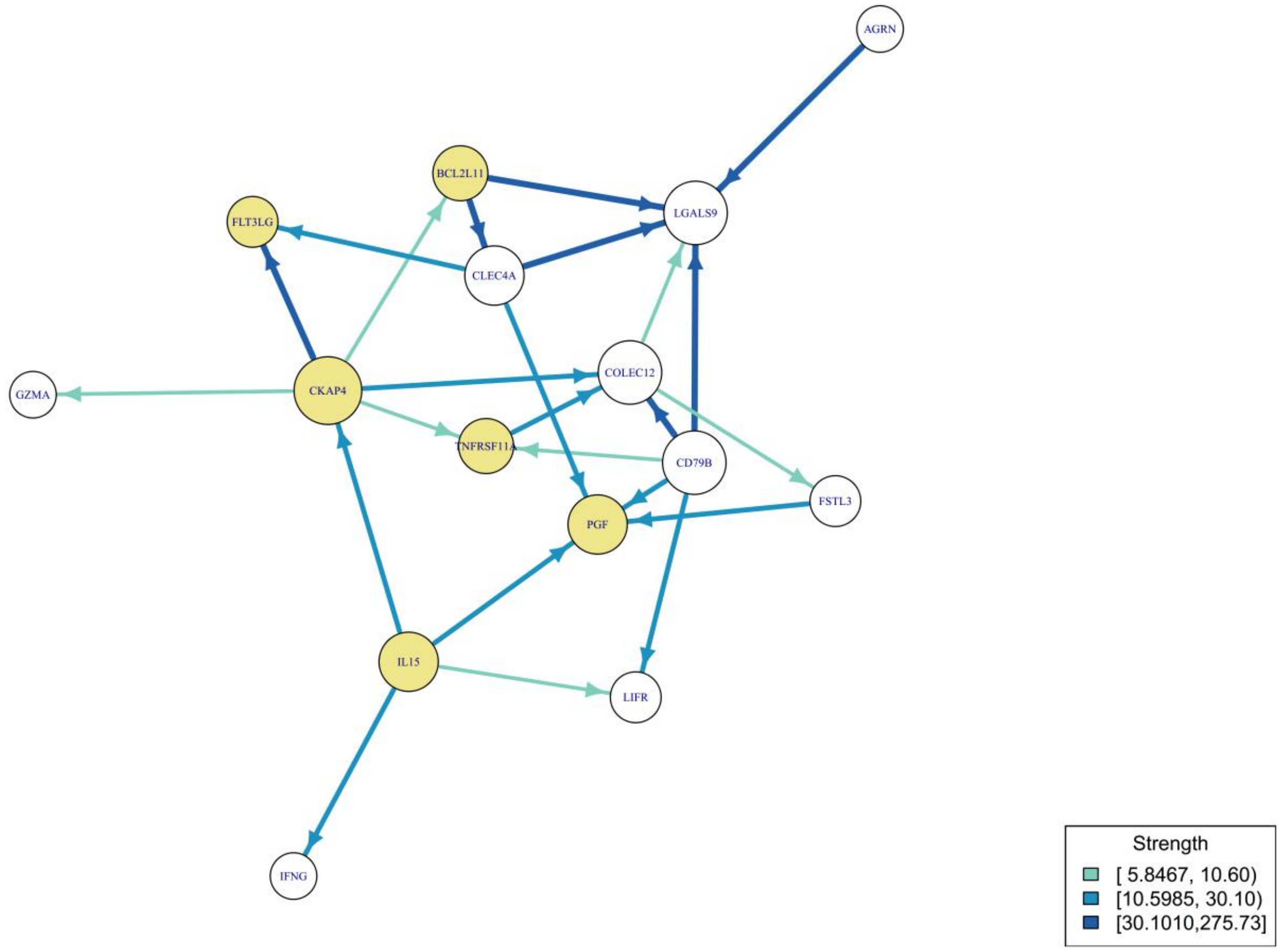

Figure S7: Protein–protein interaction network among EPIC controls matched to prostate cancer cases with more than seven years of follow-up.

The network was constructed using Bayesian network analysis among EPIC controls with follow-up greater than seven years (n = 917). Input proteins were those associated with prostate cancer risk ($p < 0.01$) in the EPIC+UK Biobank long-lag meta-analysis. Network structure was learned using a hill-climbing algorithm with Bayesian Information Criterion (BIC) optimization. Edges represent inferred conditional dependencies between proteins; only interactions above the 40th percentile of edge strength were retained. Proteins not directly connected to the six target proteins (IL15, FLT3LG, CKAP4, PGF, TNFRSF11A, and BCL2L11) were excluded from the final network. Nodes highlighted in yellow represent these six prostate cancer risk proteins identified in the long-lag meta-analysis. Data for this figure is provided in Source Data-Figure S7.

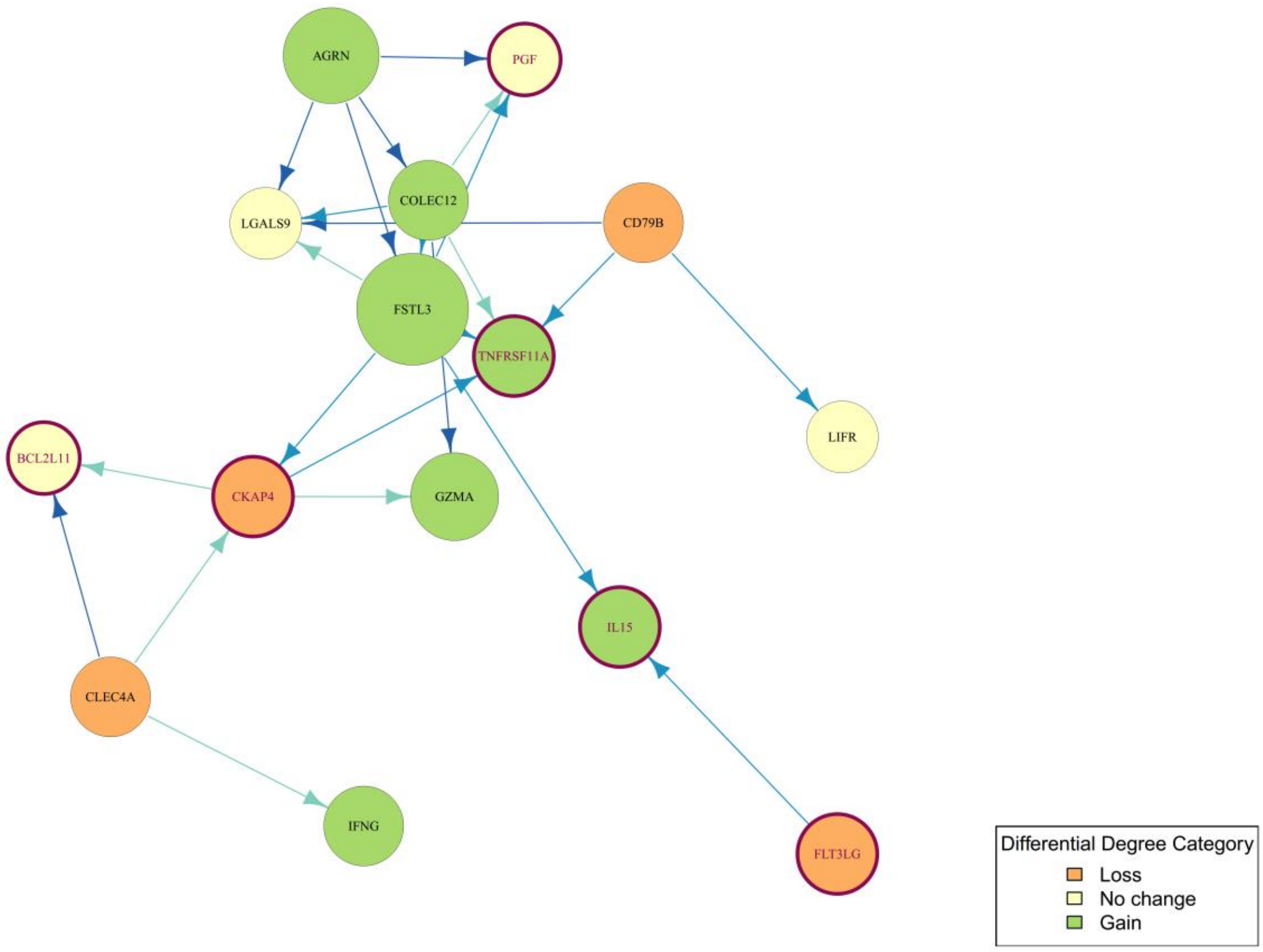

Figure S8: Differential network analysis comparing protein–protein interaction networks between EPIC prostate cancer cases and matched controls with more than seven years of follow-up.

Node degree differences were calculated as the number of network connections in cases minus that in controls, using separate Bayesian network models for each group. Input proteins were those associated with prostate cancer risk ($p < 0.01$) in the EPIC+UK Biobank long-lag meta-analysis. "Gain" indicates higher connectivity in cases than controls, "Loss" indicates lower connectivity, and "No Change" indicates similar connectivity in both groups. Nodes with a red ring represent prostate cancer risk proteins identified in the long-lag meta-analysis. The analysis was conducted in EPIC using 917 matched case-control pairs with follow-up greater than seven years. Data for this figure is provided in Source Data-Figure S8.

S4: Cross cancer analysis of candidate prostate cancer risk proteins

S4.1 Cross cancer analysis in the UK Biobank cohort study

UK Biobank endpoints definition for the observational analyses

For associations with prostate cancer identified in both observational and genetic analyses, we examined the relationship of these proteins with other cancer outcomes using UK Biobank (UKBB) data. Cancer outcomes were defined based on the 10th revision of the World Health Organization's International Statistical Classification of Diseases (ICD-10), including head and neck (C00–C14, C32), oesophagus (C15), stomach (C16), colorectum (C18–C20), liver (C22), pancreas (C25), lung (C34), malignant melanoma (C43), breast in women (C50), uterine (C54), ovary (C56), prostate (C61), kidney (C64–C65), bladder (C67), brain (C71), thyroid (C73), and blood cancers, including non-Hodgkin lymphoma (NHL; C82–C85), multiple myeloma (C90), and leukaemia (C91–C95). The following sub-classifications of these cancer groupings were also considered: oral (C00–14) and lip and oral cavity (C00–06) within head and neck cancers (C00–14, C32); adenocarcinoma of the oesophagus (C15, morphology codes ICD-O-3 8140–8573) within oesophageal cancer (C15); colon (C18) and rectum (including rectosigmoid junction, C19–20) within colorectal cancer (C18–20); adenocarcinoma of the lung (C34, morphology codes ICD-O-3 8140, 8211, 8250–8260, 8310, 8323, 8480–8490 and 8550), squamous cell carcinoma (C34, morphology codes ICD-O-3 8070-8072), small cell carcinoma (C34, morphology codes ICD-O-3 8041-8042) within lung cancer (C34); and diffuse lymphoma (C83) within NHL (C82–85).

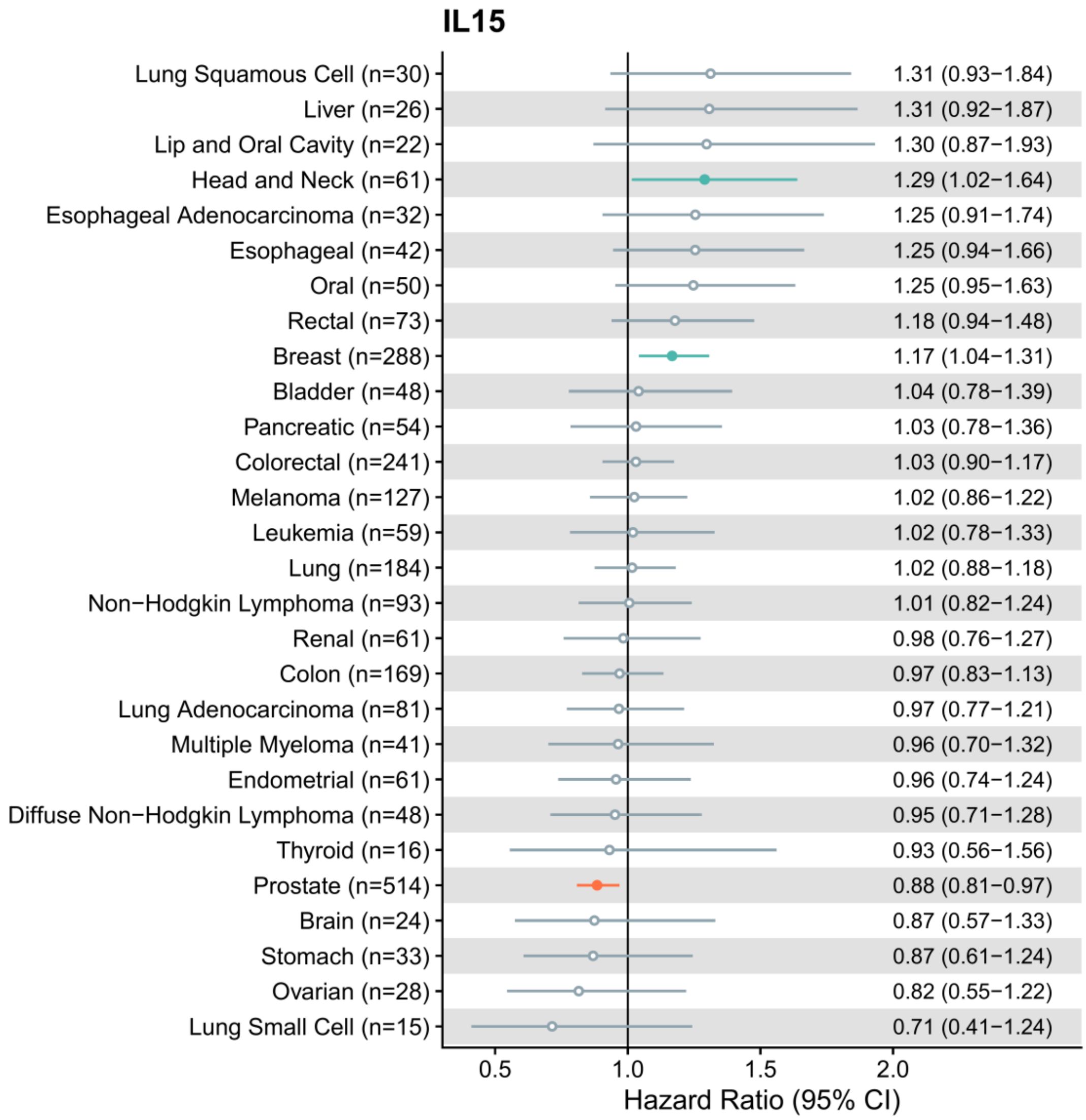

Figure S9: Observational analysis of IL15 in relation to risk for prostate cancer and other cancers diagnosed more than seven years after recruitment in the UK Biobank cohort study. Hazard ratios (HRs) and 95% confidence intervals were estimated using Cox proportional hazards models, using the fully adjusted model as described in the Methods section. Results in red refer to those for risk of prostate cancer, while those in blue indicate an association with $p < 0.05$. Hollow points indicate $p > 0.05$. All p-values are two-sided. No adjustment was made for multiple comparisons. Data for this figure is provided in Source Data-Figure S9-15.

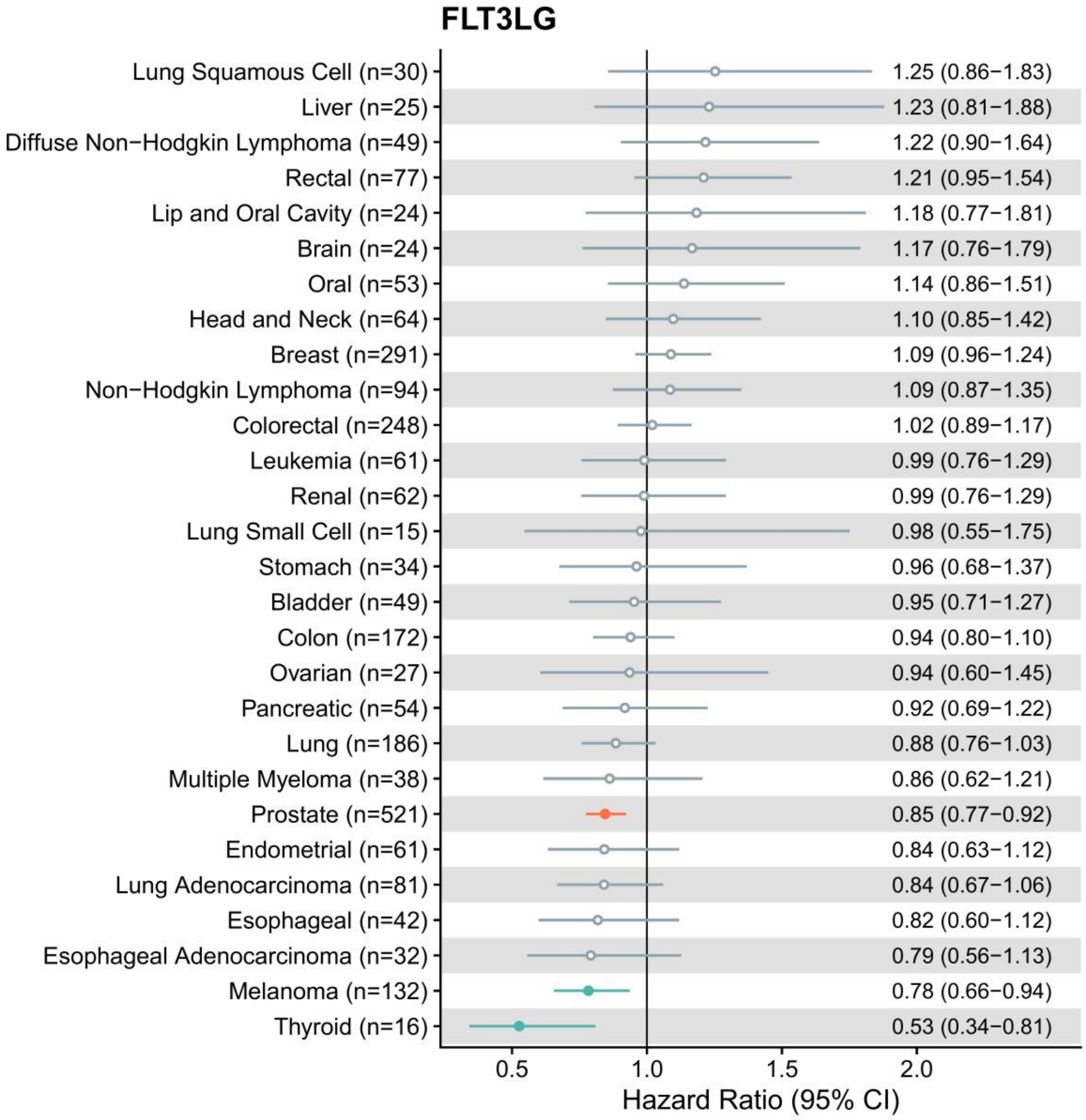

Figure S10: Observational analysis of FLT3LG in relation to risk for prostate cancer and other cancers diagnosed more than seven years after recruitment in the UK Biobank cohort study. Hazard ratios (HRs) and 95% confidence intervals were estimated using Cox proportional hazards models, using the fully adjusted model as described in the Methods section. Results in red refer to those for risk of prostate cancer, while those in blue indicate an association with $p < 0.05$. Hollow points indicate $p > 0.05$. All p-values are two-sided. No adjustment was made for multiple comparisons. Data for this figure is provided in Source Data-Figure S9-15.

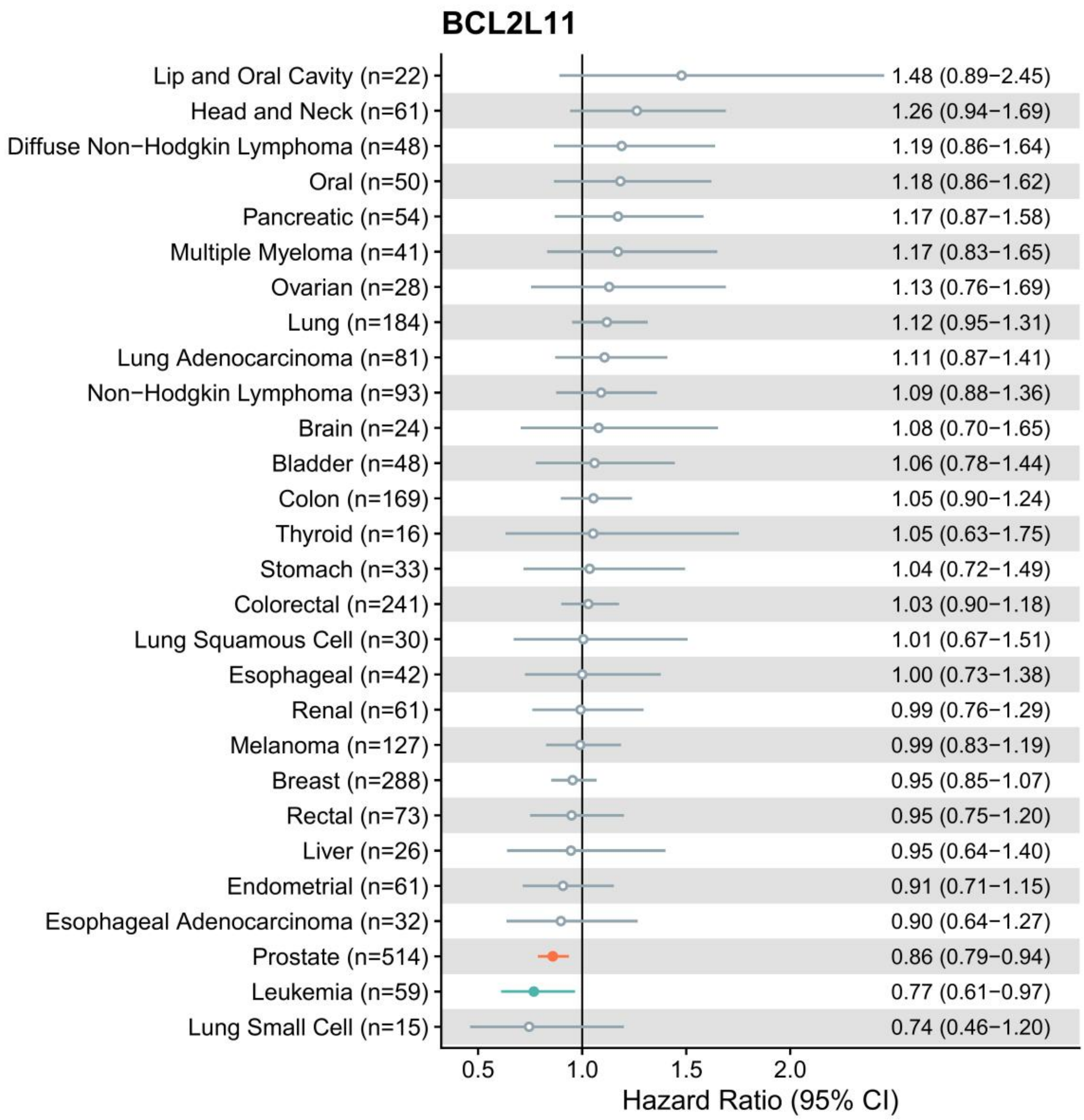

Figure S11: Observational analysis of BCL2L11 in relation to risk for prostate cancer and other cancers diagnosed more than seven years after recruitment in the UK Biobank cohort study. Hazard ratios (HRs) and 95% confidence intervals were estimated using Cox proportional hazards models, using the fully adjusted model as described in the Methods section. Results in red refer to those for risk of prostate cancer, while those in blue indicate an association with $p < 0.05$. Hollow points indicate $p > 0.05$. All p-values are two-sided. No adjustment was made for multiple comparisons. Data for this figure is provided in Source Data-Figure S9-15.

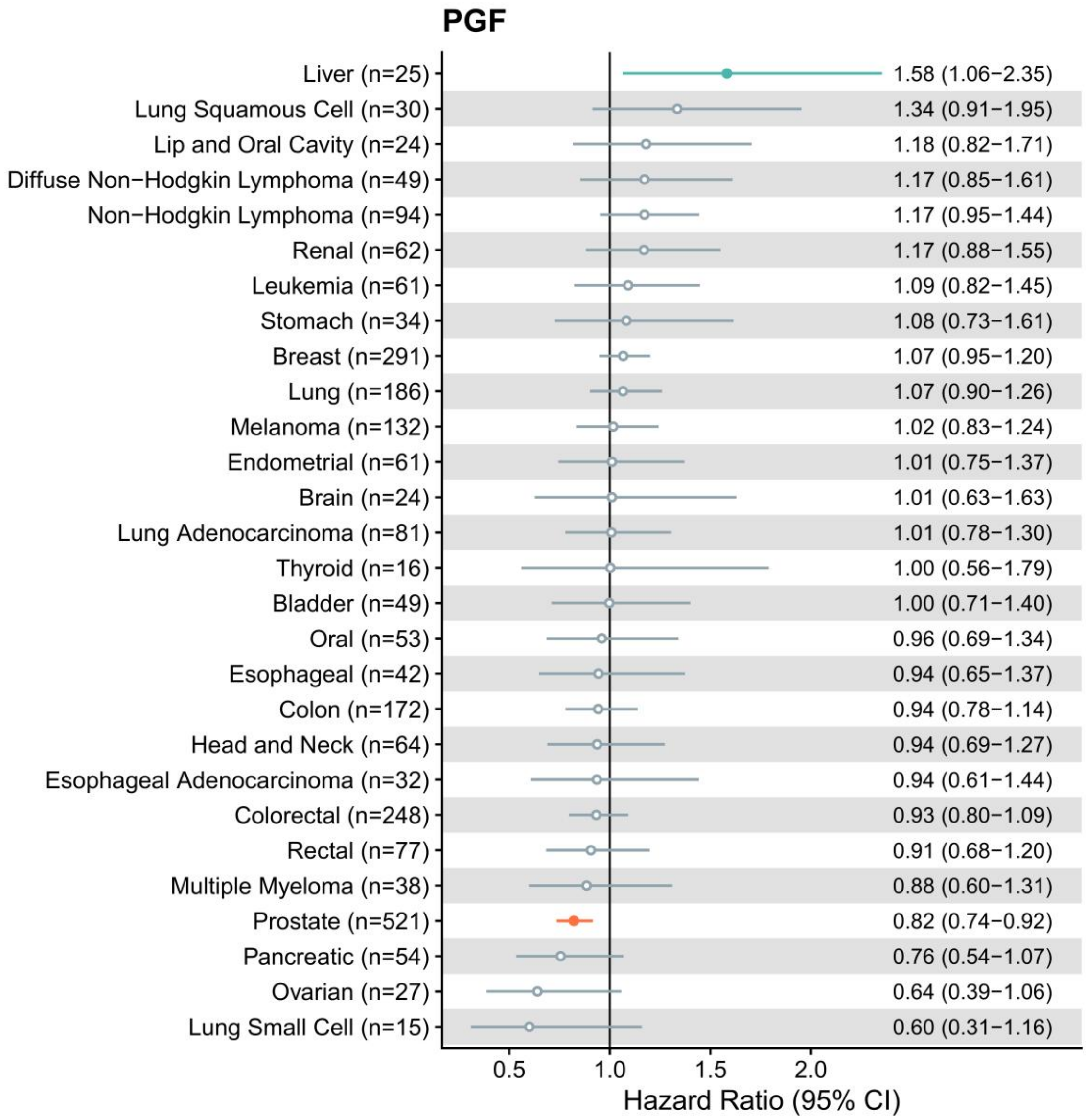

Figure S12: Observational analysis of PGF in relation to risk for prostate cancer and other cancers diagnosed more than seven years after recruitment in the UK Biobank cohort study. Hazard ratios (HRs) and 95% confidence intervals were estimated using Cox proportional hazards models, using the fully adjusted model as described in the Methods section. Results in red refer to those for risk of prostate cancer, while those in blue indicate an association with $p < 0.05$. Hollow points indicate $p > 0.05$. All p-values are two-sided. No adjustment was made for multiple comparisons. Data for this figure is provided in Source Data-Figure S9-15.

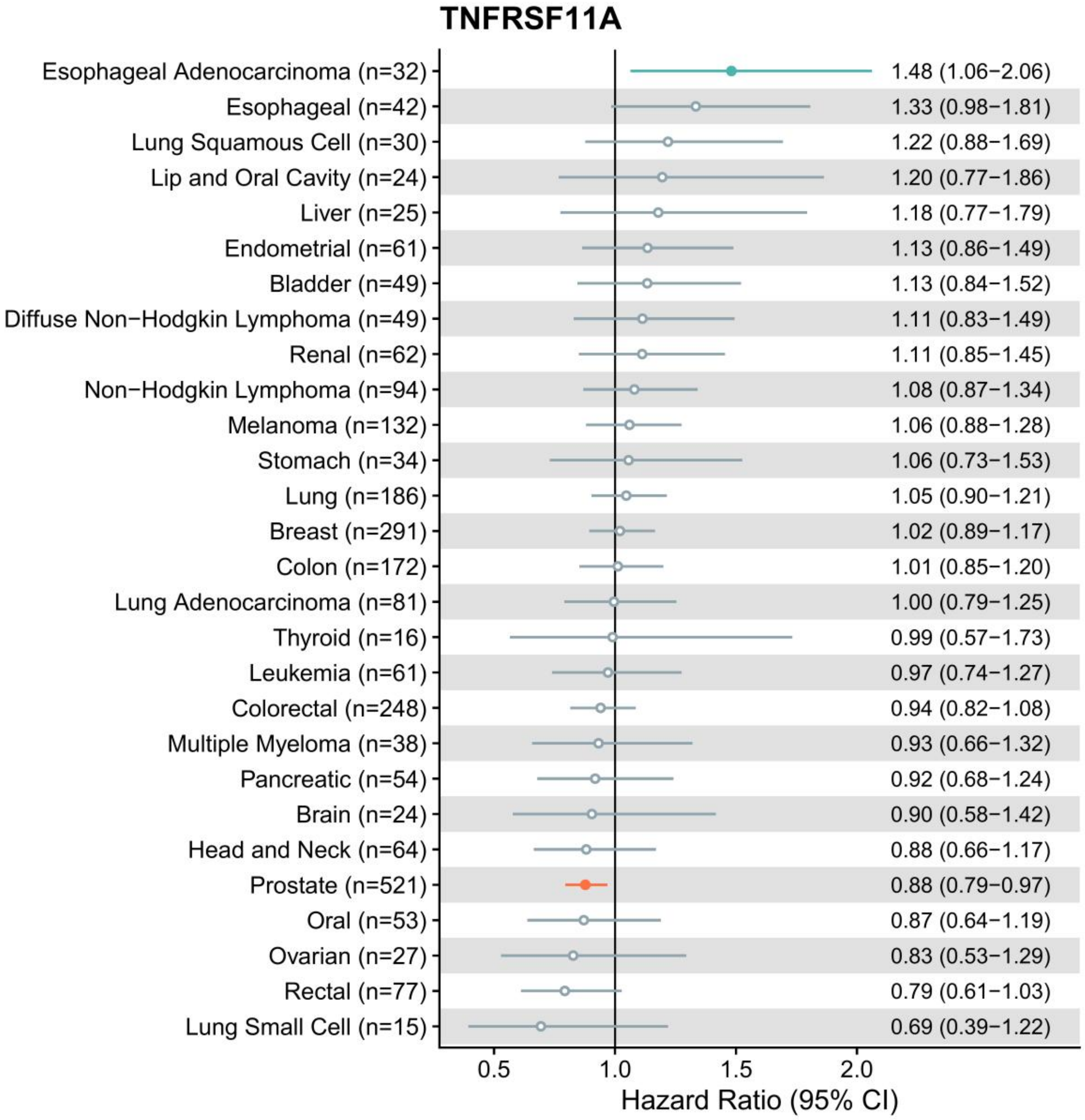

Figure S13: Observational analysis of TNFRSF11A in relation to risk for prostate cancer and other cancers diagnosed more than seven years after recruitment in the UK Biobank cohort study.

Hazard ratios (HRs) and 95% confidence intervals were estimated using Cox proportional hazards models, using the fully adjusted model as described in the Methods section. Results in red refer to those for risk of prostate cancer, while those in blue indicate an association with $p < 0.05$. Hollow points indicate $p > 0.05$. All p-values are two-sided. No adjustment was made for multiple comparisons. Data for this figure is provided in Source Data-Figure S9-15.

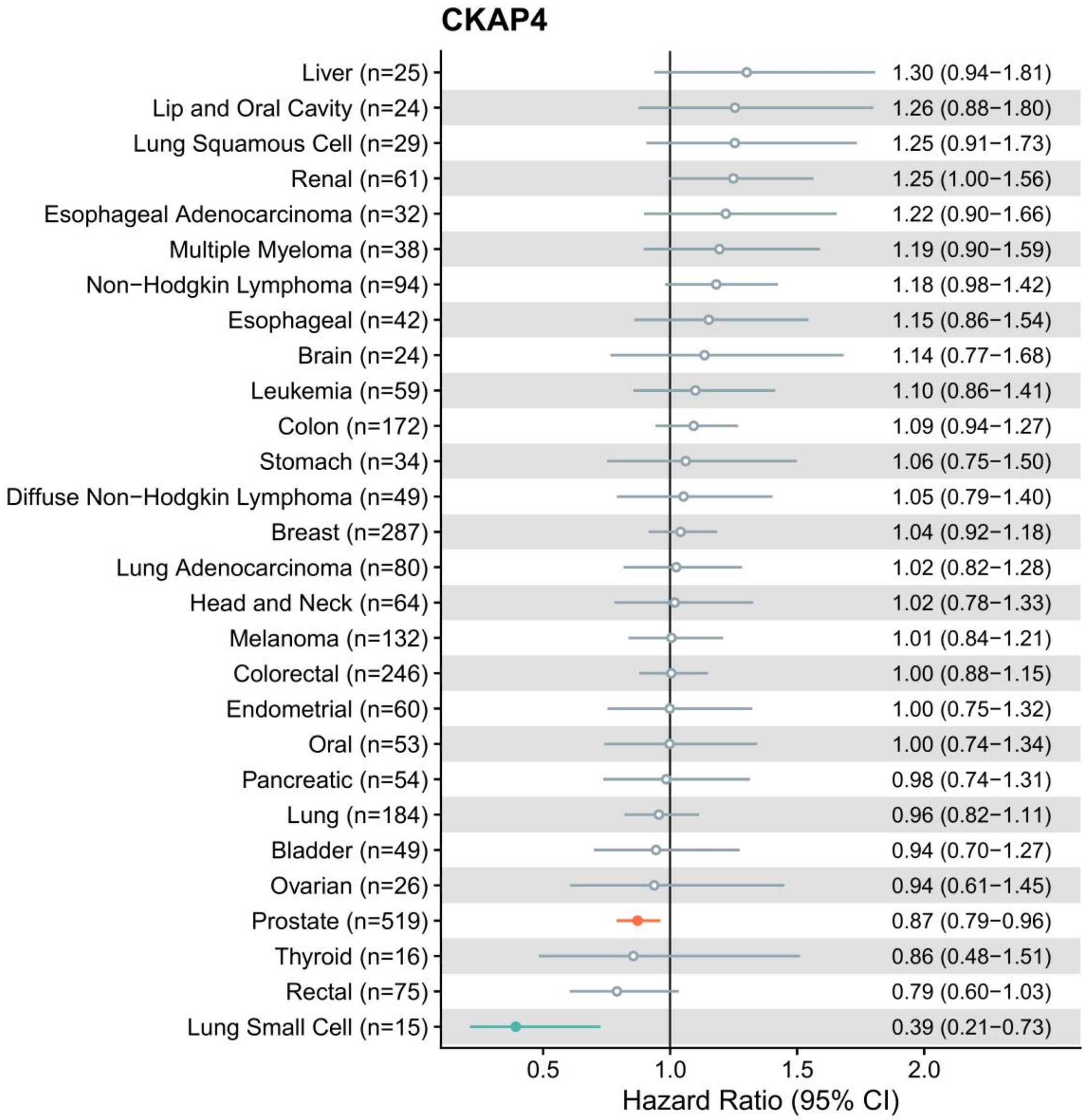

Figure S14: Observational analysis of CKAP4 in relation to risk for prostate cancer and other cancers diagnosed more than seven years after recruitment in the UK Biobank cohort study. Hazard ratios (HRs) and 95% confidence intervals were estimated using Cox proportional hazards models, using the fully adjusted model as described in the Methods section. Results in red refer to those for risk of prostate cancer, while those in blue indicate an association with $p < 0.05$. Hollow points indicate $p > 0.05$. All p-values are two-sided. No adjustment was made for multiple comparisons. Data for this figure is provided in Source Data-Figure S9-15.

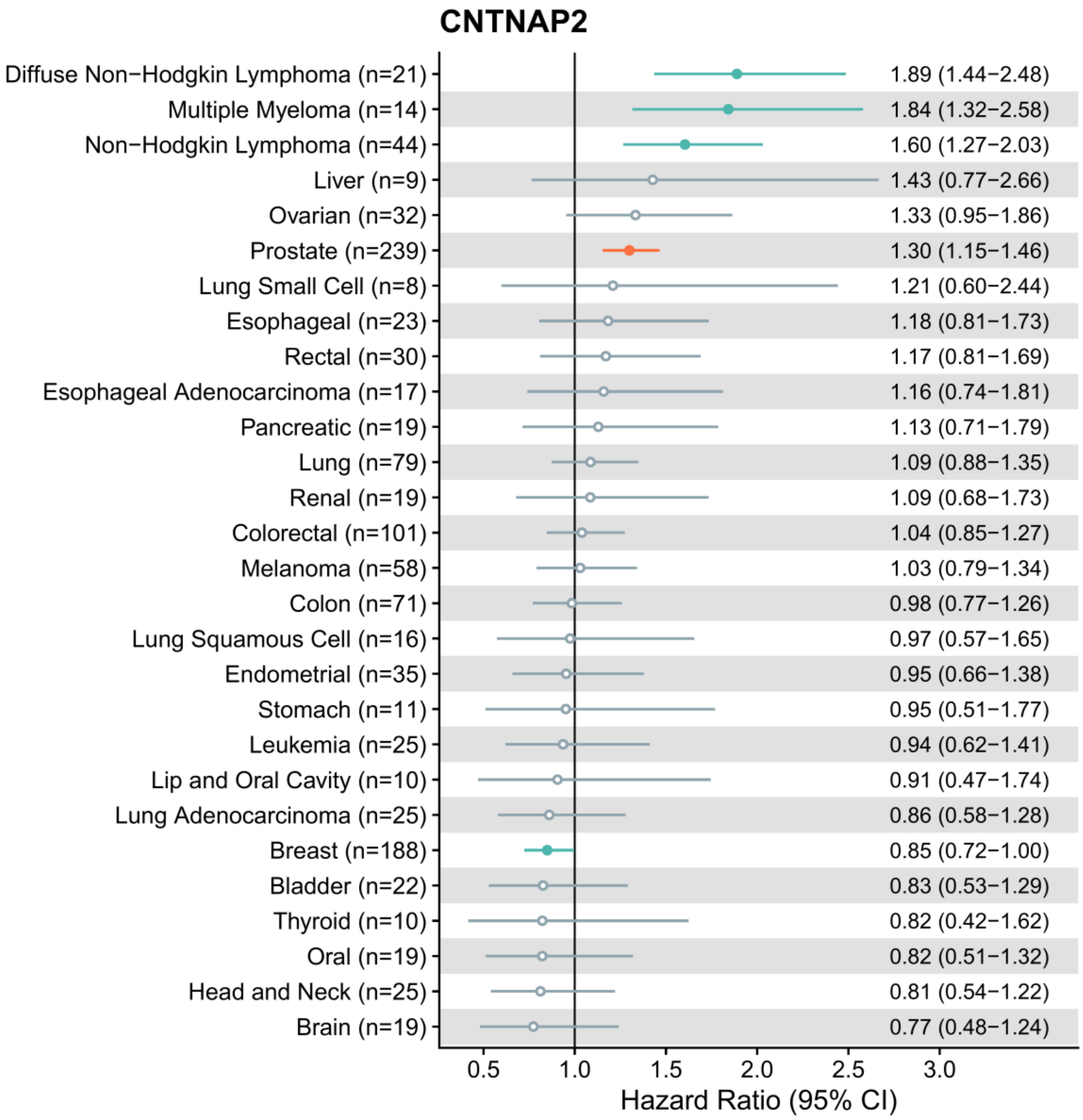

Figure S15: Observational analysis of CNTNAP2 in relation to risk for prostate cancer and other cancers diagnosed less than three years after recruitment in the UK Biobank cohort study. Hazard ratios (HRs) and 95% confidence intervals were estimated using Cox proportional hazards models, using the fully adjusted model as described in the Methods section. Results in red refer to those for risk of prostate cancer, while those in blue indicate an association with $p < 0.05$. Hollow points indicate $p > 0.05$. All p-values are two-sided. No adjustment was made for multiple comparisons. Data for this figure is provided in Source Data-Figure S9-15.

S4.2 Cross cancer analysis based on MR results

Summary of GWAS used in MR analyses

Bladder (ICD-10: C67). GWAS summary statistics were obtained from a meta-analysis involving 8,988 cases and 11,978 controls of European ancestry.[3] Breast (ICD-10: C50). GWAS summary statistics were obtained for overall breast cancer (133,384 cases and 113,789 controls) and for breast cancer stratified by oestrogen receptor status (ER-positive: 69,501 cases and 105,974 controls; ER-negative: 21,468 cases and 105,974 controls) and by

molecular subtypes (luminal B-like, luminal A-like, luminal /HER2-negative-like, HER2-enrichedlike, and triple negative).[4] All analyses were in participants of European ancestry. Colorectum (ICD-10: C18-C20). GWAS summary statistics were taken from a meta-analysis involving 78,473 cases and 107,143 controls of European ancestry.[5] Endometrium (ICD-10: C54.1). GWAS summary statistics came from the Endometrial Cancer Association Consortium, with 121,885 participants of European ancestry (12,906 cases and 108,979 controls).[6] Head and Neck. GWAS summary statistics for head and neck cancer (6,034 cases and 6,585 controls) based on ICD-10 codes: oral cavity (2,641 cases) (C02.0–C02.9, C03.0–C03.9, C04.0–C04.9 and C05.0–C06.9) and oropharynx (2,990 cases) (C01.9, C02.4 and C09.0–C10.9). The study population included individuals from Europe (45.3%), North America (43.9%) and South America (10.8%).[7] Kidney (ICD-10: C64). GWAS summary statistics for kidney cancer was obtained from a meta-analysis involving 25,890 cases and 743,585 controls of European ancestry.[8] Lung (ICD-10: C34). GWAS summary statistics for overall lung cancer came from a meta-analysis of a case-control GWAS for lung cancer (29,266 cases and 56,450 controls) and a GWAS of family history of lung cancer in the UK Biobank (48,843 proxy cases and 195,387 controls), with a total effective sample size of 41,477 cases and 105,297 controls.[9] We also obtained summary data by smoking status (never: 2,355 cases and 7,504 controls; ever: 23,223 cases and 16,964 controls), and by histological subtypes from the INTEGRAL-ILCCO cohort (adenocarcinoma: 11,273 cases and 55,483 controls; squamous cell carcinoma: 7,426 cases and 55,627 controls; small cell carcinoma: 2,664 cases and 21,444 controls).[10] All analyses were in participants of European descent. Ovary (ICD-10: C56). GWAS summary statistics were obtained from the Ovarian Cancer Association Consortium (OCAC), for invasive epithelial ovarian cancer (25,509 cases and 40,941 controls) and for histological subtypes: high-grade serous carcinoma (13,037 cases), endometrioid carcinoma (2,810 cases), invasive mucinous ovarian cancer (1,417 cases), clear cell carcinoma (1,366 cases), and high/low grade serous carcinoma (14,049 cases), with 40,941 controls for all subtypes.[11] All analyses were in European descents. Pancreas (ICD-10: C25). GWAS data were extracted from the PanScan and PanC4 consortia (with 7,638 cases and 7,364 controls of European ancestry) via the National Center for Biotechnology Information database of Genotypes and Phenotypes (dbGaP; Study Accession: phs000206.v3.p2 and phs000648.v1.p1; project reference #9314).[12] Additional cancer endpoints. GWAS summary statistics were obtained from meta-analyses of European descents from FinnGen (412,181 participants) and UKB (42,0531 participants) for the following cancers: oesophageal (ICD-10: C15, 1594 cases), stomach (C16, 2187 cases), thyroid (C73, 2334 cases), uterine (C54, 4454 cases), brain (C71, 1486 cases), melanoma of skin (C43, 5792 cases), and blood cancers including follicular (C82, 1717 cases), non-follicular (C83, 3903 cases), diffuse B-cell (C83, 1811 cases), and mature T/NK lymphoma (C84, 514 cases), multiple myeloma (C90, 2097 cases), lymphoid leukaemia (C91, 2419 cases), and myeloid leukaemia (C92, 1301 cases).[13-15]

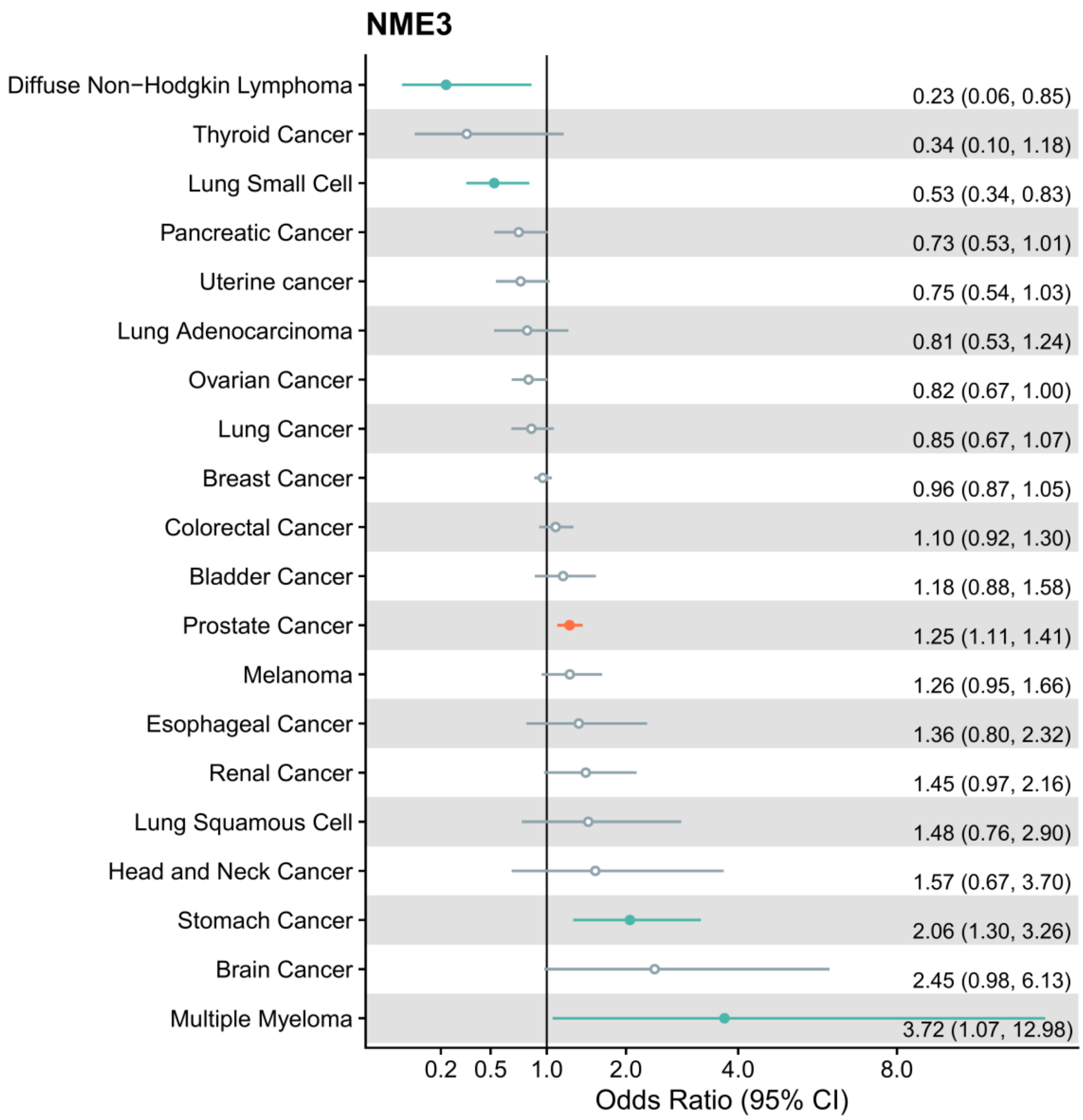

Figure S16: MR analysis of NME3 in relation to risk for prostate cancer and other cancers. Odds ratios (ORs) and 95% confidence intervals were estimated using cis-pQTL-based Wald ratio Mendelian randomization analyses and represent the effect of genetically predicted protein levels on cancer risk. The number of cases for each cancer type are as follows: Bladder Cancer (n = 8,988), Brain Cancer (n = 1,486), Breast Cancer (n = 133,384), Colorectal Cancer (n = 78,473), Diffuse Non-Hodgkin Lymphoma (n = 1,811), Esophageal Cancer (n = 1,594), Head and Neck Cancer (n = 6,034), Lung Adenocarcinoma Cancer (n = 11,273), Lung Cancer (n = 41,477), Lung Small Cell Cancer (n = 2,664), Lung Squamous Cell Cancer (n = 7,426), Melanoma (n = 5,792), Multiple Myeloma Cancer (n = 2,097), Ovarian Cancer (n = 25,509), Pancreatic Cancer (n = 7,638), Prostate Cancer (n = 85,554), Renal Cancer (n = 25,890), Stomach Cancer (n = 2,187), Thyroid Cancer (n = 2,334), Uterine Cancer (n = 4,454). Results in red refer to those for risk of prostate cancer, while those in blue indicate an association with $p < 0.05$. Hollow points indicate $p > 0.05$. No adjustment was made for multiple comparisons. Data for this figure is provided in Source Data-Figure S16-17.

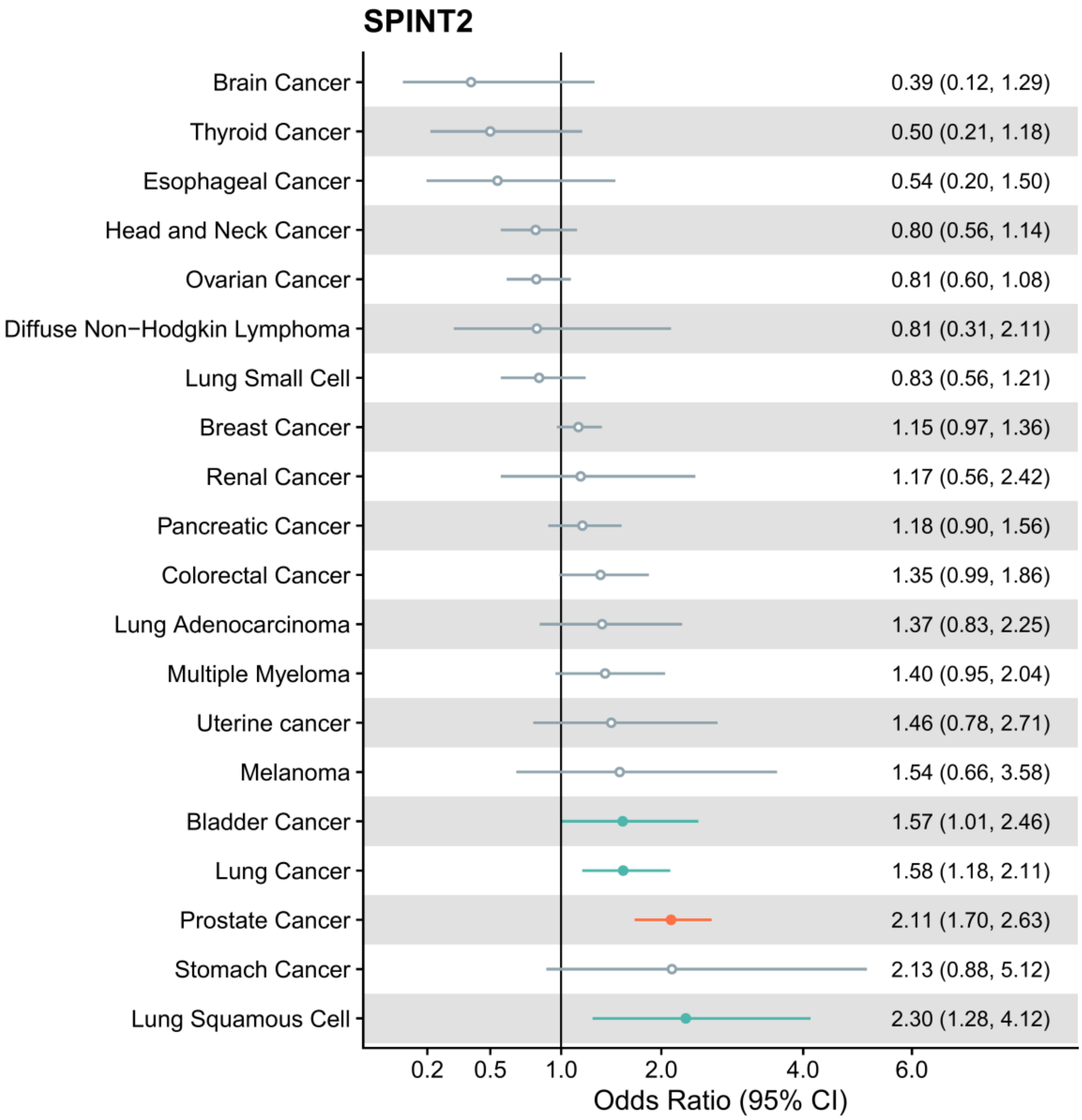

Figure S17: MR analysis of SPINT2 in relation to risk for prostate cancer and other cancers. Odds ratios (ORs) and 95% confidence intervals were estimated using cis-pQTL-based Wald ratio Mendelian randomization analyses and represent the effect of genetically predicted protein levels on cancer risk. The number of cases for each cancer type are as follows: Bladder Cancer (n = 8,988), Brain Cancer (n = 1,486), Breast Cancer (n = 133,384), Colorectal Cancer (n = 78,473), Diffuse Non-Hodgkin Lymphoma (n = 1,811), Esophageal Cancer (n = 1,594), Head and Neck Cancer (n = 6,034), Lung Adenocarcinoma Cancer (n = 11,273), Lung Cancer (n = 41,477), Lung Small Cell Cancer (n = 2,664), Lung Squamous Cell Cancer (n = 7,426), Melanoma (n = 5,792), Multiple Myeloma Cancer (n = 2,097), Ovarian Cancer (n = 25,509), Pancreatic Cancer (n = 7,638), Prostate Cancer (n = 85,554), Renal Cancer (n = 25,890), Stomach Cancer (n = 2,187), Thyroid Cancer (n = 2,334), Uterine Cancer (n = 4,454). Results in red refer to those for risk of prostate cancer, while those in blue indicate an association with $p < 0.05$. Hollow points indicate $p > 0.05$. No adjustment was made for multiple comparisons. Data for this figure is provided in Source Data-Figure S16-17.

S5: Genotyping, exome genetic score construction, and variant quality in MEC

The Multiethnic Cohort (MEC) was genotyped primarily using Illumina SNP arrays, with most remaining samples genotyped during 2020–2021 using the Illumina Global Diversity Array (GDA), including MEGA content. No exome sequencing or exome chip data were available for MEC.

The PARP1 Exome Genetic Score (ExGS) was derived in UK Biobank using exome sequencing data. For MEC, the PARP1 ExGS was constructed using the same PARP1 single-nucleotide variants (SNVs) identified in the UK Biobank–derived instrument, with scoring allele effect directions aligned to increasing PARP1 protein levels. All SNVs included in the PARP1 ExGS were common variants (minor allele frequency [MAF] >1%).

MEC genotypes underwent extensive quality control followed by ancestry-specific imputation using the 1000 Genomes Project and TOPMed reference panels. For variants with MAF >1%, imputation quality in MEC was high across racial and ethnic groups (mean R^2 ≥0.8), and none of the PARP1 SNVs included in the ExGS were poorly imputed. Rare variants (MAF <0.5%), which are known to have unreliable genotype clustering on SNP arrays, were not included in the PARP1 ExGS in MEC. Additional details on the SNVs included in the PARP1 panel is provided below.

Table S1. Single-nucleotide variants (SNVs) included in the PARP1 exome genetic score (ExGS).

Gene	Protein	MAF	cis/trans	Effect	Genotype	Scoring allele	weight
APOE	PARP1	0.08202897	trans	missense_variant	19:44908822:C:T	T	0.065441016
NLRP12	PARP1	0.20886541	trans	missense_variant	19:53824059:C:A	A	0.161935763
PARP1	PARP1	0.15122104	cis	synonymous_variant	1:226402257:G:A	G	0.155592445
SH2B3	PARP1	0.48421087	trans	missense_variant	12:111446804:T:C	T	0.04553322
LILRB5	PARP1	0.3975339	trans	missense_variant	19:54255498:T:C	T	0.038976393

Additional supplementary tables

Table S2. Baseline characteristics of UK Biobank cohort participants and those diagnosed with prostate cancer in the observational analysis.

Characteristic	All men N = 21,481	Men who developed prostate cancer N = 1,147
**Age at blood collection (year), Mean (SD)**	56.6 (8.4)	61.6 (5.7)
**Weight (kg), Mean (SD)**	85.1 (13.8)	84.1 (12.5)
**Height (cm), Mean (SD)**	175.6 (6.9)	175.0 (6.5)
**Body Mass Index (kg/m^2), Mean (SD)**	27.6 (4.0)	27.5 (3.7)
**Alcohol (g/day) , Mean (SD)**	24.5 (23.1)	23.1 (21.2)
**Smoking Status, n (%)**		
Never	10,552 (49.1)	525 (45.8)
Previous	8,132 (37.9)	512 (44.6)
Current	2,681 (12.5)	103 (9.0)
Unknown	116 (0.5)	7 (0.6)
**Educational Attainment, n (%)**		
College or university degree/other professional /vocational qualification	10,251 (47.7)	563 (49.1)
A levels/AS levels or equivalent	2,190 (10.2)	115 (10.0)
O levels/GCSEs or equivalent	5,088 (23.7)	224 (19.5)
Other/unknown	3,952 (18.4)	245 (21.4)
**Townsend deprivation index, n (%)**		
Most affluent	4,381 (20.4)	282 (24.6)
Moderately Affluent	4,240 (19.7)	197 (17.2)
Less Deprived	4,153 (19.3)	233 (20.3)
Moderately Deprived	4,275 (19.9)	240 (20.9)
Most deprived	4,398 (20.5)	195 (17.0)
Unknown	34 (0.2)	0
**Family history of prostate, n (%)**	1,364 (6.3)	131 (11.4)
**Region, n (%)**		
East Midlands	1,701 (7.9)	100 (8.7)
London	2,915 (13.6)	192 (16.7)
North-Eastern England	2,553 (11.9)	122 (10.6)
North-West England	3,467 (16.1)	150 (13.1)
Scotland	1,463 (6.8)	74 (6.5)
South-East England	1,571 (7.3)	109 (9.5)
South-West England	1,548 (7.2)	82 (7.1)
Wales	892 (4.2)	29 (2.5)
West Midlands	1,978 (9.2)	113 (9.9)
Yorkshire & the Humber	3,393 (15.8)	176 (15.3)
**Time to diagnosis, n (%)**		
<3 years	---	242 (21.1)
3-7 years	---	372 (32.4)
>7 years	---	533 (46.5)

Table S3. Baseline characteristics of UK Biobank cohort and those diagnosed with prostate cancer in EXOME analysis

Characteristic	All men N = 161,190	Men who developed prostate cancer N = 8,610
**Age at blood collection (year), Mean (SD)**	56.7 (8.1)	61.5 (5.7)
**Weight (kg), Mean (SD)**	85.6 (13.8)	84.6 (12.7)
**Height (cm), Mean (SD)**	176.0 (6.7)	175.5 (6.6)
**Body Mass Index (kg/m^2), Mean (SD)**	27.6 (4.0)	27.4 (3.7)
**Alcohol (g/day) , Mean (SD)**	24.9 (23.2)	23.8 (21.4)
**Smoking Status, n (%)**		
Never	80,466 (49.9)	4,185 (48.6)
Previous	61,033 (37.9)	3,669 (42.6)
Current	19,170 (11.9)	723 (8.4)
Unknown	521 (0.3)	33 (0.4)
**Educational Attainment, n (%)**		
College or university degree/other professional /vocational qualification	75,566 (46.9)	4,164 (48.4)
A levels/AS levels or equivalent	16,870 (10.5)	855 (9.9)
O levels/GCSEs or equivalent	40,614 (25.2)	1,844 (21.4)
Other/unknown	28,140 (17.5)	1,747 (20.3)
**Townsend deprivation index, n (%)**		
Most affluent	28,300 (17.6)	1,168 (13.6)
Moderately Affluent	30,985 (19.2)	1,552 (18.0)
Less Deprived	32,867 (20.4)	1,854 (21.5)
Moderately Deprived	33,954 (21.1)	1,945 (22.6)
Most deprived	34,888 (21.6)	2,082 (24.2)
Unknown	196 (0.1)	9 (0.1)
**Family history of prostate, n (%)**	11,008 (6.8)	890 (10.3)
**Region, n (%)**		
London	16,021 (9.9)	924 (10.7)
Wales	6,993 (4.3)	242 (2.8)
North-West England	25,183 (15.6)	1,247 (14.5)
North-Eastern England	20,857 (12.9)	1,040 (12.1)
Yorkshire & the Humber	25,635 (15.9)	1,402 (16.3)
West Midlands	15,260 (9.5)	840 (9.8)
East Midlands	11,600 (7.2)	654 (7.6)
South-East England	13,981 (8.7)	908 (10.5)
South-West England	14,416 (8.9)	755 (8.8)
Scotland	11,244 (7.0)	598 (6.9)
**Time to diagnosis, n (%)**		
<3 years	---	1,723 (20.0)
3-7 years	---	2,857 (33.2)
>7 years	---	4,030 (46.8)

Table S4. Summary of Number of cis-pQTL per protein in MR analysis

Number of cis-pQTL per protein	Overall Prostate Cancer	Aggressive Prostate Cancer	Early-onset Prostate Cancer
1	110 (38.60)	108 (39.13)	108 (39.13)
2	94 (32.98)	87 (31.52)	88 (31.88)
3	44 (15.44)	45 (16.30)	44 (15.94)
4	23 (8.07)	23 (8.33)	23 (8.33)
5	7 (2.46)	7 (2.54)	7 (2.54)
6	4 (1.40)	3 (1.09)	3 (1.09)
7	2 (0.70)	2 (0.72)	2 (0.72)
8	1 (0.35)	1 (0.36)	1 (0.36)
Total	285	276	276

Table S5. Summary of protein measurements below lower limit of detection (LOD) in EPIC and UKBB cohorts

LOD Category	Number of Proteins (%)	
	EPIC	UKBB
Samples below LOD < 1%	267 (73%)	293 (80%)
Samples below LOD 1% to 5%	19 (5%)	11 (3%)
Samples below LOD 5% to 10%	20 (5%)	8 (2%)
Samples below LOD 10% to 20%	8 (2%)	6(2%)
Samples below LOD 20% to 50%	21 (6%)	16 (4%)
Samples below LOD 50% to 70%	13 (4%)	8 (2%)
Samples below LOD 70% to 90%	16 (4%)	19 (5%)
Samples below LOD > 90%	4 (1%) – ARNT, IL4, PREB, VASH1	7 (2%) – IL2, DGKZ, ARNT, IL1A, IL24, IL4, NCLN

Table S6. Summary of missingness for proteins measured in EPIC and UKBB cohorts

Missingness Category	Number of Proteins (%)	
	EPIC	UKBB
<1%	174 (48%)	0 (0%)
1% to 3%	110 (29%)	247 (67%)
3% to 5%	81 (22%)	118 (32%)
>5%	3 (1%)- LY6D, IL1A, IL1RN	3 (1%)- MMP1, ENPP7, IL1B

References

1. PEA – a high-multiplex immunoassay technology with qPCR or NGS readout2020. https://www.olink.com/content/uploads/2021/09/olink-white-paper-pea-a-high-multiplex-immunoassay-technology-with-qpcr-or-ngs-readout-v1.0.pdf (accessed.

2. Scutari M, Denis J-B. Bayesian networks: with examples in R: Chapman and Hall/CRC; 2021.

3. Rothman N, Garcia-Closas M, Chatterjee N, et al. A multi-stage genome-wide association study of bladder cancer identifies multiple susceptibility loci. *Nat Genet* 2010; **42**(11): 978-84.

4. Michailidou K, Lindström S, Dennis J, et al. Association analysis identifies 65 new breast cancer risk loci. *Nature* 2017; **551**(7678): 92-4.

5. Fernandez-Rozadilla C, Timofeeva M, Chen Z, et al. Deciphering colorectal cancer genetics through multi-omic analysis of 100,204 cases and 154,587 controls of European and east Asian ancestries. *Nat Genet* 2023; **55**(1): 89-99.

6. O'Mara TA, Glubb DM, Amant F, et al. Identification of nine new susceptibility loci for endometrial cancer. *Nat Commun* 2018; **9**(1): 3166.

7. Lesseur C, Ferreiro-Iglesias A, McKay JD, et al. Genome-wide association meta-analysis identifies pleiotropic risk loci for aerodigestive squamous cell cancers. *PLoS Genet* 2021; **17**(3): e1009254.

8. Purdue MP, Dutta D, Machiela MJ, et al. Multi-ancestry genome-wide association study of kidney cancer identifies 63 susceptibility regions. *Nat Genet* 2024; **56**(5): 809-18.

9. Gabriel AAG, Atkins JR, Penha RCC, et al. Genetic Analysis of Lung Cancer and the Germline Impact on Somatic Mutation Burden. *J Natl Cancer Inst* 2022; **114**(8): 1159-66.

10. McKay JD, Hung RJ, Han Y, et al. Large-scale association analysis identifies new lung cancer susceptibility loci and heterogeneity in genetic susceptibility across histological subtypes. *Nat Genet* 2017; **49**(7): 1126-32.

11. Phelan CM, Kuchenbaecker KB, Tyrer JP, et al. Identification of 12 new susceptibility loci for different histotypes of epithelial ovarian cancer. *Nat Genet* 2017; **49**(5): 680-91.

12. Klein AP, Wolpin BM, Risch HA, et al. Genome-wide meta-analysis identifies five new susceptibility loci for pancreatic cancer. *Nat Commun* 2018; **9**(1): 556.

13. Kurki MI, Karjalainen J, Palta P, et al. FinnGen provides genetic insights from a well-phenotyped isolated population. *Nature* 2023; **613**(7944): 508-18.

14. Karczewski KJ, Gupta R, Kanai M, et al. Pan-UK Biobank GWAS improves discovery, analysis of genetic architecture, and resolution into ancestry-enriched effects. *medRxiv* 2024: 2024.03.13.24303864.

15. FinnGen. Documentation of R10 release. 2023. https://finngen.gitbook.io/documentation/ (accessed 13/12/2024.
