# Supplementary material for: Prospective and genetic analyses implicate lower immunosurveillance in the aetiology of prostate cancer
Source: Nat Commun. 2026 Nov 10;17:10698. doi: 10.1038/s41467-026-77378-3 (PMC13752312; doi:10.1038/s41467-026-77378-3)
Supplement: Supplementary file 2 [file 41467_2026_77378_MOESM2_ESM.pdf]

Description of Additional Supplementary Files

Supplementary Dataset 1. Associations of a 1-SD increase in selected inflammatory proteins with prostate cancer risk: analyses from the prospective EPIC and UK Biobank cohorts, meta-analysis, exome analysis, and Mendelian randomisation analysis.

This table presents the associations of a one standard deviation (1-SD) increase in circulating protein levels with risk of prostate cancer across multiple analytical approaches. Observational associations are expressed as odds ratios (ORs) and hazard ratios (HRs) with 95% confidence intervals (95% CIs) from conditional logistic regression models (EPIC) and Cox proportional hazards models (UK Biobank). Meta-analysed results are expressed as relative risks (RRs) and 95% CIs, derived using inverse-variance weighted meta-analysis. Exome-wide genetic score analyses used logistic regression. Mendelian randomisation analyses used cis-pQTL-based Wald ratio estimates. Results are presented overall and stratified by time from blood collection to diagnosis (< 3 years and > 7 years). Selected proteins are those that were statistically significant in the overall, short lag-time (< 3 years), or long lag-time (> 7 years) meta-analyses of EPIC and UK Biobank.

Supplementary Dataset 2. Protein-cancer associations from minimally adjusted, and fully -adjusted, and subgroup analyses by time-to-diagnosis and cancer subtype from EPIC, UK Biobank, and meta-analysis.

This table provides complete results for the association of all 368 immune and inflammation-related proteins with risk of prostate cancer from observational analyses. Results are presented from both minimally adjusted models (adjusted for exact age at blood collection) and fully adjusted models (additionally adjusted for smoking, alcohol intake, BMI, education level, and diabetes status) in EPIC and UK Biobank. Subgroup analyses are presented stratified by time from blood collection to diagnosis (< 3 years, 3–7 years, and > 7 years) and cancer subtypes (tumour stage, histological grade, and disease aggressiveness) from EPIC and UK Biobank, and from inverse-variance weighted meta-analysis. Associations are expressed as ORs (EPIC), HRs (UK Biobank), or RRs (meta-analysis) and 95% CIs. All p-values are two-sided and were not adjusted for multiple comparisons unless otherwise stated. This table is also referenced in analyses presented in Figures 2, 3, supplementary information S1, and S5.

Supplementary Dataset 3. Protein-cancer associations from cis-pQTL exome-wide genetic score analyses.

This table presents results from the cis-pQTL-based exome-wide genetic score (exGS) analysis for 262 proteins. Associations of a one standard deviation increase in genetically predicted protein levels with overall prostate cancer risk are expressed as ORs and 95% CIs, estimated using logistic regression in 161,190 European men from the UK Biobank. Models were adjusted for age and the first 10 genetic principal components of ancestry. All p-values are two-sided. Conventional significance was defined as $p < 0.05$; Bonferroni correction ($p < 0.05/262$) was applied to define statistical significance after correction for multiple testing.

Supplementary Dataset 4. Protein-cancer associations from combined cis- and trans-pQTL exome-wide genetic score analyses.

This table presents the full results from the combined cis- and trans-pQTL exome-wide genetic score (exGS) analysis for 347 proteins. Associations of a one standard deviation increase in genetically predicted protein levels with overall prostate cancer risk are expressed as ORs and 95% CIs, estimated using logistic regression in 161,190 European men from the UK Biobank. Models were adjusted for age and the first 10 genetic principal components of ancestry. All p-values are two-sided. Conventional significance was defined as $p < 0.05$; Bonferroni correction ($p < 0.05/347$) was applied to define statistical significance after correction for multiple testing.

Supplementary Dataset 5. Protein-cancer associations from Mendelian randomisation analyses.

This table presents the full Mendelian randomisation (MR) results for 285 proteins with available cis-pQTLs in relation to overall, aggressive, and early-onset prostate cancer risk. Primary estimates are ORs and 95% CIs per one standard deviation increase in genetically predicted protein levels, derived using the Wald ratio method. For proteins passing Bonferroni correction ($p < 0.05/285$), results from Bayesian colocalisation analyses are also included, reporting posterior probabilities for a shared causal variant (PP4) between protein and prostate cancer GWAS signals. All p-values are two-sided.

Supplementary Dataset 6. Colocalisation results for Mendelian randomisation associations passing Bonferroni correction.

This table provides the results of Bayesian colocalisation analyses for proteins whose MR associations with prostate cancer passed Bonferroni correction ($p < 0.05/285$). For each protein, colocalisation was assessed within a 250 kb region around the index cis-pQTL using conventional colocalisation and the Sum of Single Effects (coloc SuSiE) method. Posterior probabilities for five hypotheses (H0–H4) are reported, where PP4 > 0.70 was accepted as evidence for a shared causal variant between the protein and prostate cancer GWAS signal.

Supplementary Dataset 7. Reverse Mendelian randomisation analysis results for significant target proteins.

This table presents the results of reverse Mendelian randomisation analyses for proteins with significant associations with prostate cancer after Bonferroni correction. Prostate cancer was modelled as the exposure and protein concentrations as the outcome. Results are reported using multiple MR methods: random-effects inverse-variance weighted, weighted median, and MR-PRESSO. Consistent evidence across methods was required to indicate a notable reverse MR association. All p-values are two-sided.
