# Supplementary material for: Prospective and genetic analyses implicate lower immunosurveillance in the aetiology of prostate cancer
Source: Nat Commun. 2026 Nov 10;17:10698. doi: 10.1038/s41467-026-77378-3 (PMC13752312; doi:10.1038/s41467-026-77378-3)
Supplement: Supplementary file 4 [file 41467_2026_77378_MOESM5_ESM.pdf]

nature portfolio

Peer Review File

Prospective and genetic analyses implicate lower immunosurveillance in the aetiology of prostate cancer

Corresponding Author: Dr Karl Smith-Byrne

This manuscript has been previously reviewed at another journal. This document only contains information relating to versions considered at Nature Communications.

This file contains all reviewer reports in order by version, followed by all author rebuttals in order by version.

Version 1:

Reviewer comments:

Reviewer #2

(Remarks to the Author)
The authors have addressed my concerns and revised the manuscript accordingly.

Reviewer #4

(Remarks to the Author)
The authors have adequately addressed my previous critiques. I have no further comments.

Open Access This Peer Review File is licensed under a Creative Commons Attribution 4.0 International License, which permits use, sharing, adaptation, distribution and reproduction in any medium or format, as long as you give appropriate credit to the original author(s) and the source, provide a link to the Creative Commons license, and indicate if changes were made.

In cases where reviewers are anonymous, credit should be given to 'Anonymous Referee' and the source.
The images or other third party material in this Peer Review File are included in the article's Creative Commons license, unless indicated otherwise in a credit line to the material. If material is not included in the article's Creative Commons license and your intended use is not permitted by statutory regulation or exceeds the permitted use, you will need to obtain permission directly from the copyright holder.
To view a copy of this license, visit https://creativecommons.org/licenses/by/4.0/

Reviewers' Comments:

Reviewer #1 (Remarks to the Author):

Major Comments

1. *The manuscript presents the results as a series of descriptive associations without a unifying conceptual framework. Clarifying the central hypothesis and how the study builds on existing knowledge would help frame the results. For example, emphasizing the protective role of immunosurveillance and its implications for PC prevention could tie the findings together.*

   Response: Our study aimed to provide a comprehensive, agnostic evaluation of inflammation and immune-related proteins in relation to prostate cancer risk, using large-scale proteomic, genetic, and replication analyses across multiple cohorts. However, based on the limited available prospective epidemiological data and on the conventional wisdom that prostate cancer is an immunologically cold tumour, we did not anticipate the potential role of immune surveillance a priori. This is why we did not lead with this hypothesis in the Introduction, and rather discussed this emerging theme in the Discussion, where we reflect on the potential protective roles of immune-related pathways, such as those involving the recruitment and activation of natural killer and T cells, and their possible relevance for prostate cancer prevention.

2. *The introduction should briefly summarize prior studies on inflammation and immune markers in PC to provide better context for the knowledge gap this study addresses. This would set the stage for the novel contributions of the current work.*

   Response: As we have described in the introduction, there are relatively few prospective observational data on PC in relation to the majority of inflammation and immune related proteins. The majority of studies investigated the association with CRP and results from meta-analyses are inconsistent. We revised the Introduction and added more details about these studies on **page 3-4, lines 106-115**.

   > The role of inflammation and the immune system in cancer aetiology is complex. While chronic inflammation is a hallmark of cancer and can promote cellular proliferation, immune and inflammation-related signalling can also detect and eliminate abnormal cells, preventing tumorigenesis via immunosurveillance.[1-3] To date for prostate cancer (PC), there is suggestive epidemiological evidence for an association with a limited number of relevant candidate proteins, such as C-reactive protein and Interleukin (IL) 6[4]. A systematic review and meta-analysis of inflammatory markers and cancer risk, which included up to seventeen studies of prostate cancer, found CRP to be the most frequently examined marker; although most studies reported null findings, pooled prospective estimates revealed a weak but statistically significant association between elevated CRP and PC risk. IL6 showed no consistent effect, with one study reporting a negative association and three studies reporting null findings.[4] More recently, a prospective analysis from the Osteoporotic Fractures in Men (MrOS) cohort reported that moderate or high IL10 levels were associated with substantially lower PC risk, with no significant associations for IL6, CRP, TNF-α, or overall inflammatory burden.[5] Despite

these insights, prospective observational data on most immune and inflammation-related proteins in PC remain scarce.[6]

3. *The authors should clarify why EPIC included fewer controls compared to UK Biobank. Could this difference impact statistical power or result comparability?*

   Response: Thank you for this comment. The difference in the number of controls comes from the underlying study designs. EPIC used a 1:1 nested case-control design, resulting in an equal number of cases and controls. In contrast, UK Biobank is a prospective cohort study, where the number of controls is determined by the overall cohort size and the number of cancer cases occurring during follow-up.

   Both the number of events and study design influence statistical power. Figure 1 shows the power analysis for EPIC and UK Biobank, based on the observed number of cases and their respective study designs. Although EPIC has lower statistical power than the UK Biobank, this does not necessarily affect the comparability of results. Both prospective studies used the same proteomic platform (Olink technology), ensuring consistency in biomarker measurements. As shown in Figure S1 of the **Supplementary Materials**, the significant point estimates are broadly consistent between the two studies, although confidence intervals are wider in EPIC, as expected.

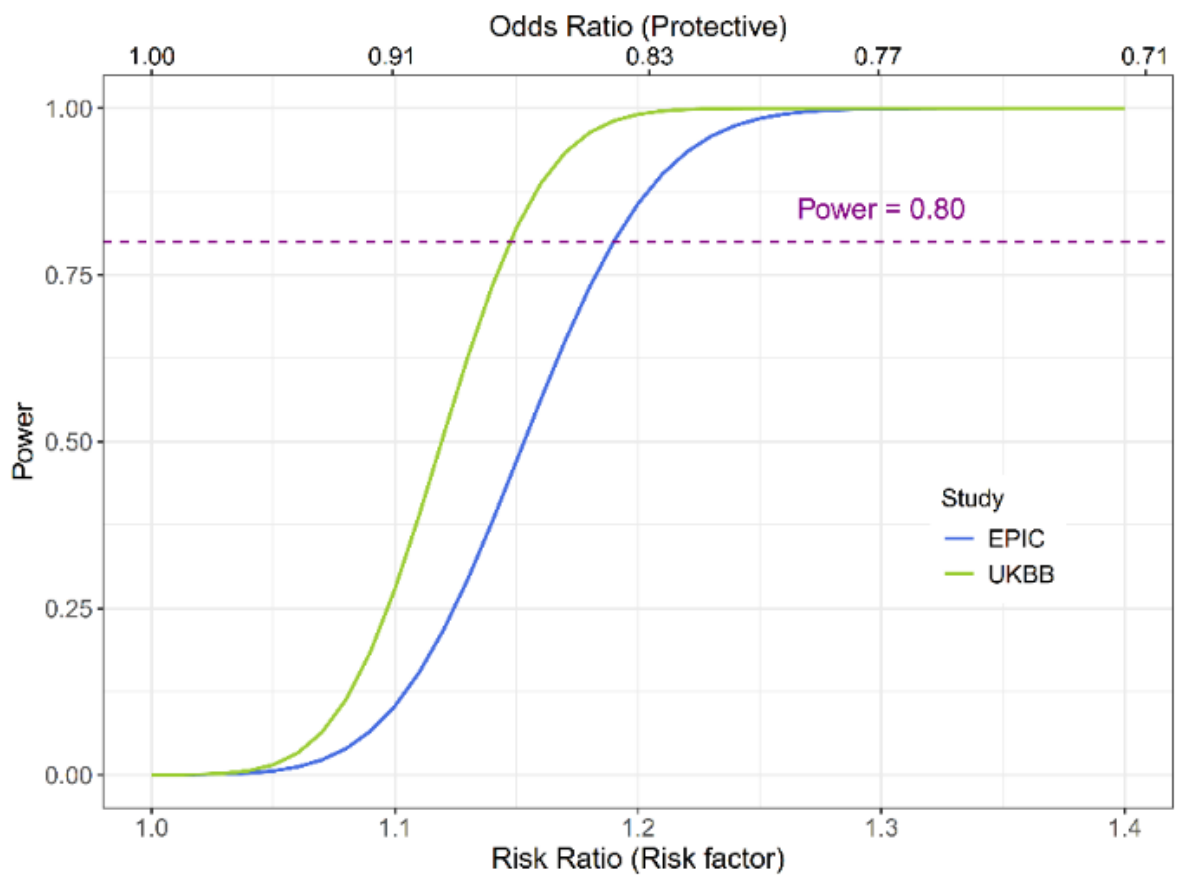

Figure 1: Statistical power analysis for EPIC and UKBB

4. *Have you considered conducting non-linear analyses for the observational data?*

   Response: We evaluated potential non-linear associations for all 368 inflammatory proteins using natural splines with three degrees of freedom. While adding non-linear terms improved model fit for some proteins, it did not materially change the overall findings. Consistent with the linear analyses, no proteins in EPIC passed the ENT threshold in the overall analysis. In the lag-time analysis, IL15 was the only protein to surpass the ENT threshold in the non-linear model, and this association was also significant under the linear model.

   In UK Biobank, non-linear analyses confirmed that CNTNAP2 and FLT3LG remained significant in the overall analysis, as observed in the linear models. In the long-lag analysis, BCL2L11 and FLT3LG-

both significant under the linear model-did not meet the ENT threshold when non-linear terms were included, likely reflecting reduced power from increased model complexity. In the short-lag analysis, CNTNAP2 remained the only protein significant under both linear and non-linear models.

5. *The manuscript does not specify the significance threshold for SNP selection in the Mendelian Randomization (MR) analysis. Including this information is essential for reproducibility and understanding the robustness of the findings.*

   Response: We have now updated the text (**page 9, line 315**) to specify that we chose genome-wide significance as the threshold to select cis pQTL for inclusion in our MR analyses.

   > "*Cis*-pQTLs meeting genome-wide significance (p-value<5e-8) and independence at r2 < 0.01 were extracted from the protein GWAS by Sun et al. involving 34,557 UKBB participants of European ancestry, and those with weak instrument strength (i.e. F-statistics < 10 [β2/σ2]) were excluded from our study."

6. *How many SNPs were available as cis-instruments for the proteins analyzed? Reporting this would clarify the strength of the genetic instruments used in MR.*

   Response: After we performed quality control for cis pQTL and harmonisation with prostate cancer GWAS summary statistics, including a search for proxy variants, cis pQTL were available for 285 out of 368 proteins in our study. More details on the eligible cis-SNPs for each protein is presented in Supplementary Table S9. We have modified the manuscript on **page 10, lines 327-328**:

   > "Harmonised SNPs were oriented to the protein-increasing allele and 285 proteins were included in cis pQTL MR analyses in this study."

   and **page 13, line 439-440**:

   > We also conducted MR analyses for PC for the 285 (out of 368) proteins in our study that had available cis-pQTLs (**see Supplementary Table S9** and **Supplementary Table S11** for complete MR results, and the number of eligible cis-SNPs for each protein, respectively).

   Supplementary Table 11 (Number of cis-pQTL per protein in MR analysis) is added to the revised version and below as well:

Number of cis-pQTL per protein	Overall Prostate Cancer	Aggressive Prostate Cancer	Early-onset Prostate Cancer
1	110 (38.60)	108 (39.13)	108 (39.13)
2	94 (32.98)	87 (31.52)	88 (31.88)
3	44 (15.44)	45 (16.30)	44 (15.94)
4	23 (8.07)	23 (8.33)	23 (8.33)
5	7 (2.46)	7 (2.54)	7 (2.54)
6	4 (1.40)	3 (1.09)	3 (1.09)
7	2 (0.70)	2 (0.72)	2 (0.72)
8	1 (0.35)	1 (0.36)	1 (0.36)
Total	285	276	276

7. *The manuscript would benefit from incorporating multivariate MR to address potential pleiotropic effects of metabolites that might confound protein-PC associations.*

   Response: We agree that multivariate MR can be a valuable tool to investigate the potential influence of pleiotropy. However, in the cis-pQTL MR analyses more often than not, there was only one genetic variant available for analyses; no multivariate MR methods are currently possible in this scenario.

8. *State explicitly whether a fixed-effects or random-effects model was used in the inverse-variance weighted (IVW) MR analysis. This improves transparency in the statistical approach.*

   Response: Cis pQTL MR analyses in this manuscript used Wald ratios as described in the methods section due to the general availability of only one cis pQTL per protein after clumping at $r2 < 0.01$. No inverse-variance weighted models were performed and we have now updated the methods section to reflect this.

9. *The MR section should include estimates from MR-Egger and the weighted median (WM) to account for directional pleiotropy. Bi-directional MR could also help evaluate potential reverse causality.*

   Response: Thank you for this suggestion. Due to the availability of only a single cis-pQTL instrument for most proteins, we performed MR analyses using the Wald ratio method. MR-Egger and weighted median approaches require multiple independent instruments per exposure to provide valid estimates, which was not feasible in our analysis. We have now conducted bi-directional MR and added the corresponding methods (**page 10, lines 341:348**) and results (**page 13, lines 452-455**) and in **Supplementary Table S12**.

10. *The robustness of the colocalization analysis to prior assumptions should be discussed. Were alternative priors tested? This is particularly important given the complexity of cis-pQTL regions.*

    Response: We assessed the sensitivity of our colocalization results to the choice of prior using the in-built functions as part of the coloc package. Choice of prior (p12 ranging from 1e-4 to 1e-6) was not observed to materially alter the conclusions of these tests in our data. A sentence has been added to Methods on **page 10, lines 336-339:**

    > "Within each predefined genetic region (250 kb around the index cis-pQTL), we tested for the presence of a single shared causal variant between protein and cancer using conventional colocalisation, as well as multiple shared causal variants using the Sum of Single Effects (coloc SuSiE) regression method, which can account for the presence of multiple causal variants of which only a subset are shared between two traits. Default priors were used for analyses and PP4 > 0.7 was accepted as evidence for colocalisation; we also assessed the sensitivity of our colocalization results to the choice of priors (with p12 ranging from 1e-4 to 1e-6)."

    and to the Results on **page 13, lines 448-450**:

Colocalisation analyses showed support for a shared causal variant between SPINT2 (OR:2.11, 95%CI:1.70-2.63, PP4: 97%) and NME3 (1.25, 1.11-1.41, PP4: 71%) and these conclusions were generally robust to the choice of priors (i.e. SPINT2 colocalised when p12= 1e-4 to 1e-6, while NME3 colocalised when p12=1e-4 to 1e-5).

11. *The authors employed MR to infer causality. However, given the complexity of cis-pQTL regions, further elaboration on the methodology used for colocalization analysis is needed. Specifically, did the coloc-SuSiE model account for multiple causal variants, and if so, how was this implemented?*

Response: Our colocalization analyses employed both naïve colocalisation and also the R **coloc** package's coloc.susie() function, which first fine-maps each trait with SuSiE (allowing up to 10 independent signals per locus using the local LD matrix) and then integrates those "single-effect" credible sets to compute Bayes factors and posterior probabilities of shared causality. In doing so, this method is explicitly colocalise and testing for multiple causal variants rather than assuming a single effect. Further details to this effect have now been added to the manuscript on **page 10, lines 336-339**.

"Within each predefined genetic region (250 kb around the index cis-pQTL), we tested for the presence of a single shared causal variant between protein and cancer using conventional colocalisation, as well as multiple shared causal variants using the Sum of Single Effects (coloc SuSiE) regression method, which can account for the presence of multiple causal variants of which only a subset are shared between two traits. [24] Default priors were used for analyses and PP4 > 0.7 was accepted as evidence for colocalisation; we also assessed the sensitivity of our colocalization results to the choice of priors (with p12 ranging from 1e-4 to 1e-6)."

12. *The time-stratified analysis is a strength. However, the authors should elaborate on the biological mechanisms that might explain the stronger associations seen in samples taken more than 7 years before diagnosis.*

Response: We thank the reviewer for this helpful suggestion. The stronger associations in blood collected more than seven years before diagnosis are consistent with potential endogenous processes/factors acting early in prostate cancer aetiology rather than markers of subclinical disease. Prostate cancer often evolves slowly and typically becomes immunologically cold, with limited immune infiltration in established tumours. Circulating immune proteins measured well before diagnosis may therefore reflect host immunosurveillance and tissue homeostasis at a time when these processes are intact, rather than reflecting inflammatory responses to an existing lesion. For example, higher IL-15, a cytokine that supports NK- and CD8-cell function, was more clearly associated with lower prostate cancer risk in samples taken more than seven years before diagnosis. This temporal pattern reduces the likelihood of reverse causation in determining our findings and instead supports an interpretation that aspects of systemic immune competence influence risk during earlier stages of tumour development. In the Discussion (**page 14-15, lines 494-500**), we have explained this rationale with the findings being compatible with an aetiologically relevant window long before diagnosis.

"Our findings are consistent with potential endogenous processes/factors acting early in prostate cancer aetiology rather than the associations reflecting markers of subclinical disease. Prostate cancer often evolves slowly and typically becomes immunologically cold, with low tumour infiltrating lymphocytes, such as active T cell and NK cells, in tumour tissue compared to tissue from healthy prostates.[3,25] Circulating immune proteins measured well before diagnosis may therefore reflect host immunosurveillance and tissue homeostasis at a time when these processes are intact, rather than reflecting inflammatory responses to an existing lesion."

13. *Most genetic analyses were based on individuals of European ancestry. While this limitation is acknowledged, the authors should provide a more detailed discussion of how this affects the generalizability of their findings, particularly in the context of multi-ethnic populations.*

Response: We thank the reviewer for raising this important point. As mentioned in the discussion, the majority of our genetic analyses were conducted in individuals of European ancestry. Nonetheless, we were able to replicate our key finding for PARP1 and prostate cancer risk in the Multiethnic Cohort (MEC), which included participants from several non-European populations (White: 555 cases; Latin American: 602; Japanese: 1,006; Black: 538; Hawaiian: 182). This suggests that, at least for this locus, the signal observed in the UK Biobank is consistent across ancestries. However, we also note that the number of non-European cases remains relatively limited, and only a few large-scale datasets to date have directly measured plasma protein levels in diverse populations. Expanding such resources will be an important priority for future research. We discuss replication of PARP1 in the MEC in the Discussion on **page 16, lines 553-556**:

"We also identified genetically predicted higher PARP1 to be associated with an increased risk of PC overall using exome protein score analyses and were successful in replicating this association in an external, diverse prospective cohort consisting of African American, European American, Japanese American, Latino, and Native Hawaiian men."

and **page 16, lines 567-572**:

"However, we note that the data in this analysis were majority participants in EPIC that are predominantly of European ancestry and that almost all cases in our UK Biobank analyses are also of European ancestry; the promise of these initial proteomics results further underscores the need for the prompt diversification of studies on proteins and cancer and the assessment of the generalisability of findings across populations with differing ancestries."

14. *The study analyzes 368 proteins, representing only a fraction of the immune proteome. Discussing potential proteins of interest for future research would be beneficial.*

Response: We agree that 368 proteins represent only a portion of the immune proteome. We selected the Olink Explore Inflammation I panel as a first investigation into many common and notable bioamrkers of the immune system, while recognising that it does not capture the full breadth of immune signalling or cell type specific markers.

With large-scale high-throughput proteomics becoming available in population cohorts, including Olink HT and emerging Illumina protein-prep workflows both in the UK Biobank, it will be possible to deepen coverage across pathways such as soluble checkpoint signalling, innate sensing, myeloid and NK surveillance, complement, and stromal-immune crosstalk.

At present, however, our study provides early evidence that prospective measures of the circulating immune system carry information relevant to prostate cancer aetiology. Expanding coverage in future work may help refine mechanistic hypotheses and nominate additional biomarkers or preventive targets.

We addressed this limitation in the discussion on **page 17, lines 589-591** and as below:

> "Further, we investigated a sizable but nonetheless still modest subset of all immune and inflammation-related proteins. Therefore, the seven proteins that we identify in this study likely represent only a fraction of proteins with an important role in PC aetiology. With large-scale high-throughput proteomics increasingly available in population cohorts (e.g. Olink HT and emerging Illumina protein-prep workflows in UK Biobank), it will be possible to broaden coverage beyond the panel we studied. Deeper characterisation of the proteome in larger sample sizes from mature prospective cohorts in the future will be needed to bring greater resolution to the network of the PC risk proteome - in particular, proteins that might be linked to a positive feedback loop for IL-15 involving NK cells, DC and T lymphocytes."

15. *The identification of PARP1 as a risk protein is noteworthy, but additional functional validation (e.g., in vitro or in vivo studies) would strengthen the evidence base.*

Response: We agree with the reviewer that functional validation is an important step in confirming the causal role of the identified proteins. While in vitro or in vivo studies were beyond the scope of this epidemiological investigation, we have added the following sentence to the Discussion (**page 16, lines 562-564**):

> "Further investigation, including functional validation, is needed to elucidate its mechanistic role in early-stage disease."

Minor Comments

16. *Figure 1: Ensure consistency in the reported number of proteins (386 vs. 368). Cross-check with the supplementary tables and correct any discrepancies.*

Response: Thank you, we have checked these numbers.

17. *Figure 5: Include sample sizes for each cohort in the forest plots for PARP1 to provide better context.*

Response: We have revised the graph and added in the number of cases.

18. *The leave-one-out plots for MR analysis need to be redrawn for improved legibility.*

Response: Thank you for your comment. We did not perform leave-one-out analyses for MR in this study. Could you please clarify if this comment refers to another plot in the manuscript that requires improvement? We would be happy to revise accordingly.

19. *Expand citations related to immunosurveillance and dendritic cell roles in PC, including recent advances in checkpoint inhibitors.*

Response: We thank the reviewer for this helpful suggestion. We have expanded the Discussion with additional citations on immunosurveillance (**page 14, line 498**), the role of dendritic cells in prostate cancer, and recent advances in immune checkpoint inhibitors on **page 14-15, lines 494-500**:

"Our findings are consistent with potential endogenous processes/factors acting early in prostate cancer aetiology rather than the associations reflecting markers of subclinical disease. Prostate cancer often evolves slowly and typically becomes immunologically cold, with low tumour infiltrating lymphocytes, such as active T cell and NK cells, in tumour tissue compared to tissue from healthy prostates.[3,25] Circulating immune proteins measured well before diagnosis may therefore reflect host immunosurveillance and tissue homeostasis at a time when these processes are intact, rather than reflecting inflammatory responses to an existing lesion."

and also on **page 15, lines 523-530**:

"We have previously reported an inverse association between FLT3LG and PC risk using both observational and using exome analyses.[6] Here we additionally demonstrate, as with IL15, its strong inverse association with risk in men who developed PC longer after having given blood. FLT3LG is primarily produced by NK cells and controls the development of dendritic cells via binding to FLT3 to increase the presentation of tumour antigens, as part of immunosurveillance that enhances T cell and NK cell activity.[33-35] In addition, FLT3LG is linked to higher levels of stimulatory dendritic cells within the tumour environment,[36] playing a pivotal role in enhancing the effectiveness of immune responses against cancer, including the improvement of outcomes in melanoma patients undergoing anti-PD-1 immunotherapy. Recent reviews further highlight the importance of dendritic cell priming in prostate cancer immunobiology and as a determinant of responsiveness to immunotherapy.[37] Clinical studies also indicate that checkpoint inhibitors have limited overall benefit in unselected prostate cancer but can elicit durable responses in biomarker-defined subsets.[38]"

Reviewer #2 (Remarks to the Author):

The authors conducted large-scale observational studies and genetic analyses to evaluate the association between inflammation- and immune-related proteins and prostate cancer (PC) risk. This study identified both measured and genetically predicted levels of several proteins associated with PC risk. While the findings are of some merit and the study design is fundamentally sound, the following comments and suggestions may help the authors enhance their manuscript.

Major comments:

1. ***Method:*** *It is possible that multiple variants flanking a gene region affect the gene transcription and translation levels, which may be highly correlated with each other and be pleiotropic for multiple traits. The Wald ratios and IVW MR methods used in this study cannot address this issue. Have the authors tried other MR approaches such as cisMR-cML for this assumption?*

   Response: We agree that multiple correlated variants within cis-regions can influence gene transcription and translation, potentially introducing pleiotropy. In our analyses, most proteins had only a single independent cis-pQTL after clumping at $r^2 < 0.01$, which precluded the use of multi-instrument approaches such as MR-Egger, weighted-median MR, or cisMR-cML. Instead, we applied complementary strategies to address this assumption: (1) colocalization analyses using the coloc and coloc-SuSiE frameworks, which explicitly model multiple causal variants while accounting for local LD structure (**page 10, lines:333-339**); and (2) reverse MR analyses to evaluate potential reverse causation (**page 10, lines:341-348**). We have also revised the Discussion (**page 17, lines 575–583**) to acknowledge this limitation and note that residual pleiotropy within cis-regions cannot be entirely excluded.

2. ***Method:*** *May consider reverse MR, to estimate the effect of PC liability on levels of interesting proteins.*

   Response: Thank you. We have now conducted bi-directional MR and added the corresponding methods (**page 10, lines 341:348**) and results (**page 13, lines 452-455**) and in **Supplementary Table S12** (see also our response to **Reviewer 1**, **Comment 9**).

3. ***Method:*** *pQTL and ExGS were conducted using UK Biobank protein data and exome sequencing data, however, it is unknown which genotyping platform was used for the MEC cohort. Was MEGA-SNP array or exomechip used? If so, which SNVs were used in generating ExGS for selected proteins, especially PARP1, in UKB and were same SNVs used in MEC? How are the qualities of these SNVs in MEC? A large proportion of rare variants (MAF<0.005) had bad genotype clusters of low quality from SNP arrays or exomechips.*

   Response: We thank the reviewer for this important question regarding the MEC data used for PARP1. We have added a citation describing the MEC genetic protocol (**page 9, line 308**).

The MEC cohort was genotyped primarily using Illumina SNP arrays, with most remaining samples genotyped in 2020–2021 on the Illumina Global Diversity Array (GDA, including MEGA content); no exome sequencing or exome chips were used in MEC.

The PARP1 Exome Genetic Score (ExGS) was derived in UK Biobank using exome sequencing data. For MEC, the PARP1 ExGS was constructed using the same PARP1 SNVs from the UKBB-derived instrument and the scoring allele effect directions of increasing PARP1 levels. All PARP1 SNVs were common variants (MAF >1%).

Regarding variant quality, MEC genotypes underwent extensive QC followed by ancestry-specific imputation using the 1000 Genomes Project and TOPMed reference panels. For MEC, variants with MAF >1% have high imputation quality across racial and ethnic groups (mean $R^2 \geq 0.8$), and no PARP1 SNVs included in the ExGS were poorly imputed. Rare variants (MAF <0.5%), which are known to have unreliable clustering on SNP arrays, were not included in the PARP1 ExGS in MEC.

Additionally, these details and a table describing the SNVs included in the PARP1 panel have been added to **Supplementary Materials S5**.

4. *Results from Mendelian randomization analyses: It would be nice if the authors list the variance explained by the instrumental cis variant(s) used in MR. And acknowledge the limitations of IV(s) selected of cis-pQTLs in Discussion.*

   Response: We agree that instrument strength is an important consideration in Mendelian randomization analyses. We therefore report the F-statistics for each cis-pQTL used as an instrument in **Supplementary Table S9**, indicating a limited risk of weak-instrument bias. For completeness, we have also added the proportion of variance explained by each cis-pQTL to this table. The limitations associated with using cis-pQTL instruments are acknowledged in the Discussion (**page 17, lines 575–583**).

5. *Results from Mendelian randomization analyses: cisMR support a few proteins for PC risk, which may reflect limited variance explained by selected cis-pQTLs. Please add effective allele of each cis-pQTL as well as EAF in exposure and outcome data sets. The section title seems confusing, should change to "MR analyses and colocalization". Colocalization is not part of MR but provides complementary evidence for a shared genetic component for the tested protein and PC. The later cannot determine association direction, in this case.*

   Response: We have revised the section title to "Mendelian randomisation and colocalisation analyses" to clarify that colocalization is presented as complementary evidence rather than as part of the MR framework. In addition, we have added the effect allele and effect allele frequency (EAF) for both exposure and outcome for each cis-pQTL used in the MR analyses to **Supplementary Table S9**, to improve transparency and reproducibility.

6. *Results in Fig. 5 and Table S8: I would suggest results of meta-analysis of UKBB and MEC: White be included in Part A. Details for used SNVs, MAF and quality, should be described.*

Response: We thank the reviewer for this helpful suggestion. We have now included the meta-analysis combining UK Biobank (UKBB) and the White (European American) subset of the Multiethnic Cohort (MEC) in Figure 5A, allowing an ancestry-matched comparison.

All genetic panels used in this study are publicly available on GitHub[1] (e.g. the PARP1 panel), including the final panel definition, a summary of SNV retention at each development step, and logs detailing SNV selection and linkage disequilibrium handling.

Population-level allele frequency information primarily follows Dhindsa et al. We have added **Supplementary Materials S5** to provide more details for PARP1 that lists, for each SNV, the minor allele frequency, gene annotation, and the most damaging predicted functional consequence. Panel-specific details are also provided.

All exome-based scores were derived from rigorously quality-controlled data. SNVs passed both the UK Biobank Pharma Proteomics Project QC and the standard UKBB exome sequencing QC pipeline, with additional internal filters excluding individuals and variants with >1% missingness. For MEC, replication relied on common variants imputed using array-based data; as reported in the MEC preprint, variants with MAF >1% showed high imputation quality (mean R^2 ≥0.8 across ancestries), and no SNVs in the PARP1 panel were poorly imputed. We have added a citation describing the MEC genetic protocol (**page 9, line 308**).

7. ***Discussion:*** *Limitations of the MR analysis used in this study (such as IVs and methods) need to be discussed. In addition, cis-pQTL may explain a limited proportion of variance of the nearby protein. Genetically determined protein levels by variants across the genome (eg, from GWAS on each protein) may greatly improve the statistical power in the MR analysis.*

Response: We agree that cis-pQTL typically explain a limited proportion of variance in circulating protein levels, which may reduce statistical power and lead to imprecise MR estimates. However, we note that all cis-pQTL instruments used in this study had F-statistics (**Supplementary Table S9**) well above the conventional threshold of 10, suggesting that weak instrument bias is unlikely to be a major concern in our analyses.

While genetically predicted protein levels using variants across the genome could increase explained variance, we deliberately focused on cis-pQTL instruments to minimize horizontal pleiotropy. For protein traits, trans-acting variants often influence protein levels via upstream regulators or shared biological pathways, which may violate the exclusion restriction and complicate causal interpretation. The cis-MR framework therefore represents a trade-off between statistical power and biological specificity.

We have revised the Discussion (**page 17, line 575-583**) to clarify these limitations and to emphasize that null or modest associations should be interpreted in the context of both the variance explained by cis instruments and their demonstrated strength based on F-statistics:

[1] https://github.com/Cancer-epi-unit/INF_PC

"Although cis-pQTL typically explain a modest proportion of variance in protein levels, all instruments used in our MR analyses had F-statistics above 10, suggesting adequate instrument strength. Nevertheless, for some proteins the variance explained was very low which limited our ability to detect modest effects. Furthermore, most proteins were instrumented by a single independent cis-pQTL, which precluded the application of multi-instrument MR methods designed to account for horizontal pleiotropy, and residual pleiotropy within cis-regions cannot be fully excluded. Limited or no support from MR and colocalisation analyses may also be due to other factors such as case mix in the cancer GWAS and genomic complexity of cis-protein regions, which remain challenging for colocalisation methods to resolve."

8. ***Discussion:*** *The discussion on replicated findings for the FLT3LG protein seems not adding more merit to this study.*

   Response: We agree that FLT3LG has been reported previously, including in our earlier work (Papier et al., 2024). Nonetheless, we consider it appropriate to retain FLT3LG in the Discussion, as its inclusion strengthens and extends the existing evidence rather than presenting it as a novel finding. Using data from EPIC, we provide an independent replication of this association, and the concordance of FLT3LG with other immune-related proteins identified in this study adds biological coherence to the emerging immunosurveillance signal.

9. ***Discussion:*** *More discussions on the possible connection between discovered proteins and immunosurveillance in PC are needed.*

   Response: We thank the reviewer for this valuable suggestion. We have expanded the Discussion (**page 14-15, lines 494:500**) to provide a more detailed interpretation of how the identified proteins relate to immunosurveillance and prostate-cancer aetiology. In particular, we highlight that IL15 and FLT3LG are key regulators of dendritic-cell and natural killer (NK)–cell activation, both of which are central to early tumour immune recognition (**page 15, lines 503-505**, and **page 15, lines 523-530**, respectively).

Minor comments:

10. *Typos and spelling errors, e.g., caption letter in "Nucleotide" in line 239, "mean" for "men" in line 243, "We note, however, we note" in line 539,…*

    Response: Thank you for noting these typos. We have corrected all identified errors and proofread the manuscript for consistency.

11. *FLT3LG has been reported as a risk protein for PC by the same group (ref 4) and should not be presented as an important finding.*

    Response: We have addressed this point in an earlier comment (**Reviewer 2**, **Comment 8**).

12. Lines 223-224: In previous study, EA measured protein values were log-base-2 transformed as normalized protein expression (NPX), which then subjected to inverse rank normal transformation. Can the authors clarify why they mean centered and standardized the NPX values instead?

Response: In our earlier work, inverse rank normalisation was used to improve the normality of protein distributions. In the present study, however, based on experience comparing results across proteomic platforms and before and after rank-based transformation, we observed that inverse rank normalisation can affect estimates for a small number of proteins. We therefore chose to mean-centre and standardise NPX values, which are already log2-transformed by design, in order to preserve relative differences and avoid unnecessary loss of information. We do not expect major differences between approaches, as both rely on the same underlying measurements and scaling, with the only distinction being the final step that enforces a normal distribution in the previous publication.

13. Reference 14 in line 284 overlaps with reference 4.

Response: Thank you for noting this. We have corrected the duplication in the reference list.

14. Line 309: LD clumping cutoff value r2<0.05, a typo for 0.01?

Response: We have revised it in the text (**page 9, line 291**)

15. Lines 354-355, it would be nice to indicate the version of the coloc package

Response: We have added the version of the coloc package to the Methods section, (**page10, line 352**).

16. Method: Mendelian randomization analyses: This section may be "Mendelian randomization and colocalization analyses".

Response: We have revised the section title to "Mendelian randomization and colocalization analyses", as suggested.

17. Results of "Exome analyses" in Table S7, many SNVs (ID) with discordant directions between OR and Z_STAT, please correct them.

Response: We have updated the exome analysis results in Table S7 and corrected the inconsistencies between the directions of the odds ratios and the corresponding Z-statistics. The revised table now reports internally consistent effect directions throughout.

18. Discussion: line 481, lines 516 and 517, and line566, where are the results for protein and PC association for cases diagnosed 10 years/a decade after blood draw?

Response: We revised the text from more than ten to more than seven years for clarity. The rationale for the original wording was that the median follow-up time was 12.2 years, meaning that

a substantial proportion of cases were diagnosed at least a decade after the blood draw. We agree though that this could be confusing, and we have revised the text to ensure that the long-lag definition is described consistently throughout the manuscript.

19. Discussion: line 489, reference for "IL15 knockout mice also lack NK cells" is missing.

Response: Thank you for this comment. We have added the reference to the revised manuscript (**page 15, line 511**).

20. Discussion: lines 526-536, no references to support the hypothesis and views for the role of PARP1 in PC?

Response: We have added two references (**page 16, line 562**) to support the role of PARP1 in prostate cancer

Reviewer #3 (Remarks to the Author):

This study investigated the association between 368 inflammation and immune-related proteins and prostate cancer (PC) risk using observational data from EPIC and UKB. The authors further explored causality using exome-wide genetic scores, Mendelian Randomization (MR), and colocalization. The key findings suggest associations between several proteins and PCs risk based on observational analysis, and a positive association of PARP1 with PCs risk based on exome analysis. Additionally, SPINT2 and NME3 were suggested to play a role in PCs risk based on MR analysis.

Originality and significance:

1. *This study is valuable as it integrates large-scale proteomic and genetic datasets to explore the role of immune-related proteins in PC risk. This is an important area of research, as the study has the potential to contribute to our understand of PC immunology. However, to strengthen its impact, the authors may consider highlighting the novelty of their approach more explicitly. Previous studies have investigated proteomic signatures for PC using proteome-wide analyses rather than focusing on specific panels (e.g. Lin C et al 2024, medrxiv; Cheng L et al 2024, Discov Oncol; Sun W et al 2024, BMC Cancer). A clearer discussion of how this study advances beyond prior work would help clarify its unique contributions.*

   Lin C et al, Causal associations between plasma proteins and prostate cancer: a Proteome-Wide Mendelian Randomization, medRxiv 2024.09.17.24312688; doi: https://doi.org/10.1101/2024.09.17.24312688
   Cheng L, et al. Evaluation of circulating plasma proteins in prostate cancer using mendelian randomization. Discov Oncol. 2024 Sep 17;15(1):453. doi: 10.1007/s12672-024-01331-3
   Sun W et al. Associations between genetically predicted concentrations of plasma proteins and the risk of prostate cancer. BMC Cancer. 2024 Jul 27;24(1):905. doi: 10.1186/s12885-024-12659-y.

   Response: As noted in the introduction, there are limited prospective observational data on the majority of inflammation and immune-related proteins in relation to prostate cancer. The studies referenced by the reviewer, while valuable and scientifically important, are primarily based on Mendelian randomization analyses, which use genetic proxies (typically cis-pQTLs) to estimate the effects of protein levels, rather than relying on direct measurements.

   While MR is a powerful method for causal inference and can help overcome key limitations of observational studies, particularly by reducing confounding and reverse causation, it also has some limitations. Cis-pQTLs are not available for all proteins, and where they do exist, the strength and specificity of these instruments can vary, limiting the robustness of MR analyses. Genetic instruments may reflect specific isoforms or proteoforms, which do not always correspond to total circulating protein concentrations measured in plasma. MR estimates reflect lifelong genetic predisposition to higher or lower protein levels and may not capture the dynamic or time-dependent changes that are particularly relevant to disease initiation and progression. MR estimates may be affected by complex LD or pleiotropy unless addressed with colocalisation and sensitivity analyses.

   These limitations underscore the value of complementing MR with direct protein measurements in prospective studies, which allow for time-stratified analyses (e.g., by lag time) and may offer an additionally temporally relevant understanding of protein–disease associations. As mentioned in the introduction, the main novelty of the current study lies in its integration of prospective proteomic data with genetic evidence across multiple cohorts, enabling a triangulated analytical

framework. This approach improves biological interpretability, strengthens causal inference, and supports the prioritisation of candidate immune-related proteins for future investigation.

Additionally, the introduction could better establish the biological rationale by explaining how inflammation contributes to prostate carcinogenesis. The epidemiological claims about CRP and IL6 should be expanded with further discussion and additional citations to support their proposed role in PC.

Response: As we have described in the introduction, there are relatively few prospective observational data on PC in relation to the majority of inflammation and immune related proteins. The majority of studies investigated the association with CRP and results from meta-analyses are inconsistent. We revised the Introduction and added more details about these studies on **page 3-4, lines 106-115**.

The role of inflammation and the immune system in cancer aetiology is complex. While chronic inflammation is a hallmark of cancer and can promote cellular proliferation, immune and inflammation-related signalling can also detect and eliminate abnormal cells, preventing tumorigenesis via immunosurveillance.[1-3] To date for prostate cancer (PC), there is suggestive epidemiological evidence for an association with a limited number of relevant candidate proteins, such as C-reactive protein and Interleukin (IL) 6[4]. A systematic review and meta-analysis of inflammatory markers and cancer risk, which included up to seventeen studies of prostate cancer, found CRP to be the most frequently examined marker; although most studies reported null findings, pooled prospective estimates revealed a weak but statistically significant association between elevated CRP and PC risk. IL6 showed no consistent effect, with one study reporting a negative association and three studies reporting null findings.[4] More recently, a prospective analysis from the Osteoporotic Fractures in Men (MrOS) cohort reported that moderate or high IL10 levels were associated with substantially lower PC risk, with no significant associations for IL6, CRP, TNF-α, or overall inflammatory burden.[5] Despite these insights, prospective observational data on most immune and inflammation-related proteins in PC remain scarce.[6]

Beyond these, some methodological, statistical, and interpretation concerns need to be addressed to ensure the robustness of its conclusions. Below are some suggestions to improve the clarity, transparency, and the reliability of the results.

Data and methodology:

2. *This study includes only Germany, Italy, the Netherlands, Spain, and the UK study centres from EPIC cohort without providing a clear justification for excluding other EPIC study centers. If these exclusions were due to data availability or quality control, it would be useful to state this explicitly. Otherwise, it raises concerns about potential selection bias, as excluded centers may have different participant characteristics or follow-up durations.*

   Response: Thank you for your comment. While the full EPIC cohort includes participants from 10 countries, our analysis was limited to male participants from five countries (Germany, Italy, the Netherlands, Spain, and the UK) that contributed to the prostate cancer nested case-control studies and had available proteomic data for this analysis. This is mentioned in the method section (**page 4, lines 139-140**):

   > "The current study included male participants who had blood samples from centres located in Germany, Italy, the Netherlands, Spain and the United Kingdom."

3. *The exome analysis harmonized the genetic variants by orienting them to the protein-increasing alleles in exome analysis. However, some technical details remain unclear: (1) how were ambiguous variants (with A/T or C/G alleles) handled?, (2) were trans-pQTLs included in the exome-wide genetic score calculation?, and (3) how were pQTLs weighted in the genetic score calculations?*

   Response: Regarding (1), since this study is based on sequencing data from the same cohort as was used in Dhindsa et al, which were processed by the same pipeline and against the same reference genome, issues with ambiguous variants (e.g., A/T or C/G SNPs) are not a concern for our analyses. This is in contrast to genotype array data, where ambiguous SNPs and strand mismatches are a common source of harmonization errors.

   (2) Yes, trans-pQTLs were included in the exome-wide genetic score (exGS) calculation, provided they reached genome-wide significance ($P < 5\times10^{-8}$).

   (3) The weights used for constructing the exGS were the beta estimates reported in Dhindsa et al.[1], which were derived from pQTL effect sizes.

   Dhindsa, RS., et al. (2023). "Rare variant associations with plasma protein levels in the UK Biobank". Nature 622(7982): 339-47.

4. *MR analysis was used to assess causality but requires additional sensitivity analyses to ensure the robustness of results. Some key issues/missing details include: (1) how many of the 368 proteins have cis-pQTLs for MR analysis? (2) MR analysis in the MHC region e.g. CDSN, HLA-DRA, and HSPA1A is challenging due to extensive linkage disequilibrium. (3) How did the authors ensure that independent SNPs were used for MR and exome-wide genetic score analyses?, and (4) no discussion of horizontal pleiotropy, a major limitation in MR studies. It would be beneficial to include MR-Egger, MR-PRESSO, or weighted median sensitivity analyes to assess pleiotropy*

   Response: (1) As noted in the manuscript on **page 13, lines 438-439** we conducted MR analyses for prostate cancer on the 285 out of the 368 proteins that had available cis-pQTLs.

   > "We also conducted MR analyses for PC for the 285 (out of 368) proteins in our study that had available *cis*-pQTLs."

   (2) We agree that inference for cis pQTL within the MHC can be challenging and that no easily implementable solution is currently available. It is for this reason that while we report results for protein MR analyses where cis pQTL are located within the MHC, no colocalization has been conducted due to uncertainty in the methods' ability to resolve complex LD structures in this locus. This is discussed in the results section on **page 13, lines 442-445**:

   > "Three of these ten proteins, CDSN, HLA-DRA, and HSPA1A, were located within the MHC. While MR results for these proteins are reported for completeness, due to the complexity of LD and the sensitivity of colocalisation methods to LD references, we chose not to conduct colocalization analyses for cis-pQTL MR results located in the MHC region."

   (3) To ensure that only independent SNPs were included across the two analyses, the following approach was applied:

   For the exSC analysis, an LD matrix was generated using the entire European subset of the UK Biobank. Variants were selected if they were not correlated ($r^2 < 0.05$) within a 10 Mb sliding window, based on pairwise comparisons of the absolute Z-score (β/SE). The process was iteratively

repeated until the highest Z-scoring independent variants were retained, as described in our previous work by *Papier et al*.

For the cis-MR analysis, an LD matrix was generated using common SNPs from 20,000 randomly selected UK Biobank participants. Independent SNPs were defined as those with $r^2 < 0.01$ within a 10 Mb window.

Papier, K., et al. (2024). "Identifying proteomic risk factors for cancer using prospective and exome analyses of 1463 circulating proteins and risk of 19 cancers in the UK Biobank." Nature communications 15(1): 4010.

Additionally, an extra column titled "r^2 with cis-panel exSC SNPs" was added to **Supplementary Table S9** to indicate whether significant MR findings were in LD with SNPs selected in the cis-exSC analysis, for greater transparency. This LD assessment was performed using the LDlink tool with the high-coverage 1000 Genomes European reference panel.

(4) Horizontal pleiotropy is the concern to causal inference in MR. In the context of single-variant cis pQTLs MR analyses, methods such as MR-Egger, weighted median, and MR-PRESSO are not applicable because they require multiple independent instruments. We therefore used approaches suited to our design: colocalisation with SuSiE to support a shared causal variant, and Steiger filtering via reverse MR to identify potential reverse causation. Nonetheless, cis instruments are closer to the relevant biology and are therefore less susceptible to pleiotropy than trans or genome-wide instruments but not required, of course, to be immune. We have therefore revised the Discussion to acknowledge this limitation (**page 17, lines 578-581**).

> "Furthermore, most proteins were instrumented by a single independent cis-pQTL, which precluded the application of multi-instrument MR methods designed to account for horizontal pleiotropy, and residual pleiotropy within cis-regions cannot be fully excluded."

Statistical issues:

5. *The study reported differences in baseline characteristics between cases and controls (Table 1 and Supplementary Table 3) but does not report formal statistical tests. Without p values, it is unclear whether these differences are statistically significant.*

   Response: As recommended by the STROBE guidelines, we did not include p-values in **Table 1** because the purpose of baseline characteristics is to describe the study population rather than to make formal comparisons between cases and controls. Statistical testing of baseline characteristics in observational studies can be misleading, as any observed differences are a function of study design (e.g., matching or stratification) rather than random assignment. Instead, we present the actual values to allow readers to assess the clinical or epidemiological relevance of any differences.

6. *The study meta-analyzed hazard ratio (HR) from UKB and odds ratio (OR) from EPIC to estimate relative risk (RR) using inverse-variance weighted meta-analysis. However, HRs and ORs are not directly comparable, and thus this approach raises concerns about statistical validity. The authors could consider either estimating ORs from UKB and meta-analyzing them with EPIC results, or analyzing HRs from UKB and ORs from EPIC separately, treating one as a replicating cohort instead of meta-analyzing them together.*

   Response: While ORs from traditional case-control studies are not directly comparable to HRs, in nested case-control designs they can be interpreted as HRs under the proportional hazards

assumption[2]. We addressed this in the manuscript and cited the relevant reference on **page 8, lines 271-273**, stating: "Since cases and controls are matched on follow-up time in the nested case-control study design, the odds ratio can be interpreted as the hazard ratio, assuming the proportional hazards assumption holds." The theoretical basis is outlined in Section 5.4.1 of the cited reference and mentioned below:

"To summarize, table below represent the expected number of cases and controls by exposure group based on data generated by risk set sampling. At any time t, let $N_E(t)$ and $N_{\bar{E}}(t)$ be the number of individuals still at risk in the exposed and unexposed population, respectively; the number of cases expected in the exposed group at time t is then $N_E(t) \times h_E(t)$, by the definition of the hazard rate, $h_E(t)$, under exposure. Similarly, the number of cases expected in the unexposed group is $N_{\bar{E}}(t) \times h_{\bar{E}}(t)$. If m controls are sampled at time t, then the expected number of exposed and unexposed controls are simply $m \times \frac{N_E(t)}{N_E(t)+N_{\bar{E}}(t)}$ and $m \times \frac{N_{\bar{E}}(t)}{N_E(t)+N_{\bar{E}}(t)}$, respectively.

Table 1 Expected cell entries in a 2×2 contingency table for data generated by risk set sampling

		Case	Risk-set control
Exposure	E	$N_E(t) \times h_E(t)$	$m \times \dfrac{N_E(t)}{N_E(t) + N_{\bar{E}}(t)}$
	Not E	$N_{\bar{E}}(t) \times h_{\bar{E}}(t)$	$m \times \dfrac{N_{\bar{E}}(t)}{N_E(t) + N_{\bar{E}}(t)}$

Based on these expected numbers, the corresponding odds ratio (OR) is calculated as follows:

$$OR = \frac{ad}{bc} = \frac{[N_E(t) \times h_E(t)] \times \left[m \times \dfrac{N_{\bar{E}}(t)}{N_E(t) + N_{\bar{E}}(t)}\right]}{[N_{\bar{E}}(t) \times h_{\bar{E}}(t)] \times \left[m \times \dfrac{N_E(t)}{N_E(t) + N_{\bar{E}}(t)}\right]} = \frac{h_E(t)}{h_{\bar{E}}(t)} = RH(t)$$

Assuming proportional hazards, the relative hazard RH(t) is constant over time, and the odds ratio derived at each time point approximates this constant hazard ratio."

[2] Jewell NP. Study designs. Statistics for Epidemiology. New York: Chapman and Hall/CRC; 2004.

7. *Several results are described in the result section without explicitly reporting ORs, HRs, 95% confidence intervals, or p-values. This makes it difficult to assess the statistical robustness.*

Response: Thank you for your comment. We have revised the manuscript to include ORs and 95% confidence intervals in the Results (**page 12, lines 393-395**):

"In EPIC, no proteins were associated with high grade, advanced stage, or aggressive PC after accounting for multiple testing. However, we identified inverse associations at conventional significance for IL15 (0.74, 0.60-0.91) and FLT3LG (0.75, 0.61-0.92) with advanced stage and BCL2L11 (0.82, 0.70-0.97), PGF (0.83, 0.71-0.98), and FLT3LG (0.85, 0.74-0.99) with aggressive PC subtypes (Error! Reference source not found.)."

and on **page 13, line 460**:

"Concordant with observations from our observational analyses from meta-analyses in EPIC and UKBB, we found evidence at conventional significance in exome protein score analyses for inverse

associations of FLT3LG (OR:0.97, 95%CI:0.95-0.99) and PGF (OR:0.96, 95%CI:0.94-0.98) with PC risk (**Error! Reference source not found.**).”

and added references to the relevant **Supplementary Materials** (e.g., **Sections S4.1** and **S4.2**), particularly for the cross-cancer analysis.

8. *The study applies multiple testing corrections (ENT and Bonferroni correction), but the results are not consistently presented in tables or results section. It is also unclear what significance threshold was used for both multiple testing corrections.*

   Response: Thank you for your comment. We used the Effective Number of Tests (ENT) method to adjust for multiple testing in all observational analyses, including EPIC, UK Biobank (UKBB), the meta-analysis, and subgroup analyses. This is mentioned in both the abstract (“…the effective number of tests (ENT) was used to control for multiple testing”) and the Methods section (“We considered any association with a p-value below 0.05 divided by the effective number of tests (ENT) as statistically significant”). In the revised version, we have added a dedicated section titled "Multiple Testing Correction in Observational Studies” (**page 8, lines 279-285**) to clearly describe the approach and threshold used (0.05/189).

   For the MR analyses, we applied Bonferroni correction and this is described in the text on **page 10, lines 331**:

   > “To account for multiple testing in our MR analyses, a Bonferroni-corrected threshold of 0.05/285 was used based on the number of proteins with available cis-instruments (i.e. 285 proteins)”

   For the Exome Analysis, **page 9, lines 298-301**:

   > “For the exome analysis, conventional significance was defined as p< 0.05, while Bonferroni correction was used as the threshold for multiple test correction across the number of proteins investigated (225) for cis-pQTL and combined (314) exGS analyses.”

9. *Table 2 only presents the statistically significant associations, but it would be valuable to include all 368 protein associations in the Supplementary Material to improve transparency.*

   Response: Thank you for the reviewer’s comment. The results for all protein-cancer associations are provided in the **Supplementary Tables**: observational analyses (**Table S5**), cis-pQTL analyses (**Table S7**), exome analyses (**Table S8**), and Mendelian randomization (MR) analyses (**Table S9**). We have revised the main text to include references to **Supplementary Table S5** (**page 12, line 398**), **Supplementary Table S7** (**page 9, line 303**), **Supplementary Materials** section **S4.1** (**page 14, line 470**) and **Supplementary Materials** section **S4.2** (**page 14, line 474**).

10. *The study reports protein-protein correlations but does not provide correlation coefficients or statistical significance. If the proteins are highly correlated, mutual adjustment may introduce multicollinearity, making effect estimates unstable. The authors could consider checking for multicollinearity using variance inflation factor.*

    Response: Supplementary Figures S2 and **S3** present both the pairwise correlation coefficients between proteins and their corresponding significance levels (*p < 0.05; **p < 0.01; ***p < 0.001).

As described in the manuscript, the main analyses evaluate the association of each protein and prostate cancer risk individually, without mutual adjustment for other proteins ("Conditional logistic regression was used to estimate odds ratios (ORs) and 95% confidence intervals (CIs) for the association of each circulating protein concentration with risk of PC") [**page 7, lines 240-242**].

The section titled "Correlations between and mutual adjustment for selected proteins" is included to provide additional information about the level of inter-correlation among selected proteins. While all pairwise correlations were below 0.6, which suggests only moderate correlation, we also note that the corresponding variance inflation factor (VIF) for this level of correlation would be approximately 1.57 (VIF = 1 / (1 − r^2)), which is well below conventional thresholds for multi-collinearity concerns (typically VIF > 5 or 10).

Beyond statistical stability, examining protein-protein correlations and potential interactions is also important for biological interpretation. Correlated proteins may act within shared pathways or biological processes, and understanding these relationships can help identify candidate pathways underlying prostate cancer risk. To support this interpretation, we complemented the correlation analysis with protein–protein interaction network analysis (**Supplementary Materials**, Section **S3**), offering additional insights into how these proteins may jointly contribute to prostate cancer development.

11. *The study identifies PARP1 as significantly associated with PC risk in the exome analysis, but this finding was not supported in observational or MR analyses. It would strengthen the discussion to address potential reasons for this discrepancy, such as possible reverse causation in observational analysis, differences in the genetic architecture captured by exome analysis vs MR analysis. Additionally, several proteins were identified in only one type of analysis e.g. observational, exome, or MR/colocalization. Providing a more detailed discussion on the interpretation of these differences – including possible biological, technical, or statistical explanations – would help understand the findings and clarify their implications*

Response: We agree that there is some inconsistency between the association that we identify and externally replicated in the MEC, and the results from other methods in this manuscript. We note that there is directionally consistent but non-significant evidence for higher PARP1 associated with increased prostate cancer risk from our cis pQTL MR analysis. Genetic analyses are often framed as modelling the lifelong effect of an elevated exposure. In this case, the effect size for PARP1 implied by our exome analyses is relatively modest (5% increased risk per SD increase in PARP1) and we were likely underpowered with our observational data to observe these presumed lifelong effects which may be revealed in future studies with larger sample sizes.

In the case of not observing genetic evidence for proteins we identified for observational data, we agree this is the subject of much interest and we have discussed this in the Discussion, including for example for SPINT2 on **page 16, lines 544-550**:

> "Similarly, further work is needed to understand the associations of SPINT2 and NME3 with PC; while both were associated with an increased risk using MR, results from observational analyses suggested this association may be inverse. One explanation for this may be potential complexities arising from cis pQTL used to proxy protein levels; cis pQTL for SPINT2 were also splicing QTL with the protein increasing allele also being associated with high intron-excision ratio, which makes it challenging to ascertain whether MR analyses proxy relative binding affinity for these Olink assays to one over other proteoforms or overall protein abundance."

And also on **page 16-17, lines 581-587**:

"Limited or no support from MR and colocalisation analyses may also be due to other factors such as case mix in the cancer GWAS and genomic complexity of cis-protein regions, which remain challenging for colocalisation methods to resolve. Discrepancies between results from MR and exome analyses may stem from differences in the genetic variants that contribute to these methods; MR typically leverages largely intronic variants that may determine protein levels by altering the promotion or enhancement of transcription while exome variants may predict the abundance of differing functional protein sequences in the blood."

12. *SPINT2 was identified as candidate causal protein based on MR and colocalization analyses, but the colocalization results (Table S10) do not support a shared causal variant. The PP4 value was close to 0, while the PP3 was close to 1, suggesting that SPINT2 and PC are likely driven by distinct genetic signals rather than true colocalization.*

    Response: We observed multiple potential causal variants using the Susie method at the SPINT2 locus. Using this method we observed high PP4 for between SPINT2 and prostate cancer at this cis locus using rs7253245 (PP4: 0.97 and PP3=0.024). These results are presented in **Supplementary Table S10**.

Reviewer #4 (Remarks to the Author):

Reviewer comments for NMED-A138357

General Comments:

This manuscript presents an extensive statistical analysis investigating the role of inflammatory and immune-related proteins in prostate cancer (PC) risk using observational, genetic, and Mendelian randomization (MR) approaches. The study is well-structured, leveraging data from multiple large-scale cohorts (EPIC, UKBB, MEC, and PRACTICAL) and incorporating robust statistical methodologies. However, several points warrant clarification and further consideration.

Specific Comments:

1. *Could the authors provide details on the methods used to evaluate the proportional hazards assumption? This information would help in assessing the robustness of your Cox regression model results.*

Response: We evaluated the proportional hazards (PH) assumption using Schoenfeld residuals and found evidence of violation for 12 proteins at conventional significance levels. To address this, we conducted additional analyses incorporating a time-varying covariate (TVC) for these proteins. Only IL17A met the ENT threshold, with higher levels associated with a lower risk of prostate cancer in UK Biobank; an association not observed in EPIC. For IL15, the p-value decreased notably in the TVC analysis (PH p = 0.02 vs. TVC p = 0.007), consistent with the stronger protective effect seen in the long-lag analysis described in the paper. For DNER, the TVC model improved the p-value but did not meet the ENT threshold in UK Biobank and showed no supporting evidence in EPIC. Results for the remaining nine proteins were essentially unchanged. We have added these methodological details to the Methods (**page 8, lines 258–260**):

> "The proportional hazards (PH) assumption was evaluated using Schoenfeld residuals; where violations were identified, extended Cox models using time-varying effects for proteins were fitted."

and Results (**page 11, lines 372–376**):

> Evidence of PH assumption violations was identified for 12 proteins using Schoenfeld residuals. For these proteins, extended Cox analyses revealed a significant association of higher IL17A with lower prostate cancer risk in the UKBB; no such association was observed in EPIC. For IL15 and DNER, p-values improved but did not meet the ENT threshold, and results for the remaining nine proteins were materially unchanged.

Figure x: Time-varying hazard ratio for selected proteins with 95% confidence interval in the UK Biobank

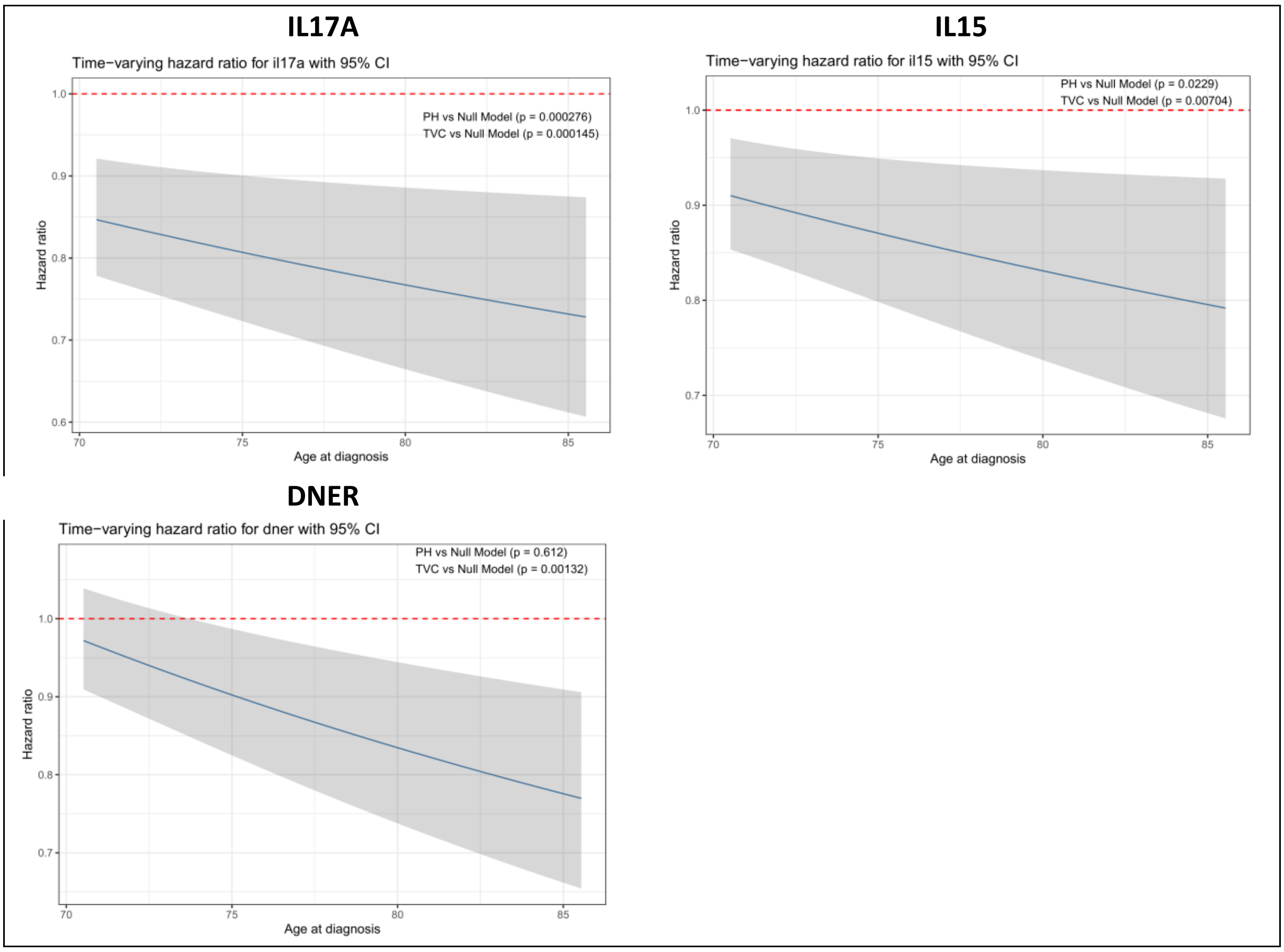

2. *The authors used the effective number of tests (ENT) for multiple testing correction. While this is a reasonable approach, can the authors provide a justification for using ENT instead of stricter family-wise error rate (FWER) controls?*

   Response: Thank you for this helpful comment. We used the effective number of tests (ENT) approach to correct for multiple testing, as it accounts for the correlation structure among the tested proteins and provides a balance between controlling false positives and retaining statistical power. While stricter family-wise error rate (FWER) methods such as Bonferroni correction are more conservative, they assume independence between tests and may be overly stringent in high-dimensional omics data where substantial correlation exists among features. We have now added the following sentence to the methods section on **page 8, lines 279-282** to justify our choice:

   > "To account for multiple testing, we applied the Effective Number of Tests (ENT) approach, which reflects the correlation structure among proteins and provides a less conservative correction than Bonferroni while still addressing the risk of false discoveries."

3. *The selection criteria for proteins in subgroup analyses should be clarified. Were all proteins tested in stratified models, or only those significant in the primary analysis?*

   Response: Thank you for the comment. All 368 proteins were included in the subgroup analyses, regardless of their significance in the primary analysis. We have clarified this point in the Methods section (**page 7, line 249**).

“These analyses were repeated for all proteins in subgroups of time from blood collection to diagnosis (<3 years [n=206], 3-7 years [n=311], and >7 years [n=917]), age at diagnosis (≤67 years/> 67 years), and prostate tumour stage (localised/advanced), histological grade (low-intermediate/high), and aggressiveness (aggressive/non-aggressive).”

4. *The authors mention Bayesian colocalization to confirm shared causal variants. Since colocalization does not always confirm causality, did the authors check for LD structure or perform alternative instrumental variable selection strategies?*

   Response: We thank the reviewer for raising this important point. Beyond our Bayesian colocalization analysis, we applied SuSiE fine-mapping (Wang et al., 2020) to each cis-pQTL region, explicitly modelling multiple causal variants and local LD structure. SuSiE produces credible sets of jointly associated variants, from which it selected conditionally independent instruments even in complex LD blocks. We then repeated colocalization using the SuSiE-derived signals, which is more robust to LD confounding. We agree that no single analysis can definitively establish causality. Instead, we believe that our complementary strategy, integrating prospective cohort data with orthogonal genetic methods, provides convergent evidence to guide future experimental studies which may highlight likely causal pathways in prostate cancer aetiology.

5. *The models adjusted for standard confounders (age, BMI, smoking, education, etc.), but were inverse probability weighting or other methods considered?*

   Response: Thank you for your suggestion. Inverse probability weighting (IPW) is particularly useful when confounding is strong and not adequately addressed by standard covariate adjustment. In our study, we adjusted for protentional confounders such as exact age, BMI, smoking, alcohol intake, education, and diabetes status. Among these, age is the primary potential confounder, but cases and controls were matched with controls on age within six months, minimising residual confounding. Other covariates, such as BMI and diabetes, showed only moderate associations with prostate cancer and did not exhibit strong imbalances (see **Table 1**).

   While IPW can offer additional robustness, it also introduces potential limitations, including sensitivity to model misspecification and extreme weights. Given the characteristics of our data, we considered standard adjustment appropriate for our primary analyses. We agree, however, that IPW or similar approaches may be valuable in future sensitivity analyses.

6. *Were interactions tested between key proteins (e.g., IL15, FLT3LG) and known PC risk factors, such as genetic risk scores for PC?*

   Response: Investigating interactions between key proteins (e.g., IL15, FLT3LG) and established prostate cancer risk factors, such as 451 polygenic risk scores and other rare variants like BRCA2, is an important next step, but is beyond the scope of the current manuscript.

7. *The paper suggests that higher levels of immunosurveillance-related proteins are protective, while higher PARP1 increases risk. Given that observational and genetic estimates for some proteins were discordant, can more discussion on possible explanations be provided?*

   Response: We agree that there is some inconsistency between the association that we identify and externally replicated in the MEC, and the results from other methods in this manuscript. We note that there is directionally consistent but non-significant evidence for higher PARP1 associated with increased prostate cancer risk from our cis pQTL MR analysis. Genetic analyses are often framed as modelling the lifelong effect of an elevated exposure. In this case, the effect size for PARP1

implied by our exome analyses is relatively modest (5% increased risk per SD increase in PARP1) and we were likely underpowered with our observational data to observe these presumed lifelong effects which may be revealed in future studies with larger sample sizes.

In the case of not observing genetic evidence for proteins we identified for observational data, we agree this is the subject of much interest and we have discussed this in the Discussion, including for example for SPINT2 on **page 16, lines 544-550**:

> "Similarly, further work is needed to understand the associations of SPINT2 and NME3 with PC; while both were associated with an increased risk using MR, results from observational analyses suggested this association may be inverse. One explanation for this may be potential complexities arising from cis pQTL used to proxy protein levels; cis pQTL for SPINT2 were also splicing QTL with the protein increasing allele also being associated with high intron-excision ratio, which makes it challenging to ascertain whether MR analyses proxy relative binding affinity for these Olink assays to one over other proteoforms or overall protein abundance."

And also on **page 16-17, lines 581-587**:

> "Limited or no support from MR and colocalisation analyses may also be due to other factors such as case mix in the cancer GWAS and genomic complexity of cis-protein regions, which remain challenging for colocalisation methods to resolve. Discrepancies between results from MR and exome analyses may stem from differences in the genetic variants that contribute to these methods; MR typically leverages largely intronic variants that may determine protein levels by altering the promotion or enhancement of transcription while exome variants may predict the abundance of differing functional protein sequences in the blood."

8. *The statement that IL15 and FLT3LG could be targets for PC prevention is intriguing but should be phrased cautiously.*

   Response: We agree that at this point cautious optimism is warranted. Our interpretation is limited to what we report in the current manuscript and previous evidence for the importance of IL15 and FLT3LG pathways in prostate cancer. For example, our observational evidence is consistent with involvement of the IL15–FLT3LG axis in prostate cancer aetiology and functional and experimental evidence describe the role of IL15 is reducing tumour progression phenotypes. However, we agree that further replication and functional validation is necessary. We therefore restrict our conclusions to suggesting that IL15-FLT3LG related biological process like immunosurveillance, be priorities for further research, which may in time inform prevention strategies.

9. *While the authors attempt replication in MEC, it is unclear how well findings generalize to non-European populations.*

   Response: We thank the reviewer for raising this important point. As mentioned in the discussion, the majority of our genetic analyses were conducted in individuals of European ancestry. Nonetheless, we were able to replicate our key finding for PARP1 and prostate cancer risk in the Multiethnic Cohort (MEC), which included participants from several non-European populations (White: 555 cases; Latin American: 602; Japanese: 1,006; Black: 538; Hawaiian: 182). This suggests that, at least for this locus, the signal observed in the UK Biobank is consistent across ancestries. However, we also note that the number of non-European cases remains relatively limited, and only a few large-scale datasets to date have directly measured plasma protein levels in diverse

populations. Expanding such resources will be an important priority for future research. We discuss replication of PARP1 in the MEC in the Discussion on **page 16, lines 552-555**:

> "We also identified genetically predicted higher PARP1 to be associated with an increased risk of PC overall using exome protein score analyses and were successful in replicating this association in an external, diverse prospective cohort consisting of African American, European American, Japanese American, Latino, and Native Hawaiian men."

and **page 16, lines 567-571**:

> "However, we note that the data in this analysis were majority participants in EPIC that are predominantly of European ancestry and that almost all cases in our UK Biobank analyses are also of European ancestry; the promise of these initial proteomics results further underscores the need for the prompt diversification of studies on proteins and cancer and the assessment of the generalisability of findings across populations with differing ancestries."

10. *The sample sizes for aggressive PC and >7 years analyses are smaller. Could power limitations explain the null findings for some proteins in aggressive disease?*

Response: Thank you for the comment. We agree that the statistical power is more limited in the subgroup analyses because of the smaller number of cases, leading to wider confidence intervals and greater likelihood of findings not passing the significance threshold. With 1,434 cases overall, we would have 76% power to detect a relative risk of 0.85 for prostate cancer, whereas for aggressive disease, with 488 cases, the power would be only 13% ($\alpha = 0.05/189$). As shown in Figure 3, FLT3LG, PGF, and BCL2L11 were associated with aggressive disease at conventional significance levels, and IL15 and FLT3LG also showed protective associations with advanced stage. These findings are directionally consistent with the overall analysis but were not sufficiently powered in the subgroup analysis. We have revised the Discussion on **page 17, lines 594-597** to comment on this limitation.

> "Finally, number of cases was limited for PC subtypes and for some cancers included in our cross-cancer analysis, leading to more limited statistical power to detect associations between protein level and risk of these specific cancer endpoints."

This is a well-conducted study employing rigorous statistical methods to investigate the role of immune-related proteins in PC risk. Addressing the points above would strengthen the manuscript's conclusions.

Reviewers' Comments:

Reviewer #1 (Remarks to the Author):

Major Comments

1. *The manuscript presents the results as a series of descriptive associations without a unifying conceptual framework. Clarifying the central hypothesis and how the study builds on existing knowledge would help frame the results. For example, emphasizing the protective role of immunosurveillance and its implications for PC prevention could tie the findings together.*

   Response: Our study aimed to provide a comprehensive, agnostic evaluation of inflammation and immune-related proteins in relation to prostate cancer risk, using large-scale proteomic, genetic, and replication analyses across multiple cohorts. However, based on the limited available prospective epidemiological data and on the conventional wisdom that prostate cancer is an immunologically cold tumour, we did not anticipate the potential role of immune surveillance a priori. This is why we did not lead with this hypothesis in the Introduction, and rather discussed this emerging theme in the Discussion, where we reflect on the potential protective roles of immune-related pathways, such as those involving the recruitment and activation of natural killer and T cells, and their possible relevance for prostate cancer prevention.

2. *The introduction should briefly summarize prior studies on inflammation and immune markers in PC to provide better context for the knowledge gap this study addresses. This would set the stage for the novel contributions of the current work.*

   Response: As we have described in the introduction, there are relatively few prospective observational data on PC in relation to the majority of inflammation and immune related proteins. The majority of studies investigated the association with CRP and results from meta-analyses are inconsistent. We revised the Introduction and added more details about these studies on **page 3-4, lines 106-115**.

   > The role of inflammation and the immune system in cancer aetiology is complex. While chronic inflammation is a hallmark of cancer and can promote cellular proliferation, immune and inflammation-related signalling can also detect and eliminate abnormal cells, preventing tumorigenesis via immunosurveillance.[1-3] To date for prostate cancer (PC), there is suggestive epidemiological evidence for an association with a limited number of relevant candidate proteins, such as C-reactive protein and Interleukin (IL) 6[4]. A systematic review and meta-analysis of inflammatory markers and cancer risk, which included up to seventeen studies of prostate cancer, found CRP to be the most frequently examined marker; although most studies reported null findings, pooled prospective estimates revealed a weak but statistically significant association between elevated CRP and PC risk. IL6 showed no consistent effect, with one study reporting a negative association and three studies reporting null findings.[4] More recently, a prospective analysis from the Osteoporotic Fractures in Men (MrOS) cohort reported that moderate or high IL10 levels were associated with substantially lower PC risk, with no significant associations for IL6, CRP, TNF-α, or overall inflammatory burden.[5] Despite

these insights, prospective observational data on most immune and inflammation-related proteins in PC remain scarce.[6]

3. *The authors should clarify why EPIC included fewer controls compared to UK Biobank. Could this difference impact statistical power or result comparability?*

   Response: Thank you for this comment. The difference in the number of controls comes from the underlying study designs. EPIC used a 1:1 nested case-control design, resulting in an equal number of cases and controls. In contrast, UK Biobank is a prospective cohort study, where the number of controls is determined by the overall cohort size and the number of cancer cases occurring during follow-up.

   Both the number of events and study design influence statistical power. Figure 1 shows the power analysis for EPIC and UK Biobank, based on the observed number of cases and their respective study designs. Although EPIC has lower statistical power than the UK Biobank, this does not necessarily affect the comparability of results. Both prospective studies used the same proteomic platform (Olink technology), ensuring consistency in biomarker measurements. As shown in Figure S1 of the **Supplementary Materials**, the significant point estimates are broadly consistent between the two studies, although confidence intervals are wider in EPIC, as expected.

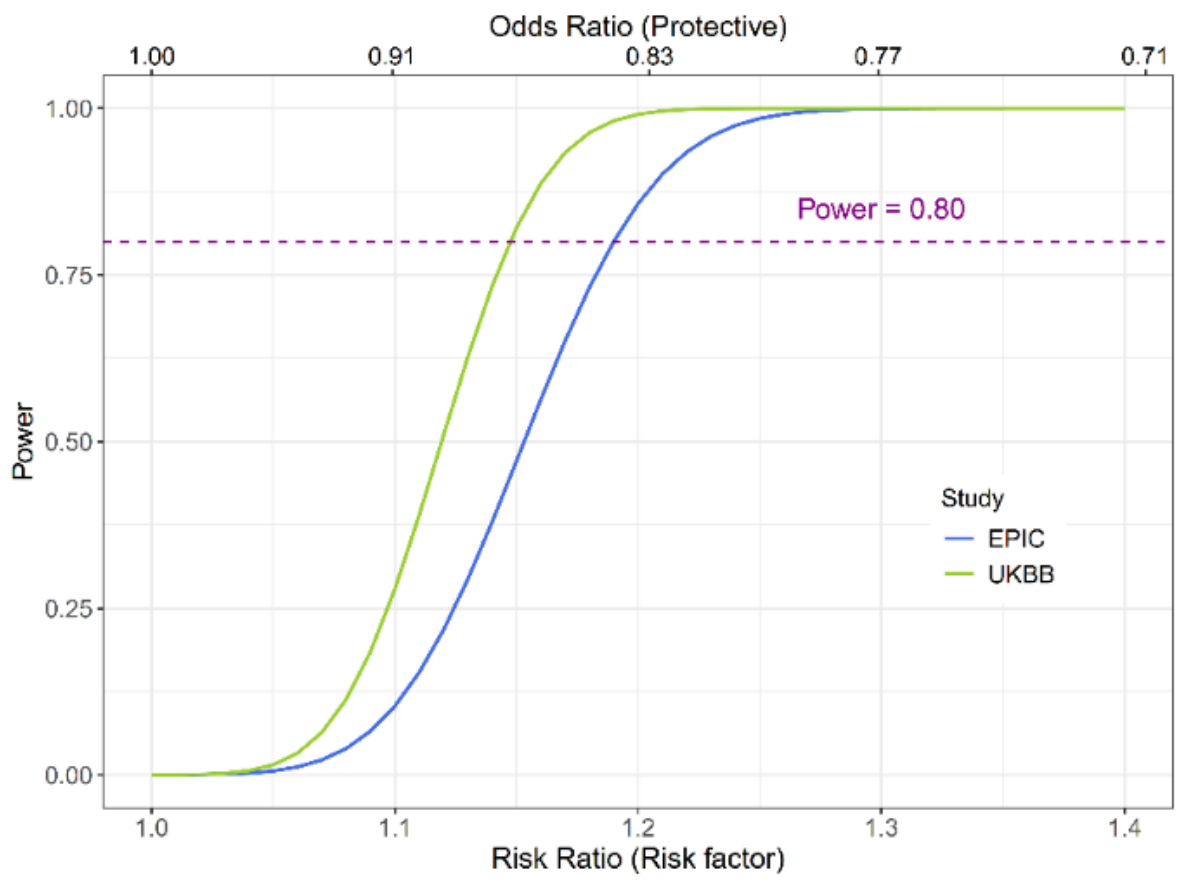

Figure 1: Statistical power analysis for EPIC and UKBB

4. *Have you considered conducting non-linear analyses for the observational data?*

   Response: We evaluated potential non-linear associations for all 368 inflammatory proteins using natural splines with three degrees of freedom. While adding non-linear terms improved model fit for some proteins, it did not materially change the overall findings. Consistent with the linear analyses, no proteins in EPIC passed the ENT threshold in the overall analysis. In the lag-time analysis, IL15 was the only protein to surpass the ENT threshold in the non-linear model, and this association was also significant under the linear model.

   In UK Biobank, non-linear analyses confirmed that CNTNAP2 and FLT3LG remained significant in the overall analysis, as observed in the linear models. In the long-lag analysis, BCL2L11 and FLT3LG-

both significant under the linear model-did not meet the ENT threshold when non-linear terms were included, likely reflecting reduced power from increased model complexity. In the short-lag analysis, CNTNAP2 remained the only protein significant under both linear and non-linear models.

5. *The manuscript does not specify the significance threshold for SNP selection in the Mendelian Randomization (MR) analysis. Including this information is essential for reproducibility and understanding the robustness of the findings.*

   Response: We have now updated the text (**page 9, line 315**) to specify that we chose genome-wide significance as the threshold to select cis pQTL for inclusion in our MR analyses.

   > "*Cis*-pQTLs meeting genome-wide significance (p-value<5e-8) and independence at r2 < 0.01 were extracted from the protein GWAS by Sun et al. involving 34,557 UKBB participants of European ancestry, and those with weak instrument strength (i.e. F-statistics < 10 [β2/σ2]) were excluded from our study."

6. *How many SNPs were available as cis-instruments for the proteins analyzed? Reporting this would clarify the strength of the genetic instruments used in MR.*

   Response: After we performed quality control for cis pQTL and harmonisation with prostate cancer GWAS summary statistics, including a search for proxy variants, cis pQTL were available for 285 out of 368 proteins in our study. More details on the eligible cis-SNPs for each protein is presented in Supplementary Table S9. We have modified the manuscript on **page 10, lines 327-328**:

   > "Harmonised SNPs were oriented to the protein-increasing allele and 285 proteins were included in cis pQTL MR analyses in this study."

   and **page 13, line 439-440**:

   > We also conducted MR analyses for PC for the 285 (out of 368) proteins in our study that had available cis-pQTLs (**see Supplementary Table S9** and **Supplementary Table S11** for complete MR results, and the number of eligible cis-SNPs for each protein, respectively).

   Supplementary Table 11 (Number of cis-pQTL per protein in MR analysis) is added to the revised version and below as well:

Number of cis-pQTL per protein	Overall Prostate Cancer	Aggressive Prostate Cancer	Early-onset Prostate Cancer
1	110 (38.60)	108 (39.13)	108 (39.13)
2	94 (32.98)	87 (31.52)	88 (31.88)
3	44 (15.44)	45 (16.30)	44 (15.94)
4	23 (8.07)	23 (8.33)	23 (8.33)
5	7 (2.46)	7 (2.54)	7 (2.54)
6	4 (1.40)	3 (1.09)	3 (1.09)
7	2 (0.70)	2 (0.72)	2 (0.72)
8	1 (0.35)	1 (0.36)	1 (0.36)
Total	285	276	276

7. *The manuscript would benefit from incorporating multivariate MR to address potential pleiotropic effects of metabolites that might confound protein-PC associations.*

   Response: We agree that multivariate MR can be a valuable tool to investigate the potential influence of pleiotropy. However, in the cis-pQTL MR analyses more often than not, there was only one genetic variant available for analyses; no multivariate MR methods are currently possible in this scenario.

8. *State explicitly whether a fixed-effects or random-effects model was used in the inverse-variance weighted (IVW) MR analysis. This improves transparency in the statistical approach.*

   Response: Cis pQTL MR analyses in this manuscript used Wald ratios as described in the methods section due to the general availability of only one cis pQTL per protein after clumping at $r2 < 0.01$. No inverse-variance weighted models were performed and we have now updated the methods section to reflect this.

9. *The MR section should include estimates from MR-Egger and the weighted median (WM) to account for directional pleiotropy. Bi-directional MR could also help evaluate potential reverse causality.*

   Response: Thank you for this suggestion. Due to the availability of only a single cis-pQTL instrument for most proteins, we performed MR analyses using the Wald ratio method. MR-Egger and weighted median approaches require multiple independent instruments per exposure to provide valid estimates, which was not feasible in our analysis. We have now conducted bi-directional MR and added the corresponding methods (**page 10, lines 341:348**) and results (**page 13, lines 452-455**) and in **Supplementary Table S12**.

10. *The robustness of the colocalization analysis to prior assumptions should be discussed. Were alternative priors tested? This is particularly important given the complexity of cis-pQTL regions.*

    Response: We assessed the sensitivity of our colocalization results to the choice of prior using the in-built functions as part of the coloc package. Choice of prior (p12 ranging from 1e-4 to 1e-6) was not observed to materially alter the conclusions of these tests in our data. A sentence has been added to Methods on **page 10, lines 336-339:**

    > "Within each predefined genetic region (250 kb around the index cis-pQTL), we tested for the presence of a single shared causal variant between protein and cancer using conventional colocalisation, as well as multiple shared causal variants using the Sum of Single Effects (coloc SuSiE) regression method, which can account for the presence of multiple causal variants of which only a subset are shared between two traits. Default priors were used for analyses and PP4 > 0.7 was accepted as evidence for colocalisation; we also assessed the sensitivity of our colocalization results to the choice of priors (with p12 ranging from 1e-4 to 1e-6)."

    and to the Results on **page 13, lines 448-450**:

Colocalisation analyses showed support for a shared causal variant between SPINT2 (OR:2.11, 95%CI:1.70-2.63, PP4: 97%) and NME3 (1.25, 1.11-1.41, PP4: 71%) and these conclusions were generally robust to the choice of priors (i.e. SPINT2 colocalised when p12= 1e-4 to 1e-6, while NME3 colocalised when p12=1e-4 to 1e-5).

11. *The authors employed MR to infer causality. However, given the complexity of cis-pQTL regions, further elaboration on the methodology used for colocalization analysis is needed. Specifically, did the coloc-SuSiE model account for multiple causal variants, and if so, how was this implemented?*

Response: Our colocalization analyses employed both naïve colocalisation and also the R **coloc** package's coloc.susie() function, which first fine-maps each trait with SuSiE (allowing up to 10 independent signals per locus using the local LD matrix) and then integrates those "single-effect" credible sets to compute Bayes factors and posterior probabilities of shared causality. In doing so, this method is explicitly colocalise and testing for multiple causal variants rather than assuming a single effect. Further details to this effect have now been added to the manuscript on **page 10, lines 336-339**.

"Within each predefined genetic region (250 kb around the index cis-pQTL), we tested for the presence of a single shared causal variant between protein and cancer using conventional colocalisation, as well as multiple shared causal variants using the Sum of Single Effects (coloc SuSiE) regression method, which can account for the presence of multiple causal variants of which only a subset are shared between two traits. [24] Default priors were used for analyses and PP4 > 0.7 was accepted as evidence for colocalisation; we also assessed the sensitivity of our colocalization results to the choice of priors (with p12 ranging from 1e-4 to 1e-6)."

12. *The time-stratified analysis is a strength. However, the authors should elaborate on the biological mechanisms that might explain the stronger associations seen in samples taken more than 7 years before diagnosis.*

Response: We thank the reviewer for this helpful suggestion. The stronger associations in blood collected more than seven years before diagnosis are consistent with potential endogenous processes/factors acting early in prostate cancer aetiology rather than markers of subclinical disease. Prostate cancer often evolves slowly and typically becomes immunologically cold, with limited immune infiltration in established tumours. Circulating immune proteins measured well before diagnosis may therefore reflect host immunosurveillance and tissue homeostasis at a time when these processes are intact, rather than reflecting inflammatory responses to an existing lesion. For example, higher IL-15, a cytokine that supports NK- and CD8-cell function, was more clearly associated with lower prostate cancer risk in samples taken more than seven years before diagnosis. This temporal pattern reduces the likelihood of reverse causation in determining our findings and instead supports an interpretation that aspects of systemic immune competence influence risk during earlier stages of tumour development. In the Discussion (**page 14-15, lines 494-500**), we have explained this rationale with the findings being compatible with an aetiologically relevant window long before diagnosis.

> "Our findings are consistent with potential endogenous processes/factors acting early in prostate cancer aetiology rather than the associations reflecting markers of subclinical disease. Prostate cancer often evolves slowly and typically becomes immunologically cold, with low tumour infiltrating lymphocytes, such as active T cell and NK cells, in tumour tissue compared to tissue from healthy prostates.[3,25] Circulating immune proteins measured well before diagnosis may therefore reflect host immunosurveillance and tissue homeostasis at a time when these processes are intact, rather than reflecting inflammatory responses to an existing lesion."

13. *Most genetic analyses were based on individuals of European ancestry. While this limitation is acknowledged, the authors should provide a more detailed discussion of how this affects the generalizability of their findings, particularly in the context of multi-ethnic populations.*

Response: We thank the reviewer for raising this important point. As mentioned in the discussion, the majority of our genetic analyses were conducted in individuals of European ancestry. Nonetheless, we were able to replicate our key finding for PARP1 and prostate cancer risk in the Multiethnic Cohort (MEC), which included participants from several non-European populations (White: 555 cases; Latin American: 602; Japanese: 1,006; Black: 538; Hawaiian: 182). This suggests that, at least for this locus, the signal observed in the UK Biobank is consistent across ancestries. However, we also note that the number of non-European cases remains relatively limited, and only a few large-scale datasets to date have directly measured plasma protein levels in diverse populations. Expanding such resources will be an important priority for future research. We discuss replication of PARP1 in the MEC in the Discussion on **page 16, lines 553-556**:

> "We also identified genetically predicted higher PARP1 to be associated with an increased risk of PC overall using exome protein score analyses and were successful in replicating this association in an external, diverse prospective cohort consisting of African American, European American, Japanese American, Latino, and Native Hawaiian men."

and **page 16, lines 567-572**:

> "However, we note that the data in this analysis were majority participants in EPIC that are predominantly of European ancestry and that almost all cases in our UK Biobank analyses are also of European ancestry; the promise of these initial proteomics results further underscores the need for the prompt diversification of studies on proteins and cancer and the assessment of the generalisability of findings across populations with differing ancestries."

14. *The study analyzes 368 proteins, representing only a fraction of the immune proteome. Discussing potential proteins of interest for future research would be beneficial.*

Response: We agree that 368 proteins represent only a portion of the immune proteome. We selected the Olink Explore Inflammation I panel as a first investigation into many common and notable bioamrkers of the immune system, while recognising that it does not capture the full breadth of immune signalling or cell type specific markers.

With large-scale high-throughput proteomics becoming available in population cohorts, including Olink HT and emerging Illumina protein-prep workflows both in the UK Biobank, it will be possible to deepen coverage across pathways such as soluble checkpoint signalling, innate sensing, myeloid and NK surveillance, complement, and stromal-immune crosstalk.

At present, however, our study provides early evidence that prospective measures of the circulating immune system carry information relevant to prostate cancer aetiology. Expanding coverage in future work may help refine mechanistic hypotheses and nominate additional biomarkers or preventive targets.

We addressed this limitation in the discussion on **page 17, lines 589-591** and as below:

> "Further, we investigated a sizable but nonetheless still modest subset of all immune and inflammation-related proteins. Therefore, the seven proteins that we identify in this study likely represent only a fraction of proteins with an important role in PC aetiology. With large-scale high-throughput proteomics increasingly available in population cohorts (e.g. Olink HT and emerging Illumina protein-prep workflows in UK Biobank), it will be possible to broaden coverage beyond the panel we studied. Deeper characterisation of the proteome in larger sample sizes from mature prospective cohorts in the future will be needed to bring greater resolution to the network of the PC risk proteome - in particular, proteins that might be linked to a positive feedback loop for IL-15 involving NK cells, DC and T lymphocytes."

15. *The identification of PARP1 as a risk protein is noteworthy, but additional functional validation (e.g., in vitro or in vivo studies) would strengthen the evidence base.*

Response: We agree with the reviewer that functional validation is an important step in confirming the causal role of the identified proteins. While in vitro or in vivo studies were beyond the scope of this epidemiological investigation, we have added the following sentence to the Discussion (**page 16, lines 562-564**):

> "Further investigation, including functional validation, is needed to elucidate its mechanistic role in early-stage disease."

Minor Comments

16. *Figure 1: Ensure consistency in the reported number of proteins (386 vs. 368). Cross-check with the supplementary tables and correct any discrepancies.*

Response: Thank you, we have checked these numbers.

17. *Figure 5: Include sample sizes for each cohort in the forest plots for PARP1 to provide better context.*

Response: We have revised the graph and added in the number of cases.

18. *The leave-one-out plots for MR analysis need to be redrawn for improved legibility.*

Response: Thank you for your comment. We did not perform leave-one-out analyses for MR in this study. Could you please clarify if this comment refers to another plot in the manuscript that requires improvement? We would be happy to revise accordingly.

19. *Expand citations related to immunosurveillance and dendritic cell roles in PC, including recent advances in checkpoint inhibitors.*

Response: We thank the reviewer for this helpful suggestion. We have expanded the Discussion with additional citations on immunosurveillance (**page 14, line 498**), the role of dendritic cells in prostate cancer, and recent advances in immune checkpoint inhibitors on **page 14-15, lines 494-500**:

"Our findings are consistent with potential endogenous processes/factors acting early in prostate cancer aetiology rather than the associations reflecting markers of subclinical disease. Prostate cancer often evolves slowly and typically becomes immunologically cold, with low tumour infiltrating lymphocytes, such as active T cell and NK cells, in tumour tissue compared to tissue from healthy prostates.[3,25] Circulating immune proteins measured well before diagnosis may therefore reflect host immunosurveillance and tissue homeostasis at a time when these processes are intact, rather than reflecting inflammatory responses to an existing lesion."

and also on **page 15, lines 523-530**:

"We have previously reported an inverse association between FLT3LG and PC risk using both observational and using exome analyses.[6] Here we additionally demonstrate, as with IL15, its strong inverse association with risk in men who developed PC longer after having given blood. FLT3LG is primarily produced by NK cells and controls the development of dendritic cells via binding to FLT3 to increase the presentation of tumour antigens, as part of immunosurveillance that enhances T cell and NK cell activity.[33-35] In addition, FLT3LG is linked to higher levels of stimulatory dendritic cells within the tumour environment,[36] playing a pivotal role in enhancing the effectiveness of immune responses against cancer, including the improvement of outcomes in melanoma patients undergoing anti-PD-1 immunotherapy. Recent reviews further highlight the importance of dendritic cell priming in prostate cancer immunobiology and as a determinant of responsiveness to immunotherapy.[37] Clinical studies also indicate that checkpoint inhibitors have limited overall benefit in unselected prostate cancer but can elicit durable responses in biomarker-defined subsets.[38]"

Reviewer #2 (Remarks to the Author):

The authors conducted large-scale observational studies and genetic analyses to evaluate the association between inflammation- and immune-related proteins and prostate cancer (PC) risk. This study identified both measured and genetically predicted levels of several proteins associated with PC risk. While the findings are of some merit and the study design is fundamentally sound, the following comments and suggestions may help the authors enhance their manuscript.

Major comments:

1. ***Method:*** *It is possible that multiple variants flanking a gene region affect the gene transcription and translation levels, which may be highly correlated with each other and be pleiotropic for multiple traits. The Wald ratios and IVW MR methods used in this study cannot address this issue. Have the authors tried other MR approaches such as cisMR-cML for this assumption?*

   Response: We agree that multiple correlated variants within cis-regions can influence gene transcription and translation, potentially introducing pleiotropy. In our analyses, most proteins had only a single independent cis-pQTL after clumping at $r^2 < 0.01$, which precluded the use of multi-instrument approaches such as MR-Egger, weighted-median MR, or cisMR-cML. Instead, we applied complementary strategies to address this assumption: (1) colocalization analyses using the coloc and coloc-SuSiE frameworks, which explicitly model multiple causal variants while accounting for local LD structure (**page 10, lines:333-339**); and (2) reverse MR analyses to evaluate potential reverse causation (**page 10, lines:341-348**). We have also revised the Discussion (**page 17, lines 575–583**) to acknowledge this limitation and note that residual pleiotropy within cis-regions cannot be entirely excluded.

2. ***Method:*** *May consider reverse MR, to estimate the effect of PC liability on levels of interesting proteins.*

   Response: Thank you. We have now conducted bi-directional MR and added the corresponding methods (**page 10, lines 341:348**) and results (**page 13, lines 452-455**) and in **Supplementary Table S12** (see also our response to **Reviewer 1**, **Comment 9**).

3. ***Method:*** *pQTL and ExGS were conducted using UK Biobank protein data and exome sequencing data, however, it is unknown which genotyping platform was used for the MEC cohort. Was MEGA-SNP array or exomechip used? If so, which SNVs were used in generating ExGS for selected proteins, especially PARP1, in UKB and were same SNVs used in MEC? How are the qualities of these SNVs in MEC? A large proportion of rare variants (MAF<0.005) had bad genotype clusters of low quality from SNP arrays or exomechips.*

   Response: We thank the reviewer for this important question regarding the MEC data used for PARP1. We have added a citation describing the MEC genetic protocol (**page 9, line 308**).

The MEC cohort was genotyped primarily using Illumina SNP arrays, with most remaining samples genotyped in 2020–2021 on the Illumina Global Diversity Array (GDA, including MEGA content); no exome sequencing or exome chips were used in MEC.

The PARP1 Exome Genetic Score (ExGS) was derived in UK Biobank using exome sequencing data. For MEC, the PARP1 ExGS was constructed using the same PARP1 SNVs from the UKBB-derived instrument and the scoring allele effect directions of increasing PARP1 levels. All PARP1 SNVs were common variants (MAF >1%).

Regarding variant quality, MEC genotypes underwent extensive QC followed by ancestry-specific imputation using the 1000 Genomes Project and TOPMed reference panels. For MEC, variants with MAF >1% have high imputation quality across racial and ethnic groups (mean R^2 ≥0.8), and no PARP1 SNVs included in the ExGS were poorly imputed. Rare variants (MAF <0.5%), which are known to have unreliable clustering on SNP arrays, were not included in the PARP1 ExGS in MEC.

Additionally, these details and a table describing the SNVs included in the PARP1 panel have been added to **Supplementary Materials S5**.

4. *Results from Mendelian randomization analyses: It would be nice if the authors list the variance explained by the instrumental cis variant(s) used in MR. And acknowledge the limitations of IV(s) selected of cis-pQTLs in Discussion.*

   Response: We agree that instrument strength is an important consideration in Mendelian randomization analyses. We therefore report the F-statistics for each cis-pQTL used as an instrument in **Supplementary Table S9**, indicating a limited risk of weak-instrument bias. For completeness, we have also added the proportion of variance explained by each cis-pQTL to this table. The limitations associated with using cis-pQTL instruments are acknowledged in the Discussion (**page 17, lines 575–583**).

5. *Results from Mendelian randomization analyses: cisMR support a few proteins for PC risk, which may reflect limited variance explained by selected cis-pQTLs. Please add effective allele of each cis-pQTL as well as EAF in exposure and outcome data sets. The section title seems confusing, should change to "MR analyses and colocalization". Colocalization is not part of MR but provides complementary evidence for a shared genetic component for the tested protein and PC. The later cannot determine association direction, in this case.*

   Response: We have revised the section title to "Mendelian randomisation and colocalisation analyses" to clarify that colocalization is presented as complementary evidence rather than as part of the MR framework. In addition, we have added the effect allele and effect allele frequency (EAF) for both exposure and outcome for each cis-pQTL used in the MR analyses to **Supplementary Table S9**, to improve transparency and reproducibility.

6. *Results in Fig. 5 and Table S8: I would suggest results of meta-analysis of UKBB and MEC: White be included in Part A. Details for used SNVs, MAF and quality, should be described.*

Response: We thank the reviewer for this helpful suggestion. We have now included the meta-analysis combining UK Biobank (UKBB) and the White (European American) subset of the Multiethnic Cohort (MEC) in Figure 5A, allowing an ancestry-matched comparison.

All genetic panels used in this study are publicly available on GitHub[1] (e.g. the PARP1 panel), including the final panel definition, a summary of SNV retention at each development step, and logs detailing SNV selection and linkage disequilibrium handling.

Population-level allele frequency information primarily follows Dhindsa et al. We have added **Supplementary Materials S5** to provide more details for PARP1 that lists, for each SNV, the minor allele frequency, gene annotation, and the most damaging predicted functional consequence. Panel-specific details are also provided.

All exome-based scores were derived from rigorously quality-controlled data. SNVs passed both the UK Biobank Pharma Proteomics Project QC and the standard UKBB exome sequencing QC pipeline, with additional internal filters excluding individuals and variants with >1% missingness. For MEC, replication relied on common variants imputed using array-based data; as reported in the MEC preprint, variants with MAF >1% showed high imputation quality (mean R^2 ≥0.8 across ancestries), and no SNVs in the PARP1 panel were poorly imputed. We have added a citation describing the MEC genetic protocol (**page 9, line 308**).

7. ***Discussion:*** *Limitations of the MR analysis used in this study (such as IVs and methods) need to be discussed. In addition, cis-pQTL may explain a limited proportion of variance of the nearby protein. Genetically determined protein levels by variants across the genome (eg, from GWAS on each protein) may greatly improve the statistical power in the MR analysis.*

Response: We agree that cis-pQTL typically explain a limited proportion of variance in circulating protein levels, which may reduce statistical power and lead to imprecise MR estimates. However, we note that all cis-pQTL instruments used in this study had F-statistics (**Supplementary Table S9**) well above the conventional threshold of 10, suggesting that weak instrument bias is unlikely to be a major concern in our analyses.

While genetically predicted protein levels using variants across the genome could increase explained variance, we deliberately focused on cis-pQTL instruments to minimize horizontal pleiotropy. For protein traits, trans-acting variants often influence protein levels via upstream regulators or shared biological pathways, which may violate the exclusion restriction and complicate causal interpretation. The cis-MR framework therefore represents a trade-off between statistical power and biological specificity.

We have revised the Discussion (**page 17, line 575-583**) to clarify these limitations and to emphasize that null or modest associations should be interpreted in the context of both the variance explained by cis instruments and their demonstrated strength based on F-statistics:

[1] https://github.com/Cancer-epi-unit/INF_PC

"Although cis-pQTL typically explain a modest proportion of variance in protein levels, all instruments used in our MR analyses had F-statistics above 10, suggesting adequate instrument strength. Nevertheless, for some proteins the variance explained was very low which limited our ability to detect modest effects. Furthermore, most proteins were instrumented by a single independent cis-pQTL, which precluded the application of multi-instrument MR methods designed to account for horizontal pleiotropy, and residual pleiotropy within cis-regions cannot be fully excluded. Limited or no support from MR and colocalisation analyses may also be due to other factors such as case mix in the cancer GWAS and genomic complexity of cis-protein regions, which remain challenging for colocalisation methods to resolve."

8. ***Discussion:*** *The discussion on replicated findings for the FLT3LG protein seems not adding more merit to this study.*

   Response: We agree that FLT3LG has been reported previously, including in our earlier work (Papier et al., 2024). Nonetheless, we consider it appropriate to retain FLT3LG in the Discussion, as its inclusion strengthens and extends the existing evidence rather than presenting it as a novel finding. Using data from EPIC, we provide an independent replication of this association, and the concordance of FLT3LG with other immune-related proteins identified in this study adds biological coherence to the emerging immunosurveillance signal.

9. ***Discussion:*** *More discussions on the possible connection between discovered proteins and immunosurveillance in PC are needed.*

   Response: We thank the reviewer for this valuable suggestion. We have expanded the Discussion (**page 14-15, lines 494:500**) to provide a more detailed interpretation of how the identified proteins relate to immunosurveillance and prostate-cancer aetiology. In particular, we highlight that IL15 and FLT3LG are key regulators of dendritic-cell and natural killer (NK)–cell activation, both of which are central to early tumour immune recognition (**page 15, lines 503-505**, and **page 15, lines 523-530**, respectively).

Minor comments:

10. *Typos and spelling errors, e.g., caption letter in "Nucleotide" in line 239, "mean" for "men" in line 243, "We note, however, we note" in line 539,…*

    Response: Thank you for noting these typos. We have corrected all identified errors and proofread the manuscript for consistency.

11. *FLT3LG has been reported as a risk protein for PC by the same group (ref 4) and should not be presented as an important finding.*

    Response: We have addressed this point in an earlier comment (**Reviewer 2**, **Comment 8**).

12. Lines 223-224: In previous study, EA measured protein values were log-base-2 transformed as normalized protein expression (NPX), which then subjected to inverse rank normal transformation. Can the authors clarify why they mean centered and standardized the NPX values instead?

Response: In our earlier work, inverse rank normalisation was used to improve the normality of protein distributions. In the present study, however, based on experience comparing results across proteomic platforms and before and after rank-based transformation, we observed that inverse rank normalisation can affect estimates for a small number of proteins. We therefore chose to mean-centre and standardise NPX values, which are already log2-transformed by design, in order to preserve relative differences and avoid unnecessary loss of information. We do not expect major differences between approaches, as both rely on the same underlying measurements and scaling, with the only distinction being the final step that enforces a normal distribution in the previous publication.

13. Reference 14 in line 284 overlaps with reference 4.

Response: Thank you for noting this. We have corrected the duplication in the reference list.

14. Line 309: LD clumping cutoff value r2<0.05, a typo for 0.01?

Response: We have revised it in the text (**page 9, line 291**)

15. Lines 354-355, it would be nice to indicate the version of the coloc package

Response: We have added the version of the coloc package to the Methods section, (**page10, line 352**).

16. Method: Mendelian randomization analyses: This section may be "Mendelian randomization and colocalization analyses".

Response: We have revised the section title to "Mendelian randomization and colocalization analyses", as suggested.

17. Results of "Exome analyses" in Table S7, many SNVs (ID) with discordant directions between OR and Z_STAT, please correct them.

Response: We have updated the exome analysis results in Table S7 and corrected the inconsistencies between the directions of the odds ratios and the corresponding Z-statistics. The revised table now reports internally consistent effect directions throughout.

18. Discussion: line 481, lines 516 and 517, and line566, where are the results for protein and PC association for cases diagnosed 10 years/a decade after blood draw?

Response: We revised the text from more than ten to more than seven years for clarity. The rationale for the original wording was that the median follow-up time was 12.2 years, meaning that

a substantial proportion of cases were diagnosed at least a decade after the blood draw. We agree though that this could be confusing, and we have revised the text to ensure that the long-lag definition is described consistently throughout the manuscript.

19. Discussion: line 489, reference for "IL15 knockout mice also lack NK cells" is missing.

Response: Thank you for this comment. We have added the reference to the revised manuscript (**page 15, line 511**).

20. Discussion: lines 526-536, no references to support the hypothesis and views for the role of PARP1 in PC?

Response: We have added two references (**page 16, line 562**) to support the role of PARP1 in prostate cancer

Reviewer #3 (Remarks to the Author):

This study investigated the association between 368 inflammation and immune-related proteins and prostate cancer (PC) risk using observational data from EPIC and UKB. The authors further explored causality using exome-wide genetic scores, Mendelian Randomization (MR), and colocalization. The key findings suggest associations between several proteins and PCs risk based on observational analysis, and a positive association of PARP1 with PCs risk based on exome analysis. Additionally, SPINT2 and NME3 were suggested to play a role in PCs risk based on MR analysis.

Originality and significance:

1. *This study is valuable as it integrates large-scale proteomic and genetic datasets to explore the role of immune-related proteins in PC risk. This is an important area of research, as the study has the potential to contribute to our understand of PC immunology. However, to strengthen its impact, the authors may consider highlighting the novelty of their approach more explicitly. Previous studies have investigated proteomic signatures for PC using proteome-wide analyses rather than focusing on specific panels (e.g. Lin C et al 2024, medrxiv; Cheng L et al 2024, Discov Oncol; Sun W et al 2024, BMC Cancer). A clearer discussion of how this study advances beyond prior work would help clarify its unique contributions.*

   Lin C et al, Causal associations between plasma proteins and prostate cancer: a Proteome-Wide Mendelian Randomization, medRxiv 2024.09.17.24312688; doi: https://doi.org/10.1101/2024.09.17.24312688
   Cheng L, et al. Evaluation of circulating plasma proteins in prostate cancer using mendelian randomization. Discov Oncol. 2024 Sep 17;15(1):453. doi: 10.1007/s12672-024-01331-3
   Sun W et al. Associations between genetically predicted concentrations of plasma proteins and the risk of prostate cancer. BMC Cancer. 2024 Jul 27;24(1):905. doi: 10.1186/s12885-024-12659-y.

   Response: As noted in the introduction, there are limited prospective observational data on the majority of inflammation and immune-related proteins in relation to prostate cancer. The studies referenced by the reviewer, while valuable and scientifically important, are primarily based on Mendelian randomization analyses, which use genetic proxies (typically cis-pQTLs) to estimate the effects of protein levels, rather than relying on direct measurements.

   While MR is a powerful method for causal inference and can help overcome key limitations of observational studies, particularly by reducing confounding and reverse causation, it also has some limitations. Cis-pQTLs are not available for all proteins, and where they do exist, the strength and specificity of these instruments can vary, limiting the robustness of MR analyses. Genetic instruments may reflect specific isoforms or proteoforms, which do not always correspond to total circulating protein concentrations measured in plasma. MR estimates reflect lifelong genetic predisposition to higher or lower protein levels and may not capture the dynamic or time-dependent changes that are particularly relevant to disease initiation and progression. MR estimates may be affected by complex LD or pleiotropy unless addressed with colocalisation and sensitivity analyses.

   These limitations underscore the value of complementing MR with direct protein measurements in prospective studies, which allow for time-stratified analyses (e.g., by lag time) and may offer an additionally temporally relevant understanding of protein–disease associations. As mentioned in the introduction, the main novelty of the current study lies in its integration of prospective proteomic data with genetic evidence across multiple cohorts, enabling a triangulated analytical

framework. This approach improves biological interpretability, strengthens causal inference, and supports the prioritisation of candidate immune-related proteins for future investigation.

Additionally, the introduction could better establish the biological rationale by explaining how inflammation contributes to prostate carcinogenesis. The epidemiological claims about CRP and IL6 should be expanded with further discussion and additional citations to support their proposed role in PC.

Response: As we have described in the introduction, there are relatively few prospective observational data on PC in relation to the majority of inflammation and immune related proteins. The majority of studies investigated the association with CRP and results from meta-analyses are inconsistent. We revised the Introduction and added more details about these studies on **page 3-4, lines 106-115**.

The role of inflammation and the immune system in cancer aetiology is complex. While chronic inflammation is a hallmark of cancer and can promote cellular proliferation, immune and inflammation-related signalling can also detect and eliminate abnormal cells, preventing tumorigenesis via immunosurveillance.[1-3] To date for prostate cancer (PC), there is suggestive epidemiological evidence for an association with a limited number of relevant candidate proteins, such as C-reactive protein and Interleukin (IL) 6[4]. A systematic review and meta-analysis of inflammatory markers and cancer risk, which included up to seventeen studies of prostate cancer, found CRP to be the most frequently examined marker; although most studies reported null findings, pooled prospective estimates revealed a weak but statistically significant association between elevated CRP and PC risk. IL6 showed no consistent effect, with one study reporting a negative association and three studies reporting null findings.[4] More recently, a prospective analysis from the Osteoporotic Fractures in Men (MrOS) cohort reported that moderate or high IL10 levels were associated with substantially lower PC risk, with no significant associations for IL6, CRP, TNF-α, or overall inflammatory burden.[5] Despite these insights, prospective observational data on most immune and inflammation-related proteins in PC remain scarce.[6]

Beyond these, some methodological, statistical, and interpretation concerns need to be addressed to ensure the robustness of its conclusions. Below are some suggestions to improve the clarity, transparency, and the reliability of the results.

Data and methodology:

2. *This study includes only Germany, Italy, the Netherlands, Spain, and the UK study centres from EPIC cohort without providing a clear justification for excluding other EPIC study centers. If these exclusions were due to data availability or quality control, it would be useful to state this explicitly. Otherwise, it raises concerns about potential selection bias, as excluded centers may have different participant characteristics or follow-up durations.*

   Response: Thank you for your comment. While the full EPIC cohort includes participants from 10 countries, our analysis was limited to male participants from five countries (Germany, Italy, the Netherlands, Spain, and the UK) that contributed to the prostate cancer nested case-control studies and had available proteomic data for this analysis. This is mentioned in the method section (**page 4, lines 139-140**):

   > "The current study included male participants who had blood samples from centres located in Germany, Italy, the Netherlands, Spain and the United Kingdom."

3. *The exome analysis harmonized the genetic variants by orienting them to the protein-increasing alleles in exome analysis. However, some technical details remain unclear: (1) how were ambiguous variants (with A/T or C/G alleles) handled?, (2) were trans-pQTLs included in the exome-wide genetic score calculation?, and (3) how were pQTLs weighted in the genetic score calculations?*

   Response: Regarding (1), since this study is based on sequencing data from the same cohort as was used in Dhindsa et al, which were processed by the same pipeline and against the same reference genome, issues with ambiguous variants (e.g., A/T or C/G SNPs) are not a concern for our analyses. This is in contrast to genotype array data, where ambiguous SNPs and strand mismatches are a common source of harmonization errors.

   (2) Yes, trans-pQTLs were included in the exome-wide genetic score (exGS) calculation, provided they reached genome-wide significance ($P < 5\times10^{-8}$).

   (3) The weights used for constructing the exGS were the beta estimates reported in Dhindsa et al.[1], which were derived from pQTL effect sizes.

   Dhindsa, RS., et al. (2023). "Rare variant associations with plasma protein levels in the UK Biobank". Nature 622(7982): 339-47.

4. *MR analysis was used to assess causality but requires additional sensitivity analyses to ensure the robustness of results. Some key issues/missing details include: (1) how many of the 368 proteins have cis-pQTLs for MR analysis? (2) MR analysis in the MHC region e.g. CDSN, HLA-DRA, and HSPA1A is challenging due to extensive linkage disequilibrium. (3) How did the authors ensure that independent SNPs were used for MR and exome-wide genetic score analyses?, and (4) no discussion of horizontal pleiotropy, a major limitation in MR studies. It would be beneficial to include MR-Egger, MR-PRESSO, or weighted median sensitivity analyes to assess pleiotropy*

   Response: (1) As noted in the manuscript on **page 13, lines 438-439** we conducted MR analyses for prostate cancer on the 285 out of the 368 proteins that had available cis-pQTLs.

   > "We also conducted MR analyses for PC for the 285 (out of 368) proteins in our study that had available *cis*-pQTLs."

   (2) We agree that inference for cis pQTL within the MHC can be challenging and that no easily implementable solution is currently available. It is for this reason that while we report results for protein MR analyses where cis pQTL are located within the MHC, no colocalization has been conducted due to uncertainty in the methods' ability to resolve complex LD structures in this locus. This is discussed in the results section on **page 13, lines 442-445**:

   > "Three of these ten proteins, CDSN, HLA-DRA, and HSPA1A, were located within the MHC. While MR results for these proteins are reported for completeness, due to the complexity of LD and the sensitivity of colocalisation methods to LD references, we chose not to conduct colocalization analyses for cis-pQTL MR results located in the MHC region."

   (3) To ensure that only independent SNPs were included across the two analyses, the following approach was applied:

   For the exSC analysis, an LD matrix was generated using the entire European subset of the UK Biobank. Variants were selected if they were not correlated ($r^2 < 0.05$) within a 10 Mb sliding window, based on pairwise comparisons of the absolute Z-score (β/SE). The process was iteratively

repeated until the highest Z-scoring independent variants were retained, as described in our previous work by *Papier et al*.

For the cis-MR analysis, an LD matrix was generated using common SNPs from 20,000 randomly selected UK Biobank participants. Independent SNPs were defined as those with $r^2 < 0.01$ within a 10 Mb window.

Papier, K., et al. (2024). "Identifying proteomic risk factors for cancer using prospective and exome analyses of 1463 circulating proteins and risk of 19 cancers in the UK Biobank." Nature communications 15(1): 4010.

Additionally, an extra column titled "r^2 with cis-panel exSC SNPs" was added to **Supplementary Table S9** to indicate whether significant MR findings were in LD with SNPs selected in the cis-exSC analysis, for greater transparency. This LD assessment was performed using the LDlink tool with the high-coverage 1000 Genomes European reference panel.

(4) Horizontal pleiotropy is the concern to causal inference in MR. In the context of single-variant cis pQTLs MR analyses, methods such as MR-Egger, weighted median, and MR-PRESSO are not applicable because they require multiple independent instruments. We therefore used approaches suited to our design: colocalisation with SuSiE to support a shared causal variant, and Steiger filtering via reverse MR to identify potential reverse causation. Nonetheless, cis instruments are closer to the relevant biology and are therefore less susceptible to pleiotropy than trans or genome-wide instruments but not required, of course, to be immune. We have therefore revised the Discussion to acknowledge this limitation (**page 17, lines 578-581**).

> "Furthermore, most proteins were instrumented by a single independent cis-pQTL, which precluded the application of multi-instrument MR methods designed to account for horizontal pleiotropy, and residual pleiotropy within cis-regions cannot be fully excluded."

Statistical issues:

5. *The study reported differences in baseline characteristics between cases and controls (Table 1 and Supplementary Table 3) but does not report formal statistical tests. Without p values, it is unclear whether these differences are statistically significant.*

   Response: As recommended by the STROBE guidelines, we did not include p-values in **Table 1** because the purpose of baseline characteristics is to describe the study population rather than to make formal comparisons between cases and controls. Statistical testing of baseline characteristics in observational studies can be misleading, as any observed differences are a function of study design (e.g., matching or stratification) rather than random assignment. Instead, we present the actual values to allow readers to assess the clinical or epidemiological relevance of any differences.

6. *The study meta-analyzed hazard ratio (HR) from UKB and odds ratio (OR) from EPIC to estimate relative risk (RR) using inverse-variance weighted meta-analysis. However, HRs and ORs are not directly comparable, and thus this approach raises concerns about statistical validity. The authors could consider either estimating ORs from UKB and meta-analyzing them with EPIC results, or analyzing HRs from UKB and ORs from EPIC separately, treating one as a replicating cohort instead of meta-analyzing them together.*

   Response: While ORs from traditional case-control studies are not directly comparable to HRs, in nested case-control designs they can be interpreted as HRs under the proportional hazards

assumption[2]. We addressed this in the manuscript and cited the relevant reference on **page 8, lines 271-273**, stating: "Since cases and controls are matched on follow-up time in the nested case-control study design, the odds ratio can be interpreted as the hazard ratio, assuming the proportional hazards assumption holds." The theoretical basis is outlined in Section 5.4.1 of the cited reference and mentioned below:

"To summarize, table below represent the expected number of cases and controls by exposure group based on data generated by risk set sampling. At any time t, let $N_E(t)$ and $N_{\bar{E}}(t)$ be the number of individuals still at risk in the exposed and unexposed population, respectively; the number of cases expected in the exposed group at time t is then $N_E(t) \times h_E(t)$, by the definition of the hazard rate, $h_E(t)$, under exposure. Similarly, the number of cases expected in the unexposed group is $N_{\bar{E}}(t) \times h_{\bar{E}}(t)$. If m controls are sampled at time t, then the expected number of exposed and unexposed controls are simply $m \times \frac{N_E(t)}{N_E(t)+N_{\bar{E}}(t)}$ and $m \times \frac{N_{\bar{E}}(t)}{N_E(t)+N_{\bar{E}}(t)}$, respectively.

Table 1 Expected cell entries in a 2×2 contingency table for data generated by risk set sampling

		Case	Risk-set control
Exposure	E	$N_E(t) \times h_E(t)$	$m \times \frac{N_E(t)}{N_E(t) + N_{\bar{E}}(t)}$
	Not E	$N_{\bar{E}}(t) \times h_{\bar{E}}(t)$	$m \times \frac{N_{\bar{E}}(t)}{N_E(t) + N_{\bar{E}}(t)}$

Based on these expected numbers, the corresponding odds ratio (OR) is calculated as follows:

$$OR = \frac{ad}{bc} = \frac{[N_E(t) \times h_E(t)] \times \left[m \times \frac{N_{\bar{E}}(t)}{N_E(t) + N_{\bar{E}}(t)}\right]}{[N_{\bar{E}}(t) \times h_{\bar{E}}(t)] \times \left[m \times \frac{N_E(t)}{N_E(t) + N_{\bar{E}}(t)}\right]} = \frac{h_E(t)}{h_{\bar{E}}(t)} = RH(t)$$

Assuming proportional hazards, the relative hazard RH(t) is constant over time, and the odds ratio derived at each time point approximates this constant hazard ratio."

[2] Jewell NP. Study designs. Statistics for Epidemiology. New York: Chapman and Hall/CRC; 2004.

7. *Several results are described in the result section without explicitly reporting ORs, HRs, 95% confidence intervals, or p-values. This makes it difficult to assess the statistical robustness.*

Response: Thank you for your comment. We have revised the manuscript to include ORs and 95% confidence intervals in the Results (**page 12, lines 393-395**):

"In EPIC, no proteins were associated with high grade, advanced stage, or aggressive PC after accounting for multiple testing. However, we identified inverse associations at conventional significance for IL15 (0.74, 0.60-0.91) and FLT3LG (0.75, 0.61-0.92) with advanced stage and BCL2L11 (0.82, 0.70-0.97), PGF (0.83, 0.71-0.98), and FLT3LG (0.85, 0.74-0.99) with aggressive PC subtypes (Error! Reference source not found.)."

and on **page 13, line 460**:

"Concordant with observations from our observational analyses from meta-analyses in EPIC and UKBB, we found evidence at conventional significance in exome protein score analyses for inverse

associations of FLT3LG (OR:0.97, 95%CI:0.95-0.99) and PGF (OR:0.96, 95%CI:0.94-0.98) with PC risk (**Error! Reference source not found.**).”

and added references to the relevant **Supplementary Materials** (e.g., **Sections S4.1** and **S4.2**), particularly for the cross-cancer analysis.

8. *The study applies multiple testing corrections (ENT and Bonferroni correction), but the results are not consistently presented in tables or results section. It is also unclear what significance threshold was used for both multiple testing corrections.*

   Response: Thank you for your comment. We used the Effective Number of Tests (ENT) method to adjust for multiple testing in all observational analyses, including EPIC, UK Biobank (UKBB), the meta-analysis, and subgroup analyses. This is mentioned in both the abstract (“…the effective number of tests (ENT) was used to control for multiple testing”) and the Methods section (“We considered any association with a p-value below 0.05 divided by the effective number of tests (ENT) as statistically significant”). In the revised version, we have added a dedicated section titled "Multiple Testing Correction in Observational Studies” (**page 8, lines 279-285**) to clearly describe the approach and threshold used (0.05/189).

   For the MR analyses, we applied Bonferroni correction and this is described in the text on **page 10, lines 331**:

   > “To account for multiple testing in our MR analyses, a Bonferroni-corrected threshold of 0.05/285 was used based on the number of proteins with available cis-instruments (i.e. 285 proteins)”

   For the Exome Analysis, **page 9, lines 298-301**:

   > “For the exome analysis, conventional significance was defined as p< 0.05, while Bonferroni correction was used as the threshold for multiple test correction across the number of proteins investigated (225) for cis-pQTL and combined (314) exGS analyses.”

9. *Table 2 only presents the statistically significant associations, but it would be valuable to include all 368 protein associations in the Supplementary Material to improve transparency.*

   Response: Thank you for the reviewer’s comment. The results for all protein-cancer associations are provided in the **Supplementary Tables**: observational analyses (**Table S5**), cis-pQTL analyses (**Table S7**), exome analyses (**Table S8**), and Mendelian randomization (MR) analyses (**Table S9**). We have revised the main text to include references to **Supplementary Table S5** (**page 12, line 398**), **Supplementary Table S7** (**page 9, line 303**), **Supplementary Materials** section **S4.1** (**page 14, line 470**) and **Supplementary Materials** section **S4.2** (**page 14, line 474**).

10. *The study reports protein-protein correlations but does not provide correlation coefficients or statistical significance. If the proteins are highly correlated, mutual adjustment may introduce multicollinearity, making effect estimates unstable. The authors could consider checking for multicollinearity using variance inflation factor.*

    Response: Supplementary Figures S2 and **S3** present both the pairwise correlation coefficients between proteins and their corresponding significance levels (*p < 0.05; **p < 0.01; ***p < 0.001).

As described in the manuscript, the main analyses evaluate the association of each protein and prostate cancer risk individually, without mutual adjustment for other proteins ("Conditional logistic regression was used to estimate odds ratios (ORs) and 95% confidence intervals (CIs) for the association of each circulating protein concentration with risk of PC") [**page 7, lines 240-242**].

The section titled "Correlations between and mutual adjustment for selected proteins" is included to provide additional information about the level of inter-correlation among selected proteins. While all pairwise correlations were below 0.6, which suggests only moderate correlation, we also note that the corresponding variance inflation factor (VIF) for this level of correlation would be approximately 1.57 ($VIF = 1 / (1 - r^2)$), which is well below conventional thresholds for multi-collinearity concerns (typically VIF > 5 or 10).

Beyond statistical stability, examining protein-protein correlations and potential interactions is also important for biological interpretation. Correlated proteins may act within shared pathways or biological processes, and understanding these relationships can help identify candidate pathways underlying prostate cancer risk. To support this interpretation, we complemented the correlation analysis with protein–protein interaction network analysis (**Supplementary Materials**, Section **S3**), offering additional insights into how these proteins may jointly contribute to prostate cancer development.

11. *The study identifies PARP1 as significantly associated with PC risk in the exome analysis, but this finding was not supported in observational or MR analyses. It would strengthen the discussion to address potential reasons for this discrepancy, such as possible reverse causation in observational analysis, differences in the genetic architecture captured by exome analysis vs MR analysis. Additionally, several proteins were identified in only one type of analysis e.g. observational, exome, or MR/colocalization. Providing a more detailed discussion on the interpretation of these differences – including possible biological, technical, or statistical explanations – would help understand the findings and clarify their implications*

Response: We agree that there is some inconsistency between the association that we identify and externally replicated in the MEC, and the results from other methods in this manuscript. We note that there is directionally consistent but non-significant evidence for higher PARP1 associated with increased prostate cancer risk from our cis pQTL MR analysis. Genetic analyses are often framed as modelling the lifelong effect of an elevated exposure. In this case, the effect size for PARP1 implied by our exome analyses is relatively modest (5% increased risk per SD increase in PARP1) and we were likely underpowered with our observational data to observe these presumed lifelong effects which may be revealed in future studies with larger sample sizes.

In the case of not observing genetic evidence for proteins we identified for observational data, we agree this is the subject of much interest and we have discussed this in the Discussion, including for example for SPINT2 on **page 16, lines 544-550**:

> "Similarly, further work is needed to understand the associations of SPINT2 and NME3 with PC; while both were associated with an increased risk using MR, results from observational analyses suggested this association may be inverse. One explanation for this may be potential complexities arising from cis pQTL used to proxy protein levels; cis pQTL for SPINT2 were also splicing QTL with the protein increasing allele also being associated with high intron-excision ratio, which makes it challenging to ascertain whether MR analyses proxy relative binding affinity for these Olink assays to one over other proteoforms or overall protein abundance."

And also on **page 16-17, lines 581-587**:

"Limited or no support from MR and colocalisation analyses may also be due to other factors such as case mix in the cancer GWAS and genomic complexity of cis-protein regions, which remain challenging for colocalisation methods to resolve. Discrepancies between results from MR and exome analyses may stem from differences in the genetic variants that contribute to these methods; MR typically leverages largely intronic variants that may determine protein levels by altering the promotion or enhancement of transcription while exome variants may predict the abundance of differing functional protein sequences in the blood."

12. *SPINT2 was identified as candidate causal protein based on MR and colocalization analyses, but the colocalization results (Table S10) do not support a shared causal variant. The PP4 value was close to 0, while the PP3 was close to 1, suggesting that SPINT2 and PC are likely driven by distinct genetic signals rather than true colocalization.*

Response: We observed multiple potential causal variants using the Susie method at the SPINT2 locus. Using this method we observed high PP4 for between SPINT2 and prostate cancer at this cis locus using rs7253245 (PP4: 0.97 and PP3=0.024). These results are presented in **Supplementary Table S10**.

Reviewer #4 (Remarks to the Author):

Reviewer comments for NMED-A138357

General Comments:

This manuscript presents an extensive statistical analysis investigating the role of inflammatory and immune-related proteins in prostate cancer (PC) risk using observational, genetic, and Mendelian randomization (MR) approaches. The study is well-structured, leveraging data from multiple large-scale cohorts (EPIC, UKBB, MEC, and PRACTICAL) and incorporating robust statistical methodologies. However, several points warrant clarification and further consideration.

Specific Comments:

1. *Could the authors provide details on the methods used to evaluate the proportional hazards assumption? This information would help in assessing the robustness of your Cox regression model results.*

Response: We evaluated the proportional hazards (PH) assumption using Schoenfeld residuals and found evidence of violation for 12 proteins at conventional significance levels. To address this, we conducted additional analyses incorporating a time-varying covariate (TVC) for these proteins. Only IL17A met the ENT threshold, with higher levels associated with a lower risk of prostate cancer in UK Biobank; an association not observed in EPIC. For IL15, the p-value decreased notably in the TVC analysis (PH p = 0.02 vs. TVC p = 0.007), consistent with the stronger protective effect seen in the long-lag analysis described in the paper. For DNER, the TVC model improved the p-value but did not meet the ENT threshold in UK Biobank and showed no supporting evidence in EPIC. Results for the remaining nine proteins were essentially unchanged. We have added these methodological details to the Methods (**page 8, lines 258–260**):

> "The proportional hazards (PH) assumption was evaluated using Schoenfeld residuals; where violations were identified, extended Cox models using time-varying effects for proteins were fitted."

and Results (**page 11, lines 372–376**):

> Evidence of PH assumption violations was identified for 12 proteins using Schoenfeld residuals. For these proteins, extended Cox analyses revealed a significant association of higher IL17A with lower prostate cancer risk in the UKBB; no such association was observed in EPIC. For IL15 and DNER, p-values improved but did not meet the ENT threshold, and results for the remaining nine proteins were materially unchanged.

Figure x: Time-varying hazard ratio for selected proteins with 95% confidence interval in the UK Biobank

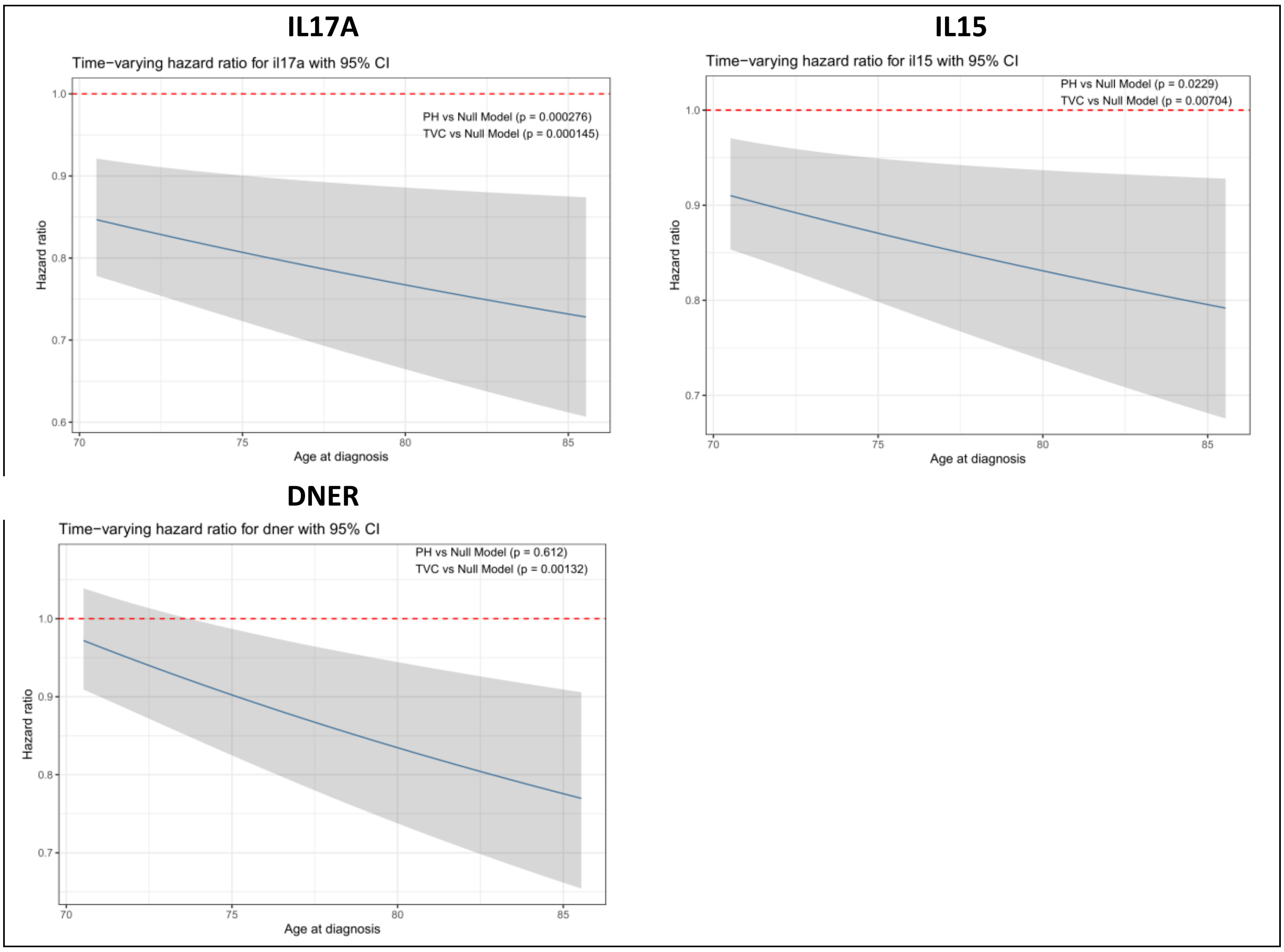

2. *The authors used the effective number of tests (ENT) for multiple testing correction. While this is a reasonable approach, can the authors provide a justification for using ENT instead of stricter family-wise error rate (FWER) controls?*

   Response: Thank you for this helpful comment. We used the effective number of tests (ENT) approach to correct for multiple testing, as it accounts for the correlation structure among the tested proteins and provides a balance between controlling false positives and retaining statistical power. While stricter family-wise error rate (FWER) methods such as Bonferroni correction are more conservative, they assume independence between tests and may be overly stringent in high-dimensional omics data where substantial correlation exists among features. We have now added the following sentence to the methods section on **page 8, lines 279-282** to justify our choice:

   > "To account for multiple testing, we applied the Effective Number of Tests (ENT) approach, which reflects the correlation structure among proteins and provides a less conservative correction than Bonferroni while still addressing the risk of false discoveries."

3. *The selection criteria for proteins in subgroup analyses should be clarified. Were all proteins tested in stratified models, or only those significant in the primary analysis?*

   Response: Thank you for the comment. All 368 proteins were included in the subgroup analyses, regardless of their significance in the primary analysis. We have clarified this point in the Methods section (**page 7, line 249**).

"These analyses were repeated for all proteins in subgroups of time from blood collection to diagnosis (<3 years [n=206], 3-7 years [n=311], and >7 years [n=917]), age at diagnosis (≤67 years/> 67 years), and prostate tumour stage (localised/advanced), histological grade (low-intermediate/high), and aggressiveness (aggressive/non-aggressive)."

4. *The authors mention Bayesian colocalization to confirm shared causal variants. Since colocalization does not always confirm causality, did the authors check for LD structure or perform alternative instrumental variable selection strategies?*

   Response: We thank the reviewer for raising this important point. Beyond our Bayesian colocalization analysis, we applied SuSiE fine-mapping (Wang et al., 2020) to each cis-pQTL region, explicitly modelling multiple causal variants and local LD structure. SuSiE produces credible sets of jointly associated variants, from which it selected conditionally independent instruments even in complex LD blocks. We then repeated colocalization using the SuSiE-derived signals, which is more robust to LD confounding. We agree that no single analysis can definitively establish causality. Instead, we believe that our complementary strategy, integrating prospective cohort data with orthogonal genetic methods, provides convergent evidence to guide future experimental studies which may highlight likely causal pathways in prostate cancer aetiology.

5. *The models adjusted for standard confounders (age, BMI, smoking, education, etc.), but were inverse probability weighting or other methods considered?*

   Response: Thank you for your suggestion. Inverse probability weighting (IPW) is particularly useful when confounding is strong and not adequately addressed by standard covariate adjustment. In our study, we adjusted for protentional confounders such as exact age, BMI, smoking, alcohol intake, education, and diabetes status. Among these, age is the primary potential confounder, but cases and controls were matched with controls on age within six months, minimising residual confounding. Other covariates, such as BMI and diabetes, showed only moderate associations with prostate cancer and did not exhibit strong imbalances (see **Table 1**).

   While IPW can offer additional robustness, it also introduces potential limitations, including sensitivity to model misspecification and extreme weights. Given the characteristics of our data, we considered standard adjustment appropriate for our primary analyses. We agree, however, that IPW or similar approaches may be valuable in future sensitivity analyses.

6. *Were interactions tested between key proteins (e.g., IL15, FLT3LG) and known PC risk factors, such as genetic risk scores for PC?*

   Response: Investigating interactions between key proteins (e.g., IL15, FLT3LG) and established prostate cancer risk factors, such as 451 polygenic risk scores and other rare variants like BRCA2, is an important next step, but is beyond the scope of the current manuscript.

7. *The paper suggests that higher levels of immunosurveillance-related proteins are protective, while higher PARP1 increases risk. Given that observational and genetic estimates for some proteins were discordant, can more discussion on possible explanations be provided?*

   Response: We agree that there is some inconsistency between the association that we identify and externally replicated in the MEC, and the results from other methods in this manuscript. We note that there is directionally consistent but non-significant evidence for higher PARP1 associated with increased prostate cancer risk from our cis pQTL MR analysis. Genetic analyses are often framed as modelling the lifelong effect of an elevated exposure. In this case, the effect size for PARP1

implied by our exome analyses is relatively modest (5% increased risk per SD increase in PARP1) and we were likely underpowered with our observational data to observe these presumed lifelong effects which may be revealed in future studies with larger sample sizes.

In the case of not observing genetic evidence for proteins we identified for observational data, we agree this is the subject of much interest and we have discussed this in the Discussion, including for example for SPINT2 on **page 16, lines 544-550**:

> "Similarly, further work is needed to understand the associations of SPINT2 and NME3 with PC; while both were associated with an increased risk using MR, results from observational analyses suggested this association may be inverse. One explanation for this may be potential complexities arising from cis pQTL used to proxy protein levels; cis pQTL for SPINT2 were also splicing QTL with the protein increasing allele also being associated with high intron-excision ratio, which makes it challenging to ascertain whether MR analyses proxy relative binding affinity for these Olink assays to one over other proteoforms or overall protein abundance."

And also on **page 16-17, lines 581-587**:

> "Limited or no support from MR and colocalisation analyses may also be due to other factors such as case mix in the cancer GWAS and genomic complexity of cis-protein regions, which remain challenging for colocalisation methods to resolve. Discrepancies between results from MR and exome analyses may stem from differences in the genetic variants that contribute to these methods; MR typically leverages largely intronic variants that may determine protein levels by altering the promotion or enhancement of transcription while exome variants may predict the abundance of differing functional protein sequences in the blood."

8. *The statement that IL15 and FLT3LG could be targets for PC prevention is intriguing but should be phrased cautiously.*

   Response: We agree that at this point cautious optimism is warranted. Our interpretation is limited to what we report in the current manuscript and previous evidence for the importance of IL15 and FLT3LG pathways in prostate cancer. For example, our observational evidence is consistent with involvement of the IL15–FLT3LG axis in prostate cancer aetiology and functional and experimental evidence describe the role of IL15 is reducing tumour progression phenotypes. However, we agree that further replication and functional validation is necessary. We therefore restrict our conclusions to suggesting that IL15-FLT3LG related biological process like immunosurveillance, be priorities for further research, which may in time inform prevention strategies.

9. *While the authors attempt replication in MEC, it is unclear how well findings generalize to non-European populations.*

   Response: We thank the reviewer for raising this important point. As mentioned in the discussion, the majority of our genetic analyses were conducted in individuals of European ancestry. Nonetheless, we were able to replicate our key finding for PARP1 and prostate cancer risk in the Multiethnic Cohort (MEC), which included participants from several non-European populations (White: 555 cases; Latin American: 602; Japanese: 1,006; Black: 538; Hawaiian: 182). This suggests that, at least for this locus, the signal observed in the UK Biobank is consistent across ancestries. However, we also note that the number of non-European cases remains relatively limited, and only a few large-scale datasets to date have directly measured plasma protein levels in diverse

populations. Expanding such resources will be an important priority for future research. We discuss replication of PARP1 in the MEC in the Discussion on **page 16, lines 552-555**:

> "We also identified genetically predicted higher PARP1 to be associated with an increased risk of PC overall using exome protein score analyses and were successful in replicating this association in an external, diverse prospective cohort consisting of African American, European American, Japanese American, Latino, and Native Hawaiian men."

and **page 16, lines 567-571**:

> "However, we note that the data in this analysis were majority participants in EPIC that are predominantly of European ancestry and that almost all cases in our UK Biobank analyses are also of European ancestry; the promise of these initial proteomics results further underscores the need for the prompt diversification of studies on proteins and cancer and the assessment of the generalisability of findings across populations with differing ancestries."

10. *The sample sizes for aggressive PC and >7 years analyses are smaller. Could power limitations explain the null findings for some proteins in aggressive disease?*

Response: Thank you for the comment. We agree that the statistical power is more limited in the subgroup analyses because of the smaller number of cases, leading to wider confidence intervals and greater likelihood of findings not passing the significance threshold. With 1,434 cases overall, we would have 76% power to detect a relative risk of 0.85 for prostate cancer, whereas for aggressive disease, with 488 cases, the power would be only 13% ($\alpha = 0.05/189$). As shown in Figure 3, FLT3LG, PGF, and BCL2L11 were associated with aggressive disease at conventional significance levels, and IL15 and FLT3LG also showed protective associations with advanced stage. These findings are directionally consistent with the overall analysis but were not sufficiently powered in the subgroup analysis. We have revised the Discussion on **page 17, lines 594-597** to comment on this limitation.

> "Finally, number of cases was limited for PC subtypes and for some cancers included in our cross-cancer analysis, leading to more limited statistical power to detect associations between protein level and risk of these specific cancer endpoints."

This is a well-conducted study employing rigorous statistical methods to investigate the role of immune-related proteins in PC risk. Addressing the points above would strengthen the manuscript's conclusions.
